# RadH: A Versatile Halogenase for Integration into Synthetic Pathways

**DOI:** 10.1002/anie.201706342

**Published:** 2017-08-18

**Authors:** Binuraj R. K. Menon, Eileen Brandenburger, Humera H. Sharif, Ulrike Klemstein, Sarah A. Shepherd, Michael F. Greaney, Jason Micklefield

**Affiliations:** ^1^ School of Chemistry & Manchester Institute of Biotechnology The University of Manchester 131 Princess Street Manchester M1 7DN UK

**Keywords:** biocatalysis, directed evolution, enzyme mechanisms, halogenases, pathway engineering

## Abstract

Flavin‐dependent halogenases are useful enzymes for providing halogenated molecules with improved biological activity, or intermediates for synthetic derivatization. We demonstrate how the fungal halogenase RadH can be used to regioselectively halogenate a range of bioactive aromatic scaffolds. Site‐directed mutagenesis of RadH was used to identify catalytic residues and provide insight into the mechanism of fungal halogenases. A high‐throughput fluorescence screen was also developed, which enabled a RadH mutant to be evolved with improved properties. Finally we demonstrate how biosynthetic genes from fungi, bacteria, and plants can be combined to encode a new pathway to generate a novel chlorinated coumarin “non‐natural” product in E. coli.

Flavin‐dependent halogenase (Fl‐Hal) enzymes halogenate aromatic precursors in the biosynthesis of a diverse range of halogenated natural products, including antibiotics, antitumor agents, and other bioactive compounds.^1^ The halogen substituents installed by these enzymes are often important for the bioactivity of the natural products and can also be used as an orthogonal handle for further synthetic derivatization.^2^ Tryptophan halogenases (Trp‐Hal) from bacteria are the most well characterized Fl‐Hal enzymes and have been subject to detailed structural and mechanistic investigations.^1^, ^3^ The regioselectivity and benign, aqueous operating conditions of Trp‐Hal enzymes has also provoked considerable interest in the development of these enzymes for synthetic applications.^4^ Many valuable materials, agrochemicals and ca. 30 % of the leading pharmaceuticals possess halogens.^5^ Haloaromatics are also widely used precursors and intermediates in synthesis. However, the narrow substrate specificity, relatively low activity, and poor stability of wild‐type Trp‐Hal enzymes limits their synthetic utility. Despite this, there has been progress in expanding the substrate scope, improving catalytic activity, and altering the regioselectivity of Trp‐Hal enzymes by using targeted or random mutagenesis approaches.^4^

Genome mining reveals there are many other bacterial Fl‐Hal enzymes.^6^ However, the majority of these remain uncharacterized and many are likely to require carrier‐protein‐tethered substrates, which would limit their utility. On the other hand, there are a number of fungal Fl‐Hal enzymes that halogenate phenolic intermediates, independent of carrier proteins, in the biosynthesis of natural products, including radicicol,^7^ griseofulvin,^8^ aspirochlorine,^9^ and chaetoviridins^10^ (Figure 1). To date the catalytic scope of these fungal halogenases has been little explored beyond the natural substrates.^11^ In this paper, we explore the mechanism and catalytic scope of the fungal halogenase RadH. We introduce a high‐throughput fluorescence screening method to rapidly select RadH mutants with improved properties, and demonstrate how an improved RadH variant can be integrated into an engineered pathway to deliver novel halogenated “non‐natural” products.

**Figure 1:**
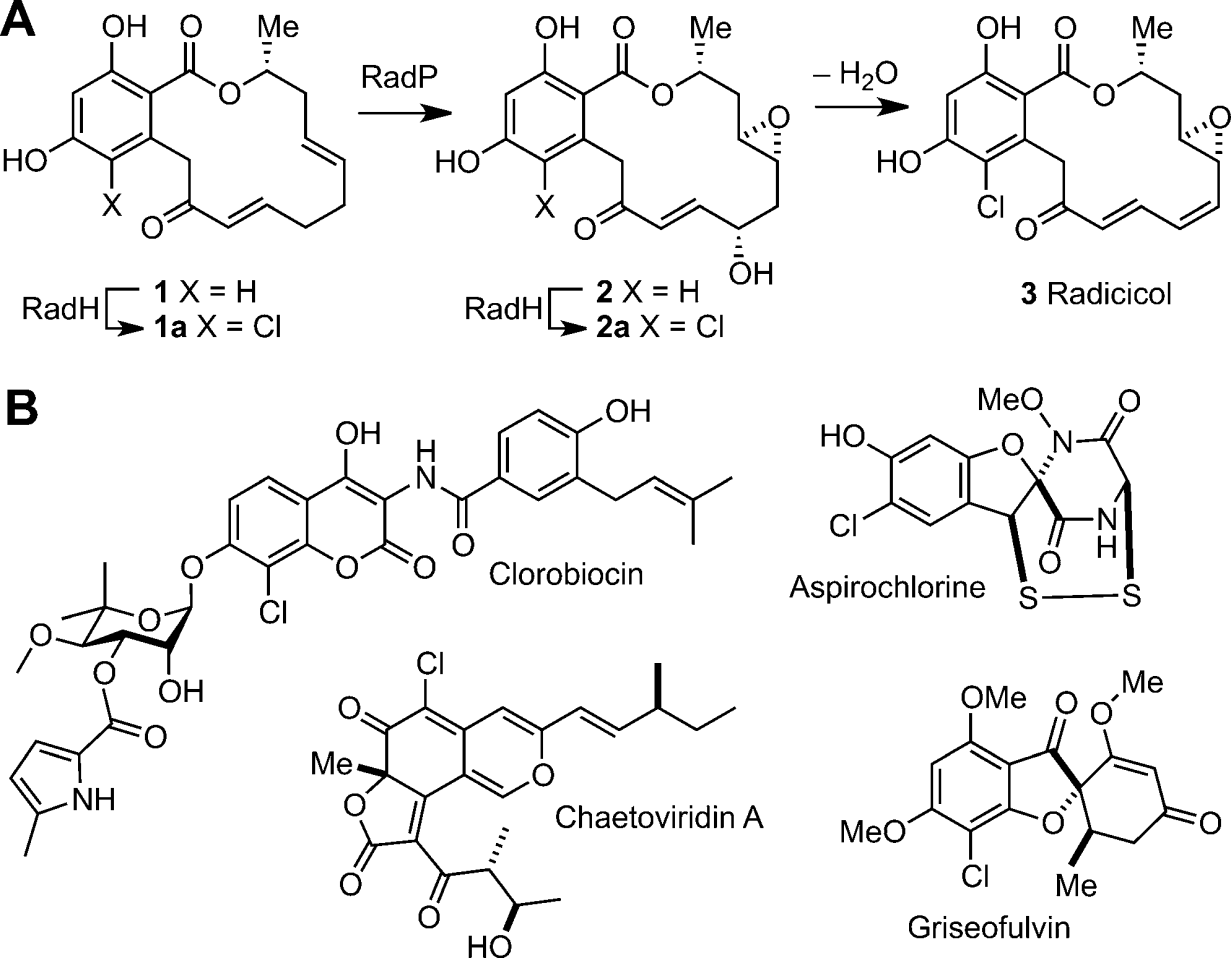
A) Proposed final steps in the biosynthesis of radicicol. B) Other natural products derived from Fl‐Hal chlorination of phenolic precursors.

Previous gene‐deletion experiments indicate that RadH is required for the production of radicicol in the fungus *Chaetomium chiversii*.^12^ Sequence similarity (Figure S1 in the Supporting Information) suggests that RadH is likely to fulfill the same function as Rdc2, which has been shown to catalyze the chlorination of monocillin II (**1**) to give **1 a** in the biosynthesis of radicicol (**3**) in a different fungal strain (Figure 1).^7^ We overproduced RadH in *E. coli*, and as expected, RadH does accept monocillin II as a substrate. However, the halogenation of **1** to **1 a** was slow, reaching only 84 % conversion after 18 hours. In addition, unlike Rdc2,^7^ RadH does not halogenate the related natural products zearalenone (**4**) or curvularin (**5**), thus suggesting that despite close sequence similarity (87 %), RadH and Rdc2 exhibit different active‐site architectures.

To further explore the biocatalytic potential of RadH, a number of aromatic compounds were tested as potential substrates (Figure 2 and Figures S2, S3). This showed that RadH is more promiscuous than other Fl‐Hal enzymes. RadH halogenates a range of natural and synthetic phenolic compounds (Figure 2 and Figure S3). Interestingly, RadH halogenates several of these compounds with higher efficiency than the proposed natural substrate **1**.^7^, ^12^ Of the compounds tested, 6‐hydroxyisoquinoline (**6**) and the plant natural product 7‐hydroxycoumarin (**8**) are the best substrates for RadH (Figure 2). In addition, RadH also halogenated plant derived flavonoids (e.g., **12**, **13**, and **14**). In all cases, we observe regioselective halogenation *ortho* to the phenolic hydroxy group, and for the majority of bicyclic or tricyclic substrates where there are two possible *ortho* positions that could be halogenated, RadH regioselectively halogenates proximal to the bridging position. To explore whether the phenolic moiety is required for RadH activity, we tested a range of electron‐rich aromatic compounds that lack hydroxy substituents and found that none of these were substrates (Figure S2). In addition, compounds with methoxy instead of hydroxy groups proximal to the halogenation site, including the methoxy derivatives of substrates **6, 8**, and **12**, were not halogenated by RadH despite having similar overall structure and electronic properties (Figure S2). This suggests that deprotonation of the phenol hydroxy group is essential in the mechanism of RadH and contributes to the regioselectivity of halogenation (Figure 3). In contrast to the highly regioselective RadH, conventional (non‐enzymatic) halogenation of phenols usually gives rise to mixtures of *ortho*‐, *para*‐, and di‐substituted products. The kinetic parameters for RadH with selected substrates were determined (Table 1 and Figures S4–S10). Notably, the *k*_cat_ values for RadH with iso‐quinoline **6** and coumarin **8** were significantly higher than those reported for Trp‐Hal enzymes with the natural substrate.3a, 13 Whilst kinetic analysis shows that monocillin II (**1**) is turned over more slowly than the other substrates, *k*_cat_/*K*_m_ was not determined for **1** since saturation of the Michaelis–Menten curve was not attained.

**Figure 2:**
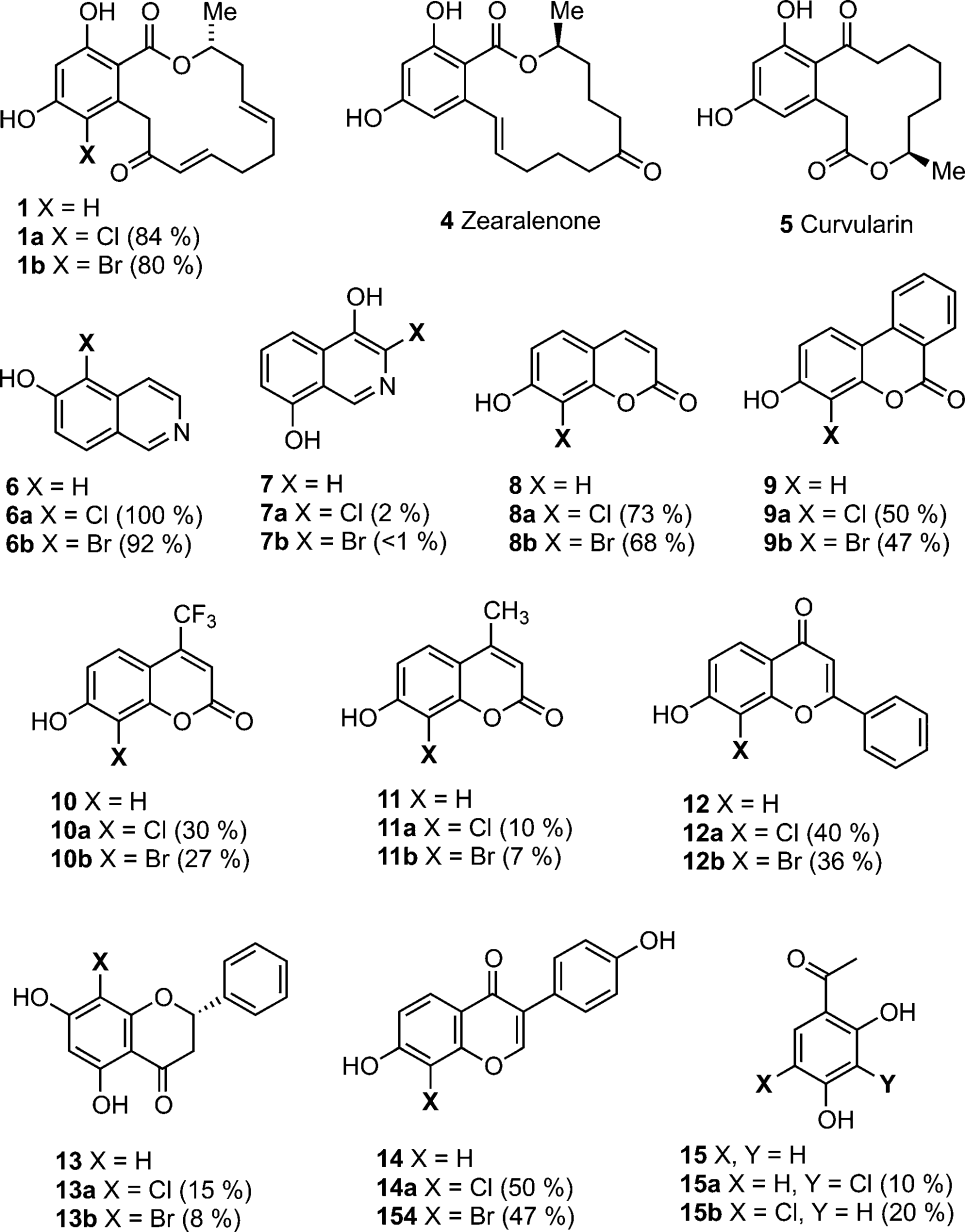
RadH substrate scope. Conditions: RadH (15 μm), Fre (2.5 μm), FAD (1 μm), NADH (2.5 mm), MgCl_2_ (or KBr for bromination reactions; 10 mm), and substrate (0.5 mm) in 10 mm potassium phosphate buffer at pH 7.4. Reactions were analyzed by HPLC after 2 hour incubation at 30 °C and 200 rpm. For reactions with monocillin II, higher enzyme concentrations [RadH (100 μm) and Fre (20 μm)] were used and the conversions were measured after 18 hours. Fre=flavin reductase, FAD=flavin adenine dinucleotide, NADH=reduced nicotinamide adenine dinucleotide.

**Table 1:** Kinetic parameters for wild‐type and D456E/T501S RadH.

RadH	Substrate	*k*_cat_ [min^−1^]	*K*_m_ [μm]	*k*_cat_/*K*_m_ [min^−1^ μm]×10^−3^
WT	**6**	3.2±0.2	277±3	12±0.7
	**8**	2.9±0.4	379±5	7.7±1.1
	**10**	0.65±0.04	309±19	2.1±0.2
	**11**	0.56±0.01	364±18	1.5±0.08
	**12**	0.032±0.003	347±2	0.092±0.009
	**14**	0.0054±0.002	281±2	0.019±0.007
				
D456E /T501S	**6**	5.8±0.6	268±10	22±2.4
	**8**	5.3±0.4	465±4	11±0.9
	**10**	ND	ND	ND
	**11**	ND	ND	ND
	**12**	0.031±0.002	383±4	0.081±0.005
	**14**	0.009±0.001	315±1	0.029±0.003

Many isoquinolines, coumarins, and flavonoids have been found to possess bioactivity for pharmaceutical and other applications (Figure S11). For example, the antibiotics clorobiocin and simocyclinone (Figure 1 and Figure S11) both possess the 8‐chloro‐7‐hydroxycoumarin moiety (**8 a**), with the 8‐chloro substituent contributing to antimicrobial activity.^14^ Given that RadH can halogenate common pharmacophores, we sought to engineer RadH variants with improved properties that might be used to generate new halogenated products with altered bioactivity, or intermediates for further synthetic elaboration.

To enable the screening of a larger number of RadH mutants, a high‐throughput assay for RadH activity was developed that exploits the fluorescence of 7‐hydroxycoumarin derivatives.^15^ The UV absorbance and fluorescence properties of coumarin substrates and halogenation products from RadH reactions were determined (Figures S12, S13). Chlorination of **8** to give 8‐chloro‐7‐hydroxycoumarin **8 a** resulted in the most significant change, with enhanced fluorescence emission at 456 nm upon excitation at 386 nm, which could easily be monitored in a 96‐well plate. To demonstrate that this assay can be used to select for improved RadH variants, a library of genes encoding RadH, with an average of 2–3 amino acid mutations per clone, was generated by error‐prone PCR (epPCR), and 960 colonies were picked then grown in 96‐well deep‐well plates. Crude cell lysates were then assayed for chlorination of **8**, and the 24 mutants with highest activity were further analyzed using ultra‐performance liquid chromatography (UPLC). The most promising mutant, D465E/T501S, showed a 15‐fold improvement in activity with **8** over wild‐type RadH in lysate assays (Figure S14). D465E/T501S was purified and shown to have significantly improved relative activity for a range of substrates (Table S1), as well as enhanced *k*_cat_ values with substrates **6**, **8**, and **14** compared to the wild‐type RadH (Table 1). To explain the more significant 15‐fold increase in activity observed in lysate assays with substrate **8**, protein production levels and the stability of D465E/T501S were compared with the wild‐type RadH. Whilst sodium dodecyl sulfate polyacrylamide gel electrophoresis (SDS‐PAGE) analysis of cell lysates indicated that the double mutant is produced to a significantly higher level in *E. coli* cells than the wild‐type enzyme (Figure S15), variable‐temperature CD indicated that the thermal stability of the wild‐type and mutant enzymes are similar (Figure S16). Thus, in addition to possessing improved catalytic activity (Table 1 and Table S1), the mutant RadH is more efficiently overproduced in *E. coli*.

In order to gain insight into the mechanism of RadH, and to rationalize how the D465E/T501S mutations may effect RadH structure and activity, a homology model for RadH was generated based on the X‐ray structure of a related flavoprotein (PDB ID: 3ATQ, Figure S17).^16^ The model shows RadH with three separate domains: the FAD‐binding, catalytic, and C‐terminal domains. The RadH catalytic domain has a large cavity (Figure 3 and Figure S18) near the isoalloxazine ring of FAD, which is likely to be the substrate binding site. To verify the model, individual lysine residues predicted to be close to (K73 and K74) or more distant (K80 and K84) to the putative substrate binding site were mutated. Whilst K73A, K80A, and K84A all showed activity similar to the wild‐type enzyme, the K74A mutation completely abolishes enzyme activity (Figure S19). The model predicts that K74 is around 4 Å away from the halogenation site (C6) of radicicol docked in the putative active site (Figure 3 and Figure S18). It is likely that the halide ion attacks C4a‐hydroperoxyflavin to generate hypohalous acid, which reacts with K74 to provide the key chloroamine or bromoamine electrophile.^3^ Interestingly, D465E and T501S are located distant from the putative active site, in the C‐terminal region of RadH, which may influence protein dynamic and interactions between the FAD binding and substrate binding domains.

**Figure 3:**
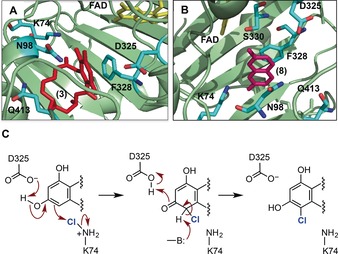
Active‐site models of RadH with radicicol (**3**; A) or with 7‐hydroxycoumarin (**8**; B) bound. C) Proposed mechanism for RadH halogenation.

The RadH structural model also shows the presence of aromatic residues F327 and F328 close to the substrate binding site, and indicates that F328 may be involved in π–π stacking interactions with the aryl group of the substrates. Accordingly, a F328A mutant was prepared (Figure S20) and was shown to have significantly reduced activity, which is consistent with F328 π‐stacking with the substrate. The model also suggests that there are several residues, such as N98, D325, S329, S330, and Q413, that are within H‐bonding distance of the substrate. To establish whether any of these residues may be involved in catalysis, the corresponding alanine mutants were created. Whilst all of the alanine mutants showed significantly reduced RadH activity, only D325A completely abolished the catalytic activity (Figure S20). This suggests that both D325 and K74 are essential for catalysis. Given that the model indicates that D325 would be in close proximity to the hydroxy group that is directly adjacent to the substrate halogenation site, it is possible that D325 may function as a general base to deprotonate the phenol group during or after attack of the electrophile (Figure 3 C). Presumably, another general base or possibly a water molecule is required to deprotonate the sp^3^‐hybridised center of the halogenated intermediate to facilitate rearomatization. This proposed mechanism (Figure 3 C) is also consistent with the observation that substrates that have been methylated at the hydroxy group proximal to the halogention site are not turned over by RadH (Figure S2B). Moreover, sequence alignments reveal that K74, D325, and F328 are conserved across known and putative fungal Fl‐Hal enzymes that also recognize phenolic substrates (Figure S21).

Previously, we showed how RadH can be integrated into synthetic pathways with chemocatalysts.^2^ To further demonstrate the versatility of RadH, we sought to integrate this enzyme into an engineered biosynthetic pathway to generate novel halogenated “non‐natural” products in vivo. The de novo biosynthesis of 8‐chloro‐7‐hydroxycoumarin (**8 a**) was selected as a target (Figure 4) because RadH can efficiently halogenate 7‐hydroxycoumarin (**8**; Figure 2), also known as umbelliferone, which is a central intermediate in the biosynthesis of coumarins produced by plants. Natural coumarins and synthetic derivatives have been developed as commercial fluorescent dyes and therapeutic agents (e.g., methoxsalen, which is used to treat psoriasis, the antibiotic novabiocin, and anticoagulants such as warfarin).^17^ To establish microbial production of 8‐chloro‐7‐hydroxycoumarin (**8 a**), a plasmid (RSFDuet‐1) with genes encoding 4‐coumaryl‐CoA ligase (4CL) from *Streptomyces coelicolor* 18 and feruloyl CoA 6′‐hydroxylase (F6′H) from the plant *Ipomoea batatas* 19 was generated and used to transform *E. coli* cells. Incubation of the transformant with precursor *p*‐coumaric acid (**16**) resulted in the production of 7‐hydroxycoumarin (**8**; Figure 4 and Figure S22). A second plasmid (pCDF) containing a gene encoding the RadH variant D465E/T501S, was next introduced and co‐expression of this halogenase, along with 4CL and F6′H, led to the production of 6.5 mg L^−1^ of **8** and 0.8 mg L^−1^ of 8‐chloro‐7‐hydroxycoumarin (**8 a**). Finally to demonstrate how **8 a** can be produced directly from glucose through fermentation, a third strain of *E. coli* was engineered with an additional plasmid (pACYC) encoding a tyrosine ammonia‐lyase (TAL) from *Saccharothrix espanaensis*.^19^ The TAL enzyme catalyzes the elimination of ammonia from the cellular pool of l‐tyrosine to generate *p*‐coumaric acid in vivo, which led to the production of 1.4 mg L^−1^ **8** and 1.1 mg L^−1^ **8 a** under fermentation conditions (Figure 4 and Figure S22). These titres could be increased by feeding l‐tyrosine. However, significantly higher levels of *p*‐coumaric acid were produced by the engineered *E. coli* strain than **8** or **8 a,** which may be due to higher production or activity of TAL compared with 4CL and F6′H.

**Figure 4:**
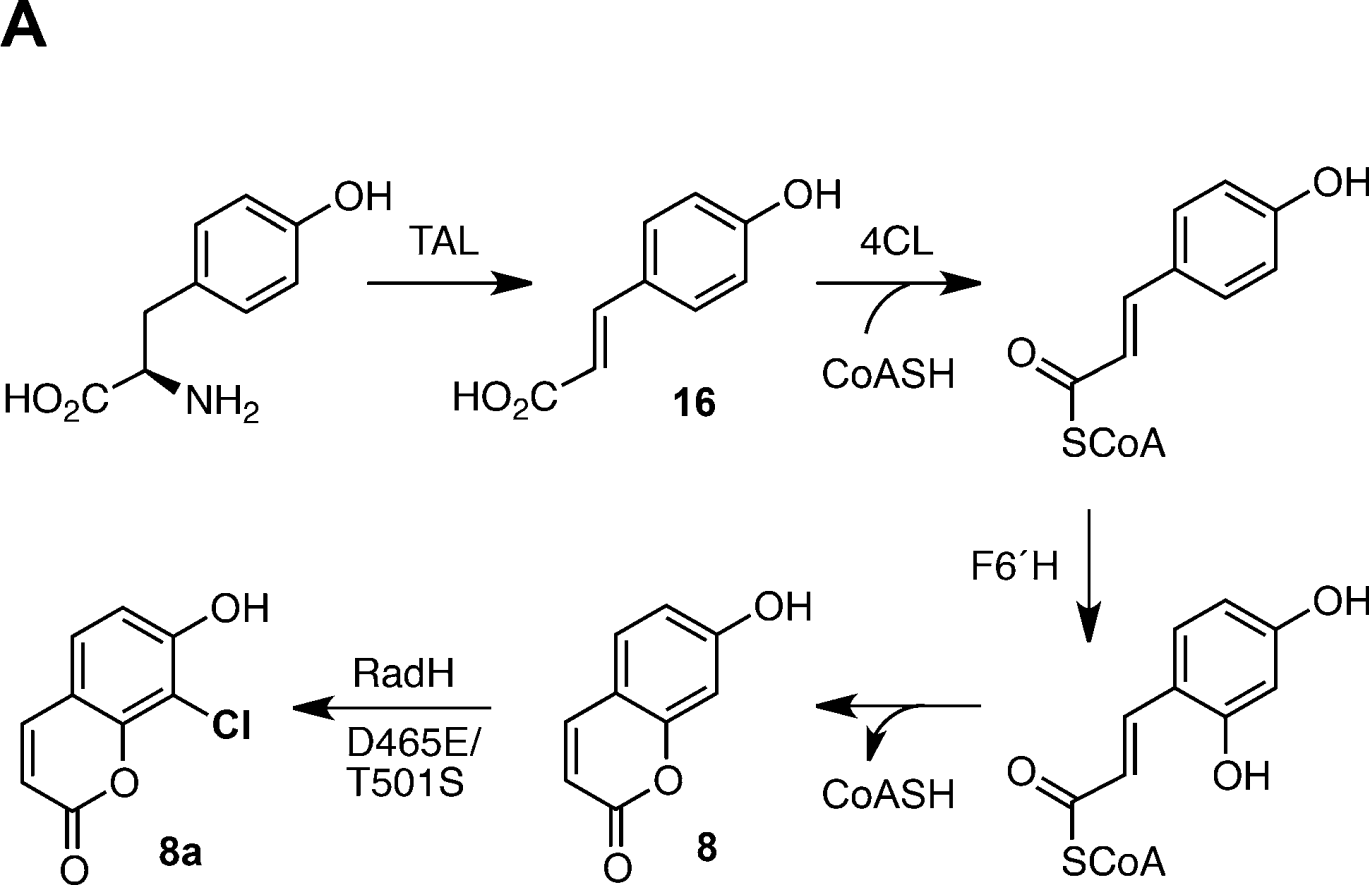
Engineered pathway to 8‐chloro‐7‐hydroxycoumarin (**8 a**) in *E. coli*.

Metabolic engineering approaches have previously been developed for the production of valuable natural plant coumarins in *E. coli*.^19^, ^20^, ^21^ However, as far as we are aware, this is the first example of the production of a “non‐natural” halogenated coumarin from glucose in *E. coli*. Notably, the engineered pathway to **8 a** is comprised of enzymes from plants, bacteria, and fungi. Production of **8 a** directly by fermentation also offers advantages over the traditional chemistry used to synthesize **8 a**, which requires deleterious and toxic reagents.^22^ Further optimization of this pathway and the addition of other enzymes from coumarin biosynthesis may also lead to additional halogenated derivatives with altered bioactivities. The C8 chloro group also provides an orthogonal handle for further synthetic derivatization.^2^

In summary, we have explored the biocatalytic and biosynthetic scope of the fungal halogenase RadH, showing that this enzyme is more promiscuous than other halogenases described to date. A phenolic hydroxy group is prerequisite for RadH activity, and halogenation is highly regioselective, occurring *ortho* to the phenol hydroxy group. Two active‐site residues, K74 and D325, were shown to be essential for RadH activity, and from this a mechanism is proposed (Figure 3) that is supported by the observation that compounds possessing methoxy groups in place of the substrate phenolic hydroxy group are not halogenated by RadH. In addition, we have developed a high‐throughput fluorescence assay, which was used to screen for a RadH mutant that exhibits higher activity. Deployment of the improved RadH variant into a coumarin biosynthetic pathway assembled in an *E. coli* host strain demonstrates that biosynthetic enzymes from diverse plant, bacterial, and fungal origins can be combined to create new pathways to novel halogenated ‘non‐natural“ products directly through fermentation.

## Conflict of interest

The authors declare no conflict of interest.

## Supporting information

As a service to our authors and readers, this journal provides supporting information supplied by the authors. Such materials are peer reviewed and may be re‐organized for online delivery, but are not copy‐edited or typeset. Technical support issues arising from supporting information (other than missing files) should be addressed to the authors.

Supplementary
