# Supplementary material for: RadH: A Versatile Halogenase for Integration into Synthetic Pathways
Source: Angew Chem Int Ed Engl. 2017 Aug 18;56(39):11841–5. doi: 10.1002/anie.201706342 (PMC5637929; doi:10.1002/anie.201706342)
Supplement: Supplementary file 1 — Supplementary [file ANIE-56-11841-s001.pdf]

Angewandte Chemie
Eine Zeitschrift der Gesellschaft Deutscher Chemiker

Supporting Information

RadH: A Versatile Halogenase for Integration into Synthetic Pathways

*Binuraj R. K. Menon[+], Eileen Brandenburger[+], Humera H. Sharif, Ulrike Klemstein, Sarah A. Shepherd, Michael F. Greaney, and Jason Micklefield**

anie_201706342_sm_miscellaneous_information.pdf

SUPPORTING INFORMATION

*

Content

Page S2-18: Supplementary Figures

Page S19-29: Experimental

Page S30-31: Supplementary References

Page S32-68: HPLC, NMR, UV and MS data for halogenase reaction products.

Supplementary Tables & Figures

	RadH WT	D465E/T501S	
Substrate	% Conversion	% Conversion	Fold improvement
**6**	28 %	79 %	2.8
**8**	18 %	34 %	1.9
**11**	2.5 %	3.5 %	1.4
**12**	10 %	14 %	1.4
**13**	3.8 %	7.2 %	1.9
**14**	12.5 %	29 %	2.3

Table S1. **Relative activities of purified enzymes D465E/T501S and wild-type RadH**. Assay conditions: RadH or D465E/T501S enzyme (15 µM), Fre (2.5 µM), FAD (1 µM), NADH (2.5 mM), $MgCl_2$ (10 mM) and substrate (0.5 mM) in a total volume of 200 µL in 10 mM potassium phosphate buffer, pH 7.4. The reaction was analyzed using HPLC after 30 minutes incubation at 30 °C.

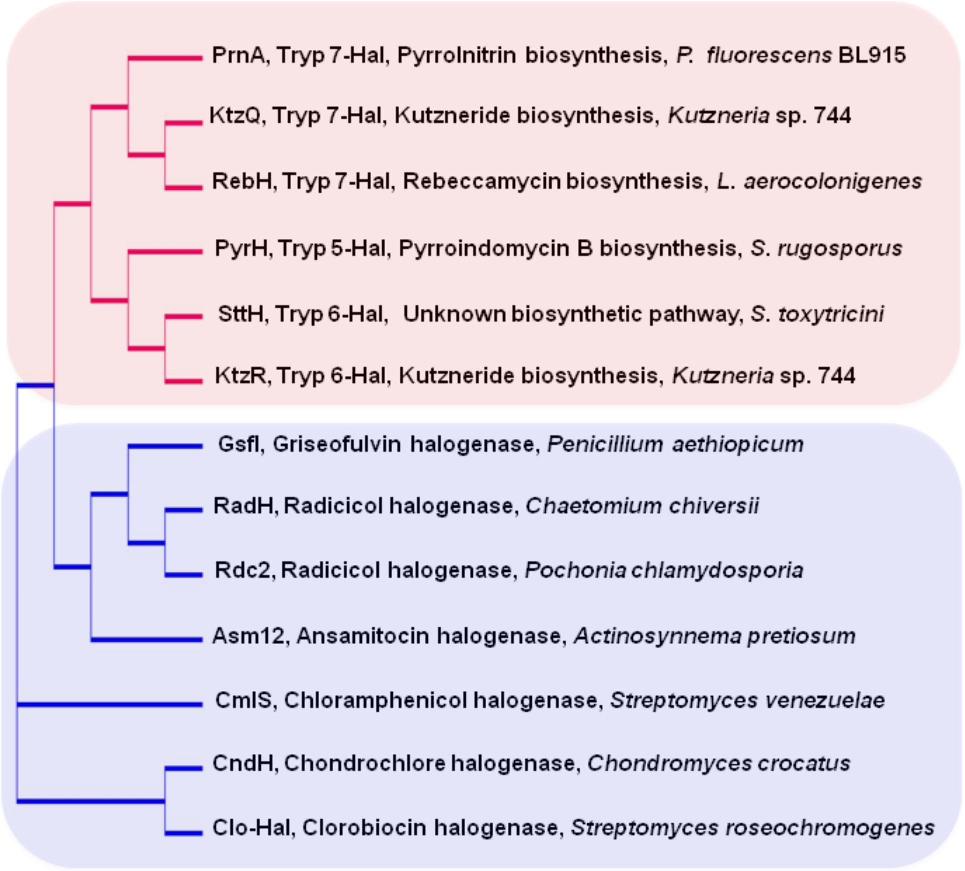

Figure S1. A phylogentic tree, generated using the neighbour-joining tree method without distance corrections via Dendroscope 3, showing the evolutionary relationship between flavin dependent tryptophan halogenases (red) and phenolic halogenases enzymes (blue).

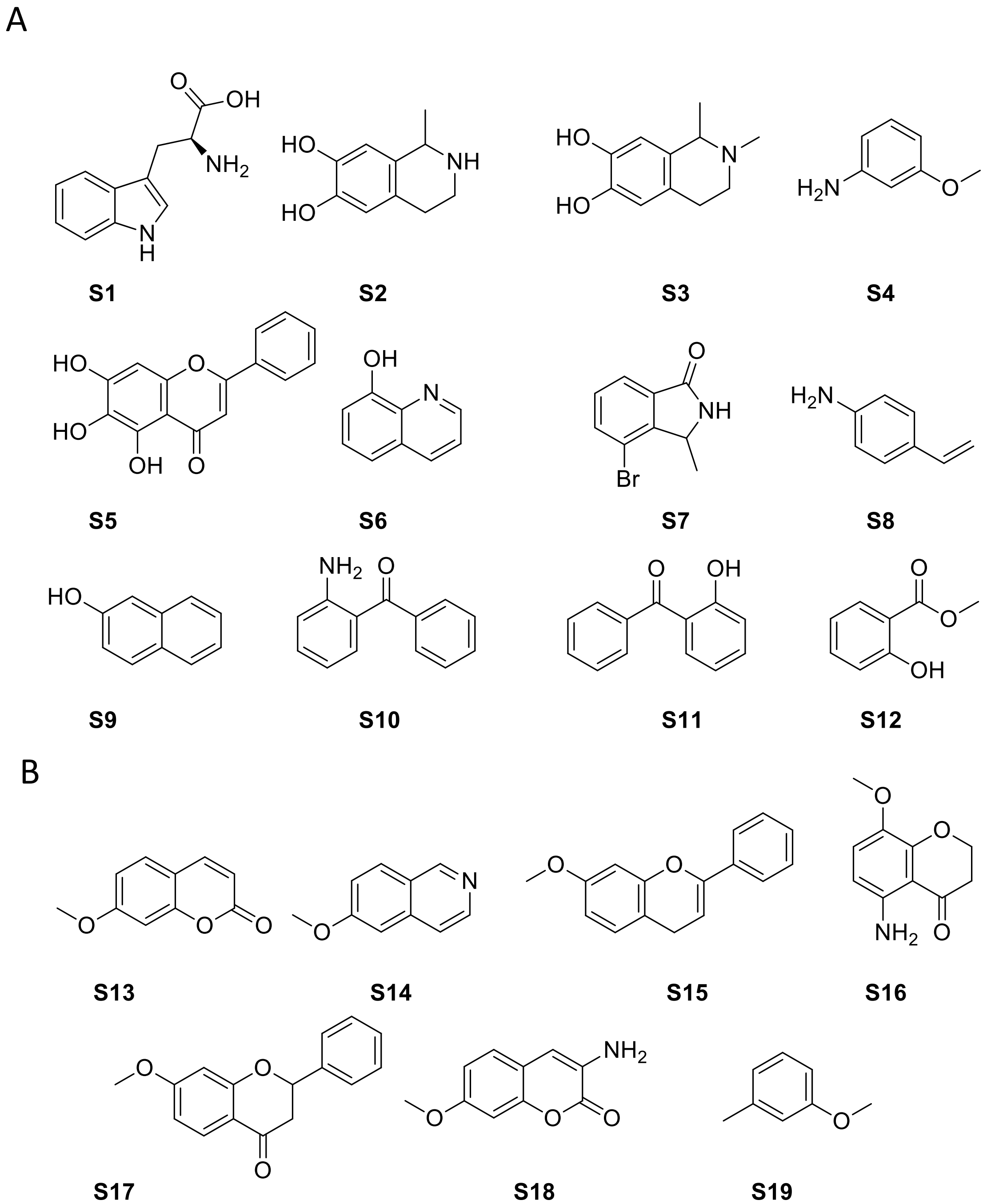

Figure S2. Display of compounds not accepted by RadH. (**A**) Amines and phenols (**S1**-**S12**); (**B**) methoxy compounds (**S13**-**S19**).

A

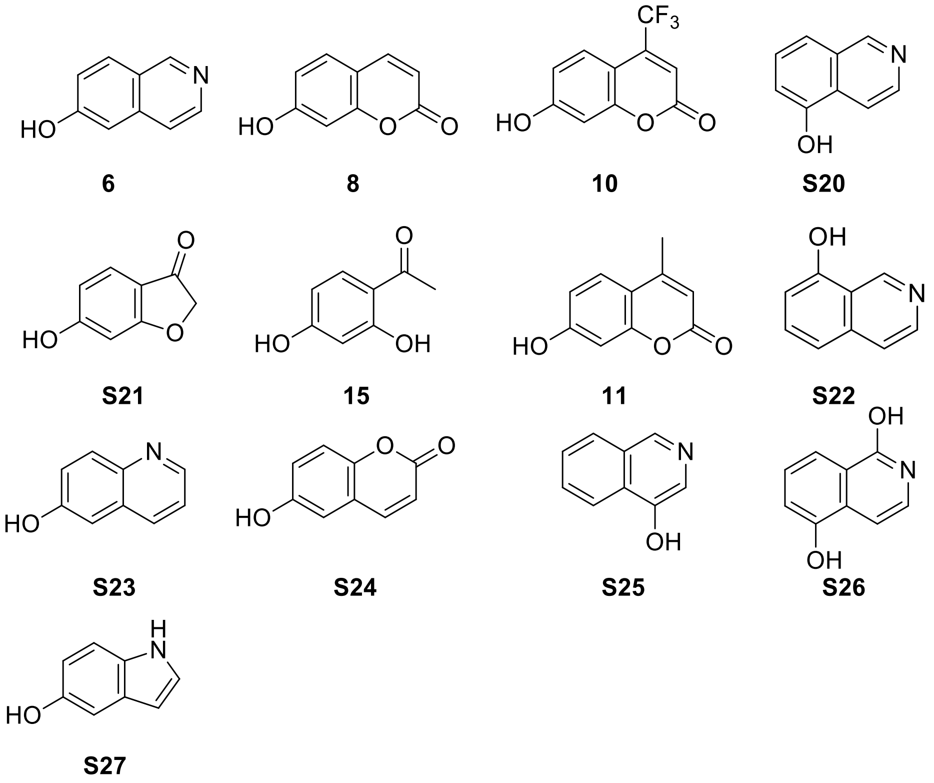

B

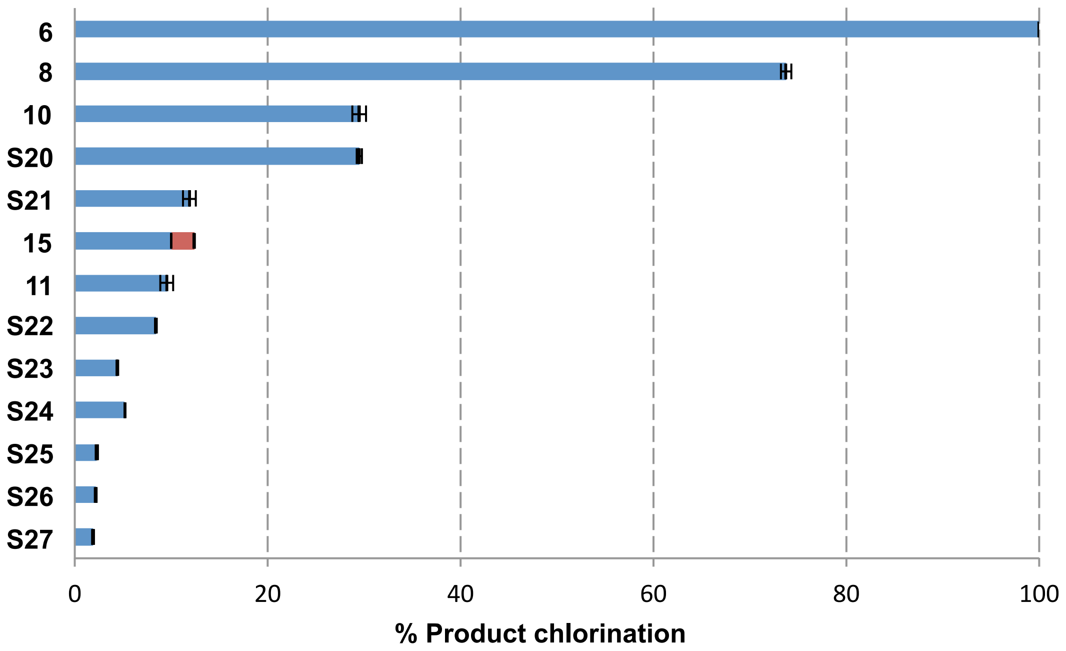

Figure S3. (**A**) Structures of RadH substrates. (**B**) Comparison of percentage conversion (chlorination) of tested substrates with RadH activity compounds from Figure 2 of the main text and additional substrates. Assay conditions were: RadH (15 µM), Fre (2.5 µM), FAD (1 µM), NADH (2.5 mM), $MgCl_2$ (10 mM) and substrate (0.5 mM) in a total volume of 200 µL in 10 mM potassium phosphate buffer, pH 7.4. The reaction was measured using HPLC after 2 hour incubation at 30 °C with shaking at 800 rpm as described previously.

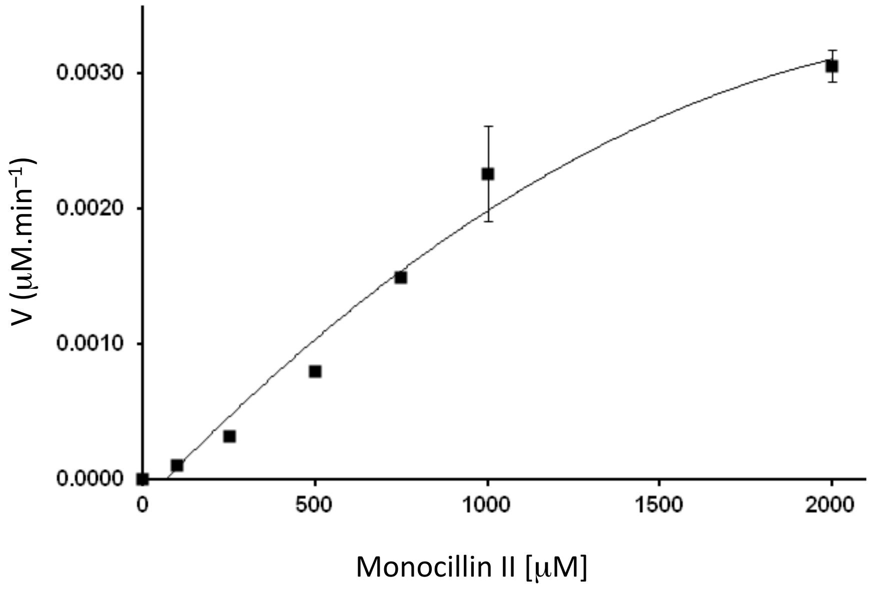

Figure S4. Substrate conversion graph of wild-type RadH chlorination of monocillin II **1**.

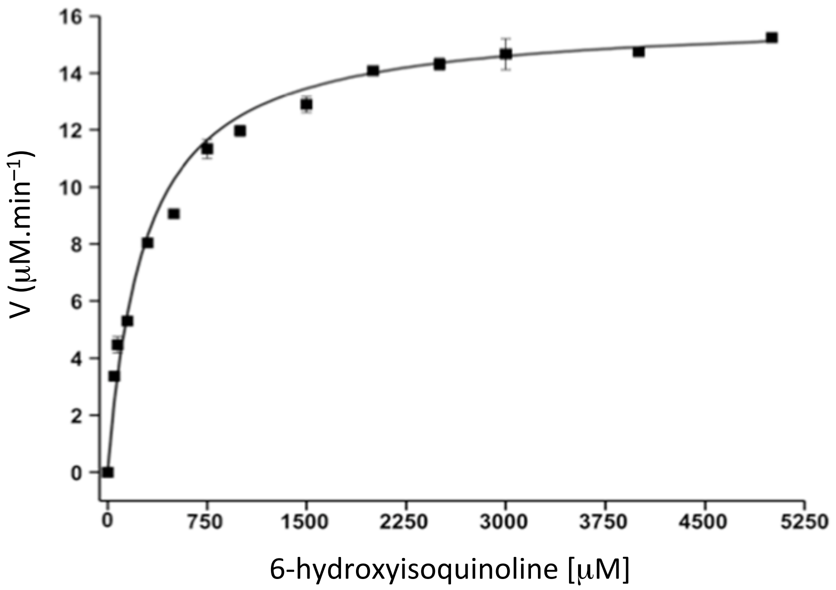

Figure S5. Michaelis-Menten graph of wild-type RadH chlorination of 6-hydroxyisoquinoline **6**.

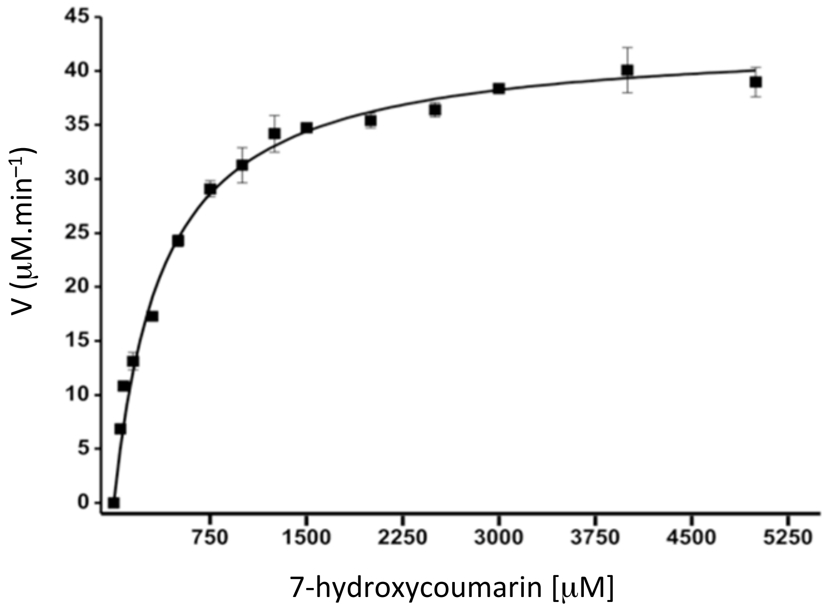

Figure S6. Michaelis-Menten graph of wild-type RadH chlorination of 7-hydroxycoumarin **8**.

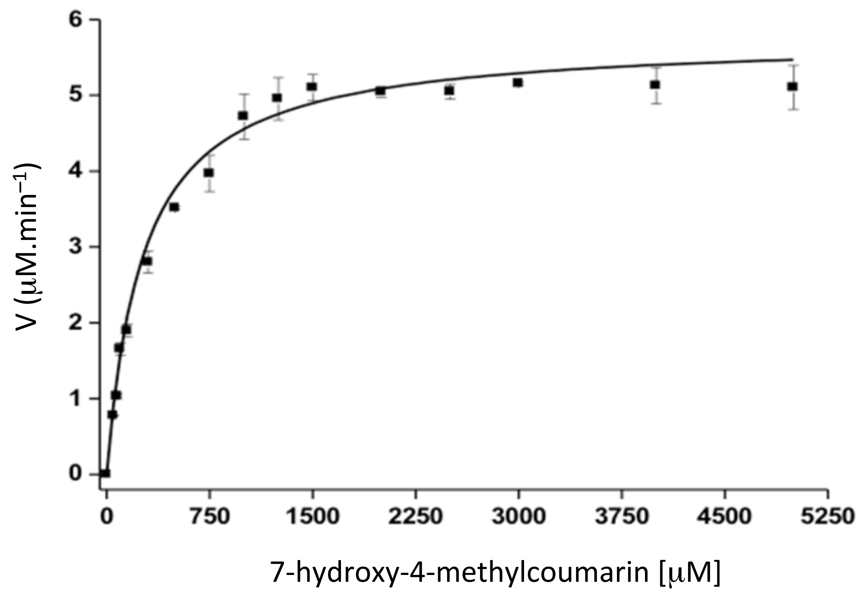

Figure S7. Michaelis-Menten graph of wild-type RadH chlorination of 7-hydroxy-4-methyl coumarin **11**.

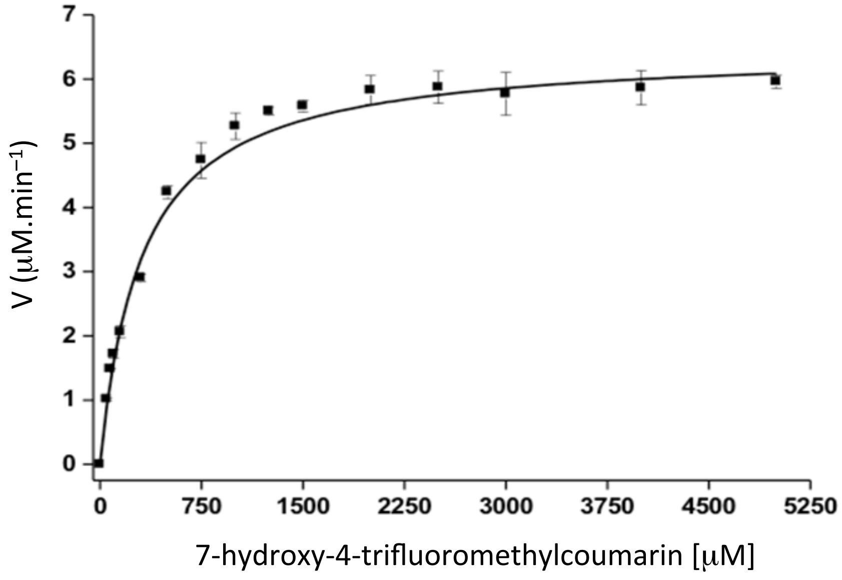

Figure S8. Michaelis-Menten graph of wild-type RadH chlorination 7-hydroxy-4-trifluoromethyl coumarin **10** .

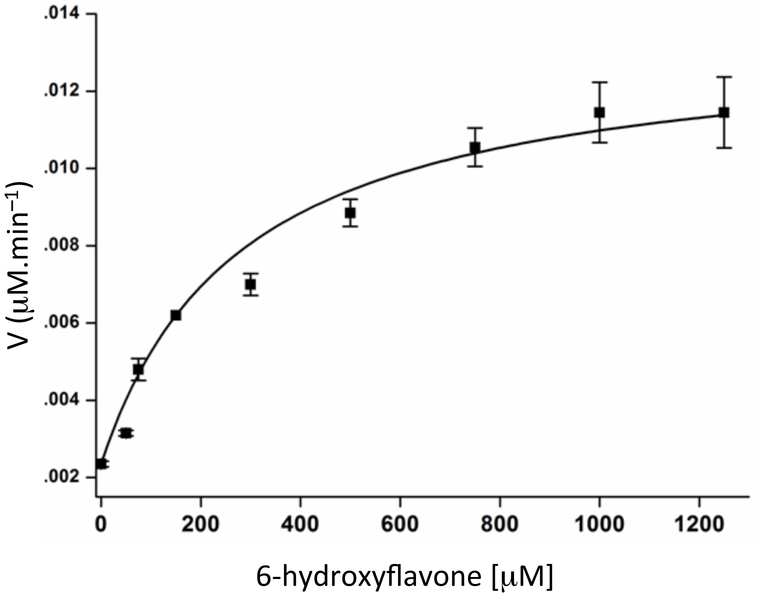

Figure S9. Michaelis-Menten graph of wild-type RadH reaction with 7-hydroxyflavone **12**.

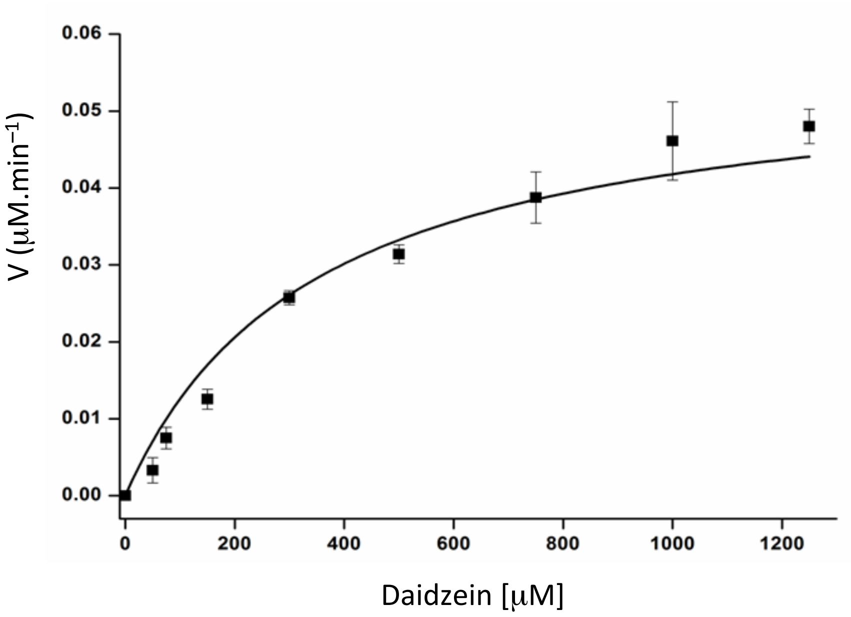

Figure S10. Michaelis-Menten graph of wild-type RadH reaction with daidzein **14**.

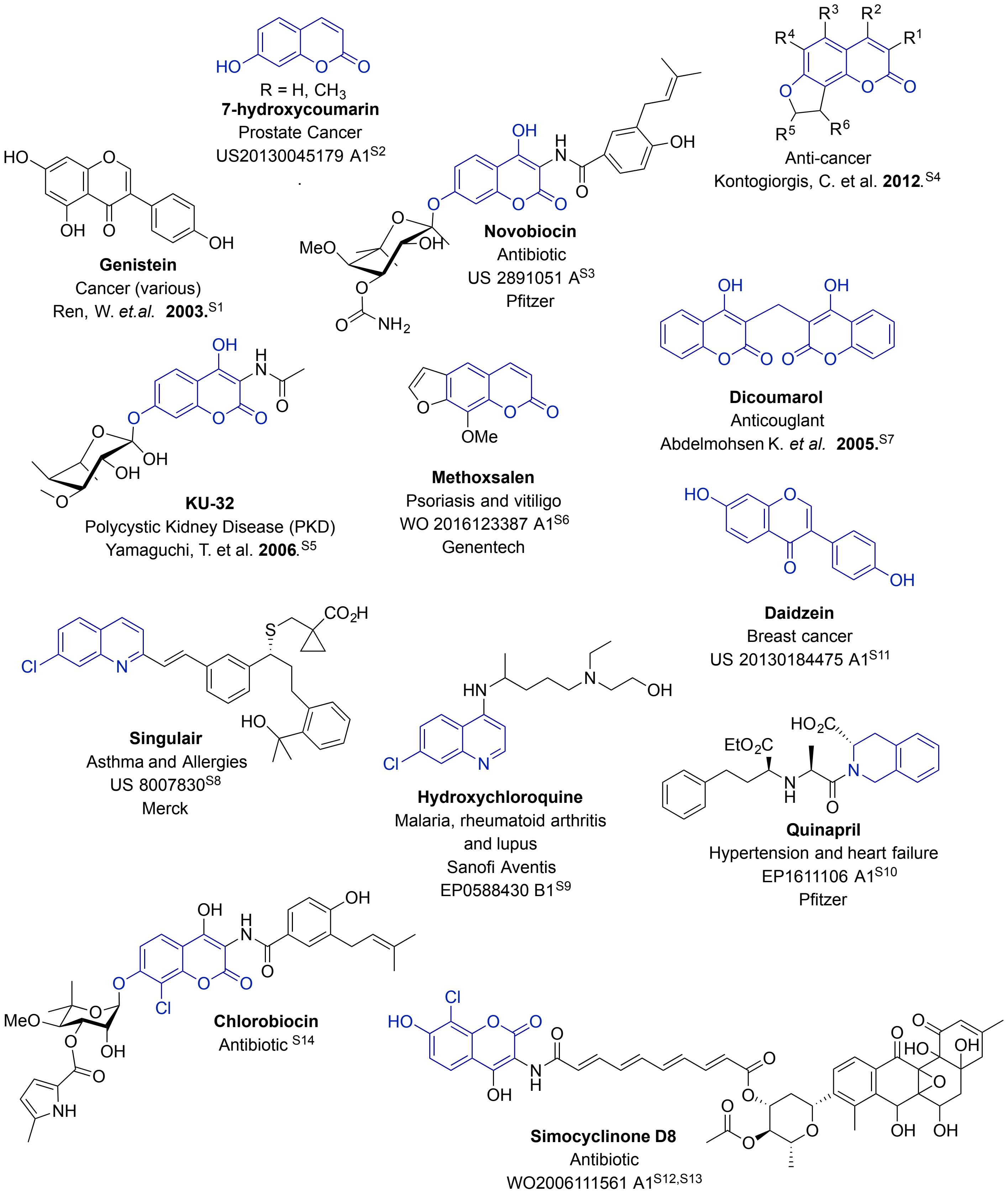

Figure S11. Some examples of pharmaceutically relevant compounds in the journal and patent literature derived from quinoline, coumarin and flavanoid scaffolds.

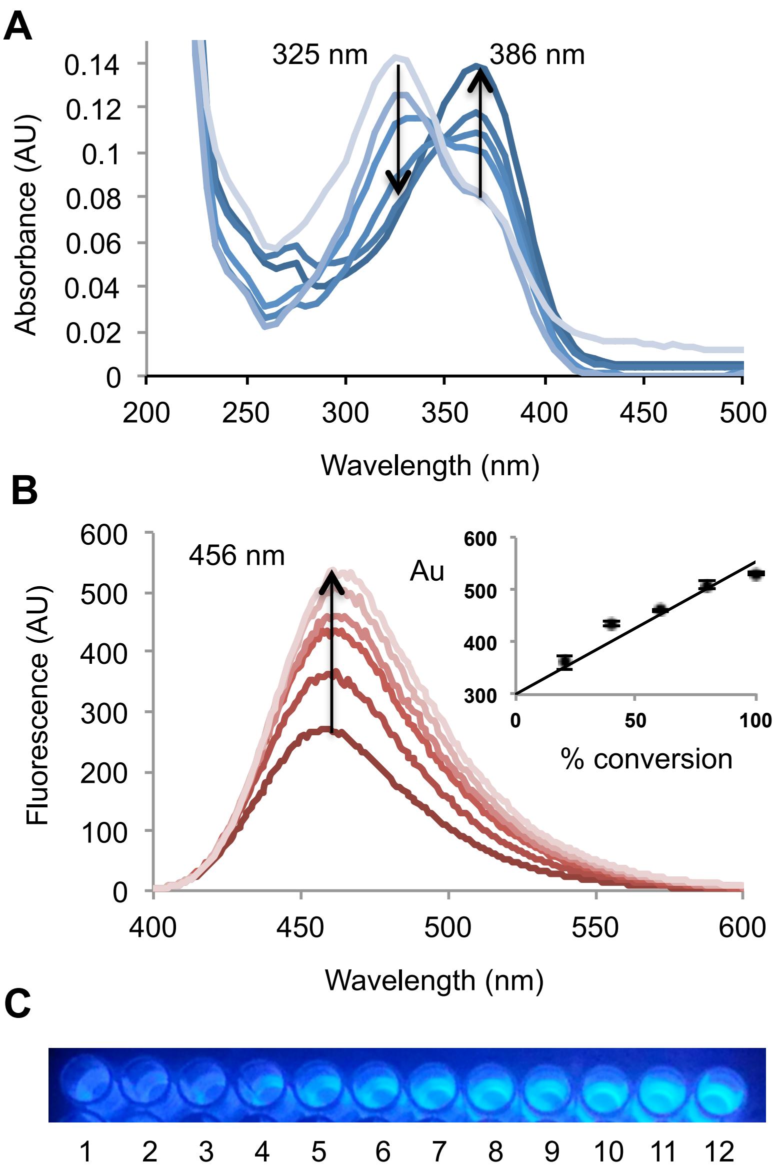

Figure S12. A fluorescence based screen for RadH activity. **(A)** Change in the UV spectra of 7-hydroxycoumarin (λ_{max} = 325 nm) upon chlorination to form 8-chloro-7-hydroxycoumarin (λ_{max} = 386 nm). **(B)** Fluorescence spectra of increasing concentrations of 8-chloro-7-hydroxycoumarin when excited at 386 nm. **(C)** RadH fluorescence plate reader assay (96 well format) showing increased production of 8-chloro-7-hydroxy-coumarin with time. Reaction conditions: RadH (15 µM), Fre (2.5 µM), FAD (1 µM), NADH (2.5 mM), $MgCl_2$ (5 mM) and 7-hydroxycoumarin (0.5 mM) in 10 mM potassium phosphate buffer, pH 7.4. After denaturing to remove proteins (90 °C for 5 minutes), the wells were loaded with supernatant from the reaction mixture at 10 min time intervals (well 1, t = 0 min to well 12 t = 120 min).

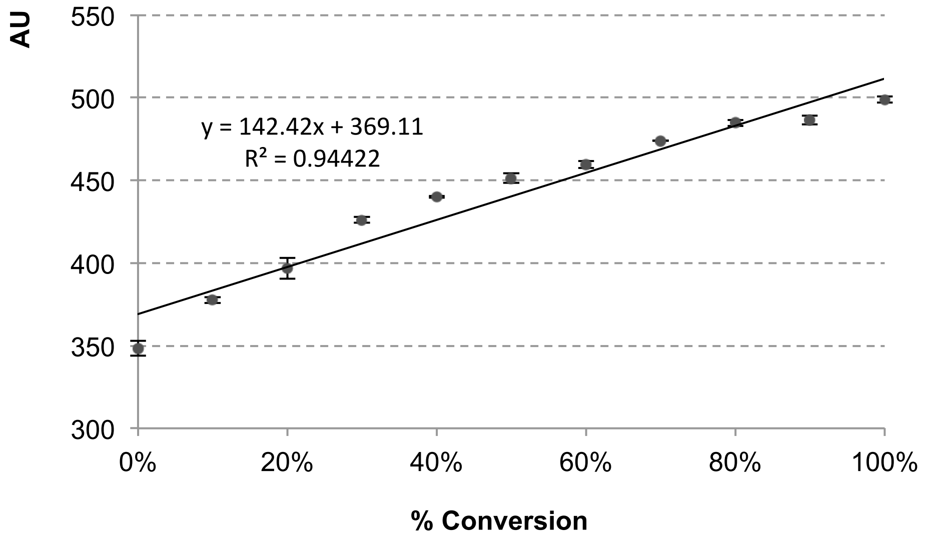

Figure S13. Fluorescence calibration curve showing a linear increase in fluorescence emission with exact molar ratios of **8**:**8a** increasing in 10% intervals. The % conversions are as follows: 0 % = 0.5 mM **8**; 10 % = 0.45:0.05 mM **8**:**8a**; and 20 % = 0.40:0.10 mM **8**:**8a** *etc*. The measurements were recorded in triplicate, the errors are low, and the R^2 value of 0.944 indicates that there is an excellent linear correlation between fluorescence and the molar ratio of **8**:**8a**. The fluorescence emission was measured using an Infinite® M1000 PRO plate reader (TECAN) by exciting the samples at 360 nm and reading the fluorescence emission above 520 nm.

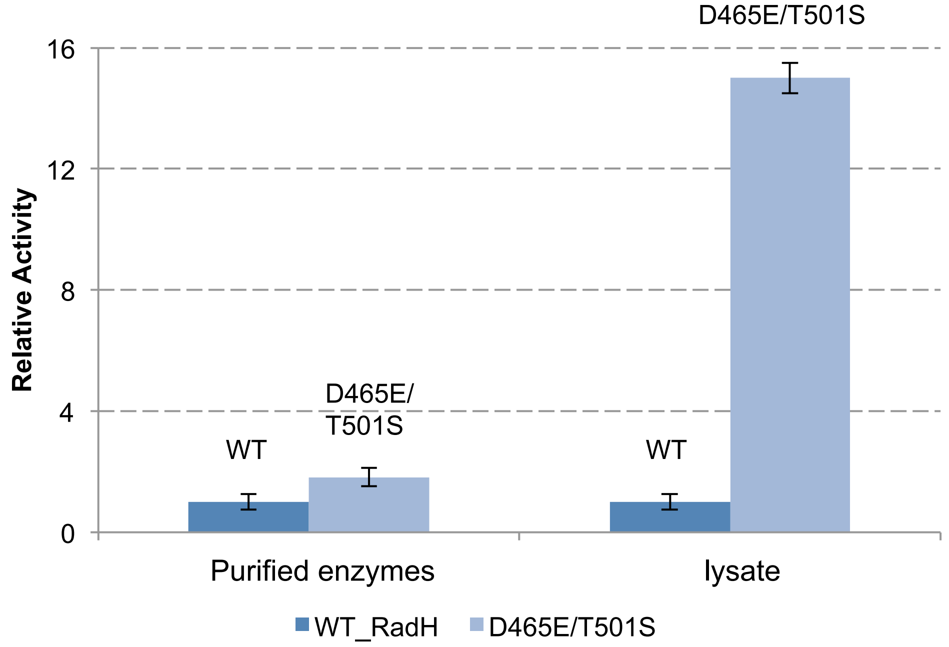

Figure S14. Relative activities wild-type RadH (dark blue, set to 1) and D465E/T501S mutant (pale blue) with 7-hydroxycoumarin **8** as a substrate in assays with pure enzymes (left) and lysate (Right). Assay conditions were: Fre (2.5 µM), FAD (1 µM), NADH (2.5 mM), $MgCl_2$ (10 mM) and substrate **8** (0.5 mM) in a total volume of 200 µL in 10 mM potassium phosphate buffer, pH 7.4. The reaction was measured using HPLC after 2 hour incubation at 30°C.

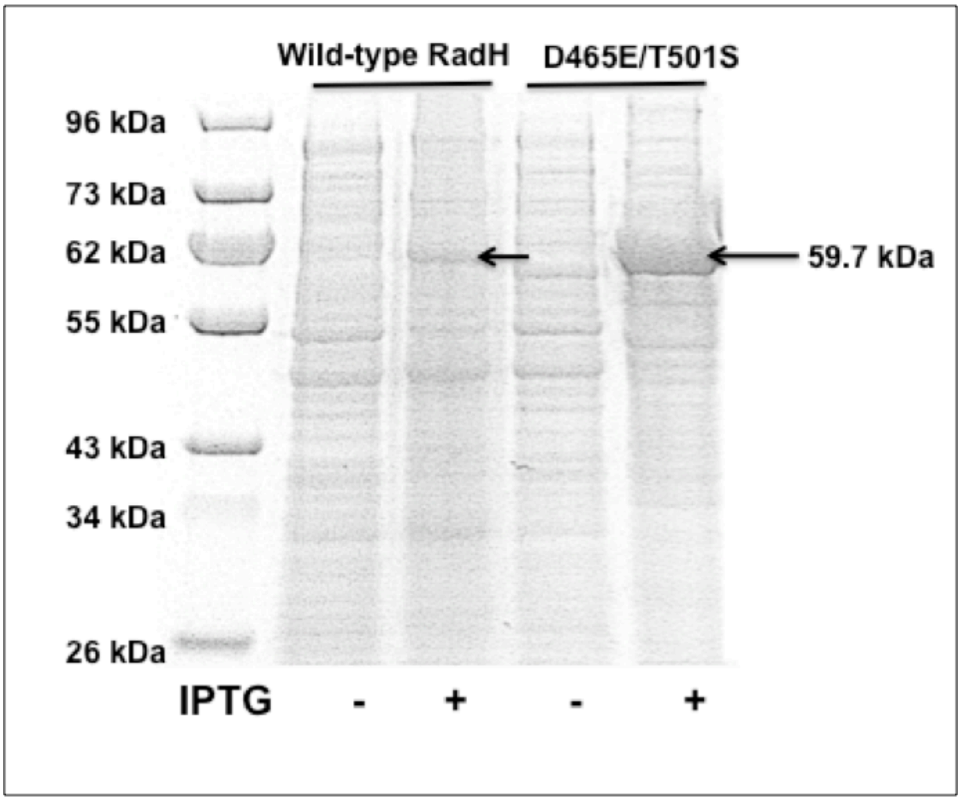

Figure S15. SDS-PAGE analysis of protein expression for the wild-type RadH and D465E/T501S variant enzymes. Reducing SDS-PAGE gel of cell lysates: lane 1, wild-type RadH uninduced cell lysate; lane 2, wild-type RadH 3 hours after IPTG induction; lane 3, D465E/T501S variant uninduced cell lysate; lane 4, D465E/T501S variant 3 hours after IPTG induction. Protein expression was induced with 0.1 mM IPTG (final concentration).

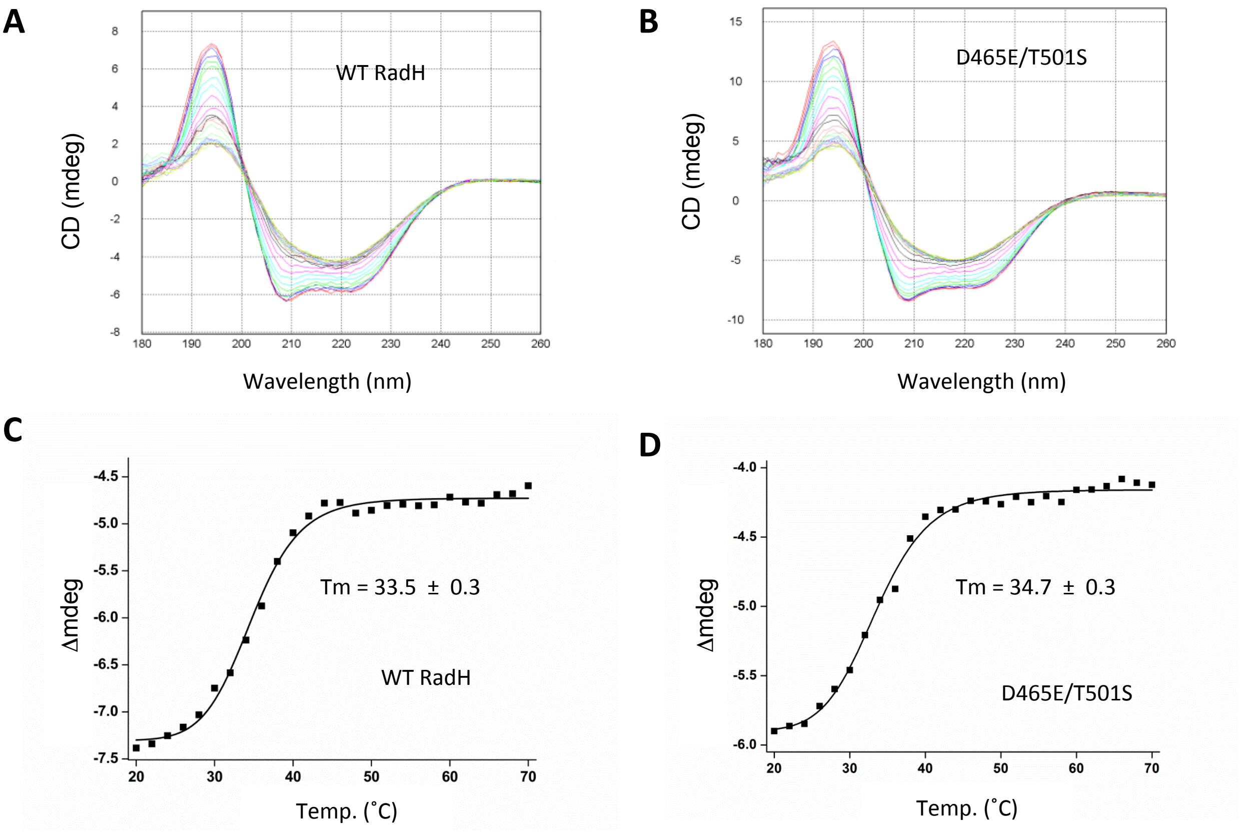

Figure S16. Thermal stability and melting temperatures for wild-type RadH and D465E/T501S variant enzymes. CD spectrum showing spectral changes during thermal stability assay for (**A**) wild-type RadH and (**B**) the D465E/T501S variant. Thermal stability/melting curve of (**C**) wild-type RadH, and (**D**) the D465E/T501S variant. The CD spectrum were measured every 2 °C, from 20 °C up to 80 °C with 10 μM enzyme in a 0.5 mm path length cuvette at fixed temperature for 2 minute with the temperature ramp of 0.2 °C per minute. The absorbance changes at 222 nm was plotted against temperature, and fitted with a nonlinear curve fitting function to find melting temperature of the proteins.

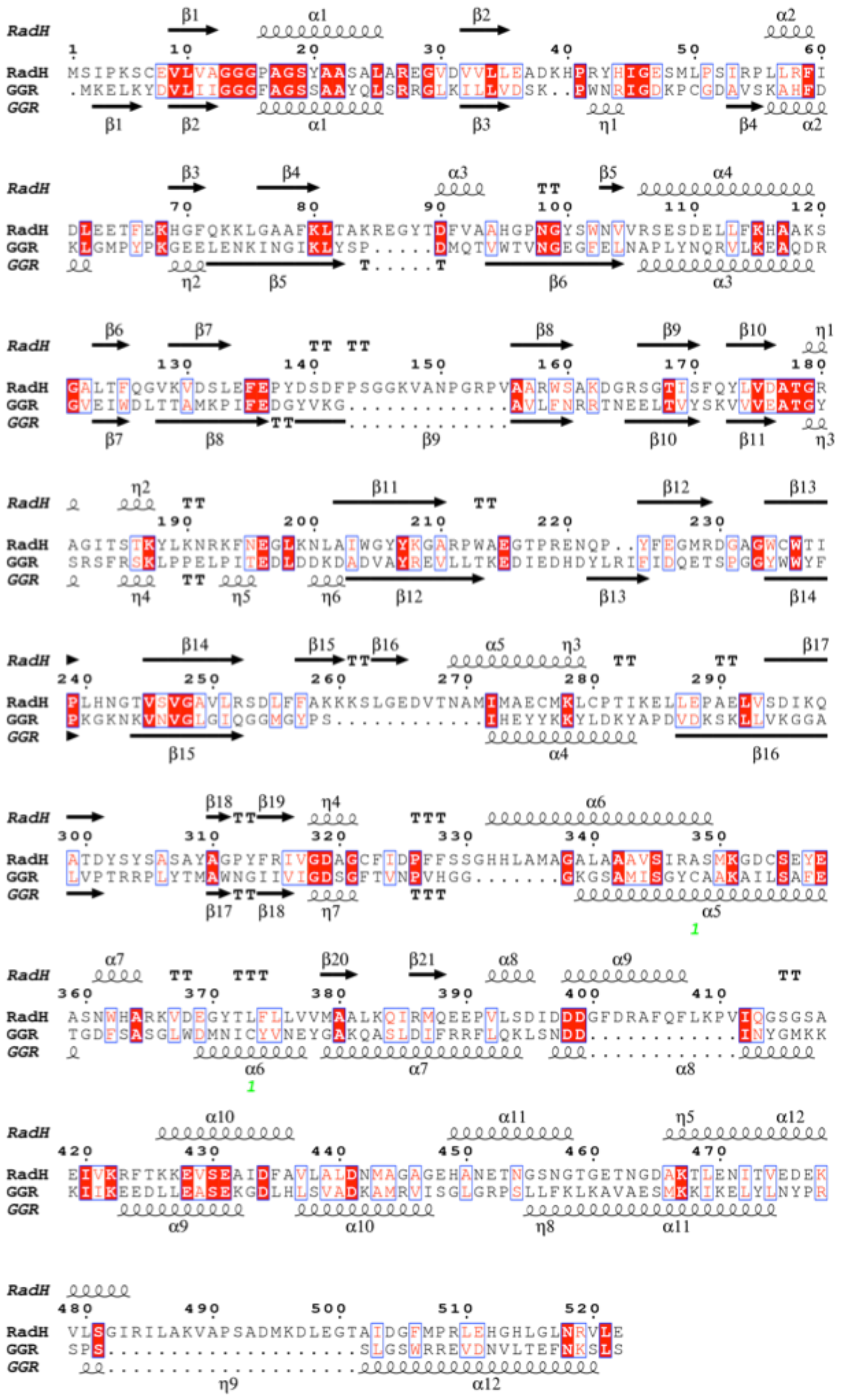

Figure S17. Sequence and 2D secondary structural alignment between Geranylgeranyl reductase (GGR) protein (Pdb id : 3ATQ) and RadH homology model. Helices and beta sheets are numbered and the conserved residues indicated by boxes. ESPript 3.0 structural alignment server was used to generate the figure. GGR is a flavoprotein from *Sulfolobus acidocaldariu* with 19.8 % identity and 33.9 % similarity to RadH. Whilst other proteins have higher sequence identity, GGR has the best overall sequence coverage and similarity.

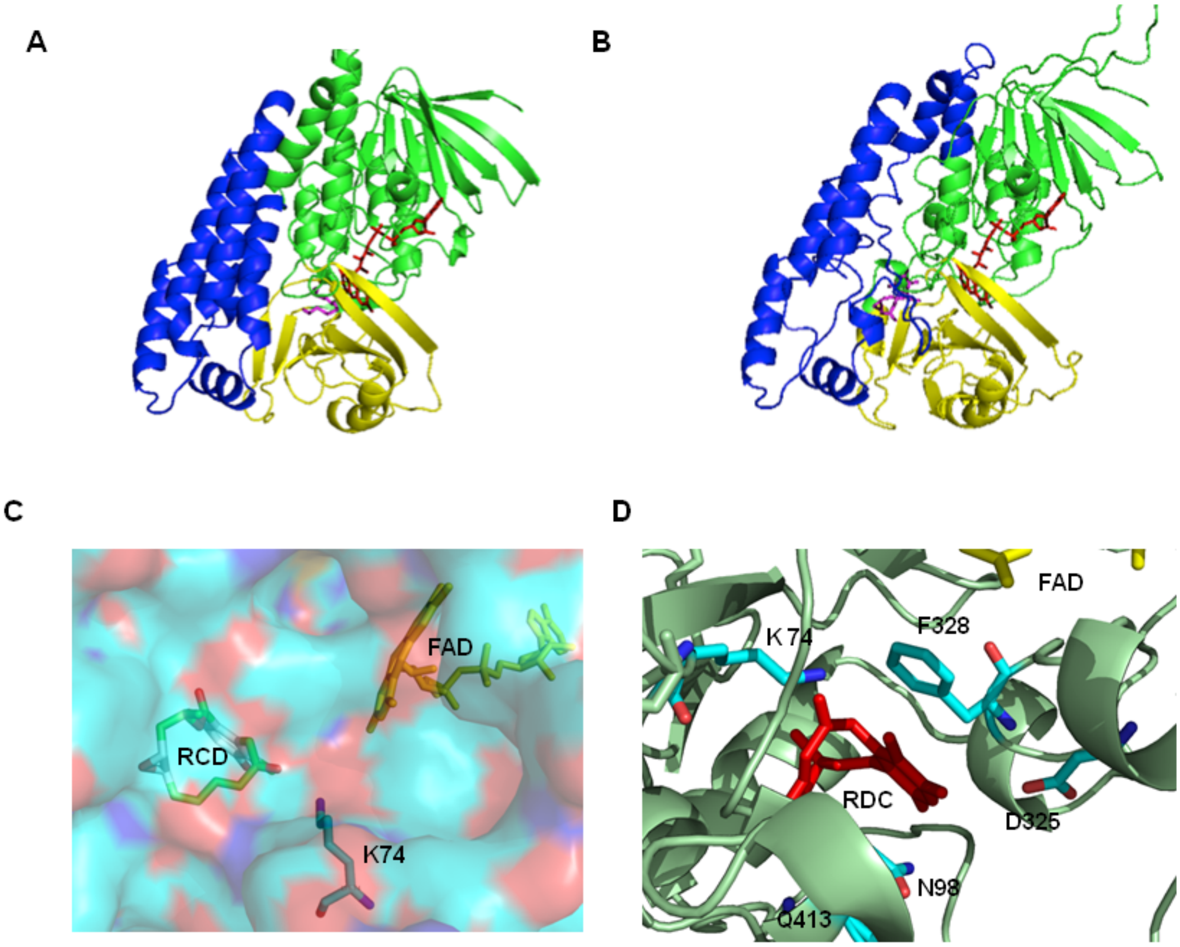

Figure S18 (**A**) Crystal structure of geranylgeranyl reductase (GGR) from *sulfolobus acidocaldariu* (Pdb id : 3ATQ). (**B**) Derived homology model of RadH based on the X-ray structure of GGR (PDB 3ATQ). In figure A and B, FAD is shown in red, while bound ligand (tetradecane or Radicicol) are shown in magenta. The protein back bone cartoon colour is based on different domains, colors for the secondary structure correspond to the domain colors, green, yellow and blue represent the FAD-binding, catalytic and C-terminal domains, respectively. (**C**) The spacious radicicol binding pocket in RadH enzyme displayed as surface model. (**D**) The relative position of active site Lys74, Asp325 and other active site residues in relation to radicicol (RDC) in the RadH structure is shown in a different orientation.

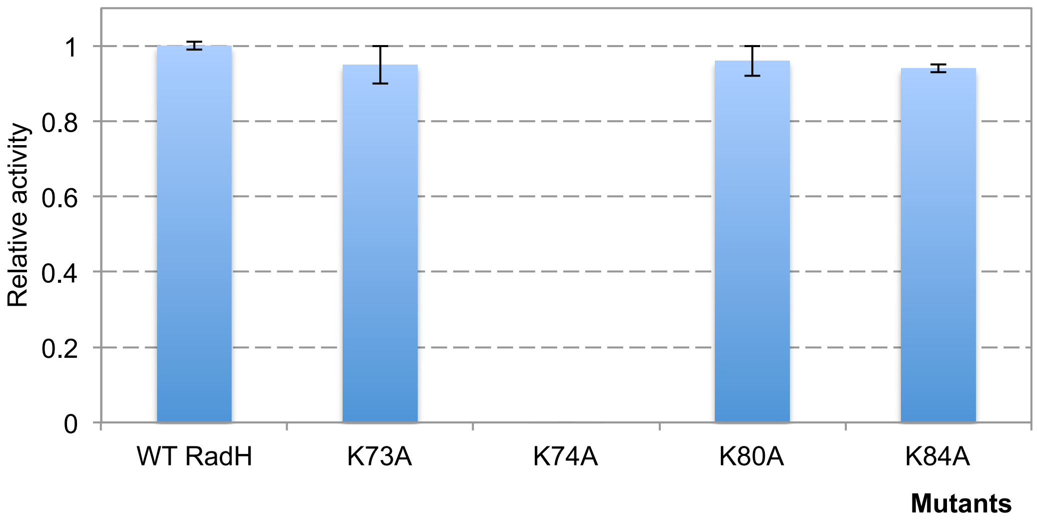

Figure S19 Relative activities of RadH active site lysine to alanine variants towards 6-hydroxyisoquinoline **6** are shown. Assay conditions were: RadH (15 µM), Fre (2.5 µM), FAD (1 µM), NADH (2.5 mM), $MgCl_2$ (10 mM) and substrate (0.5 mM) in a total volume of 200 µL in 10 mM potassium phosphate buffer, pH 7.4, with 2 hour incubation at 30°C.

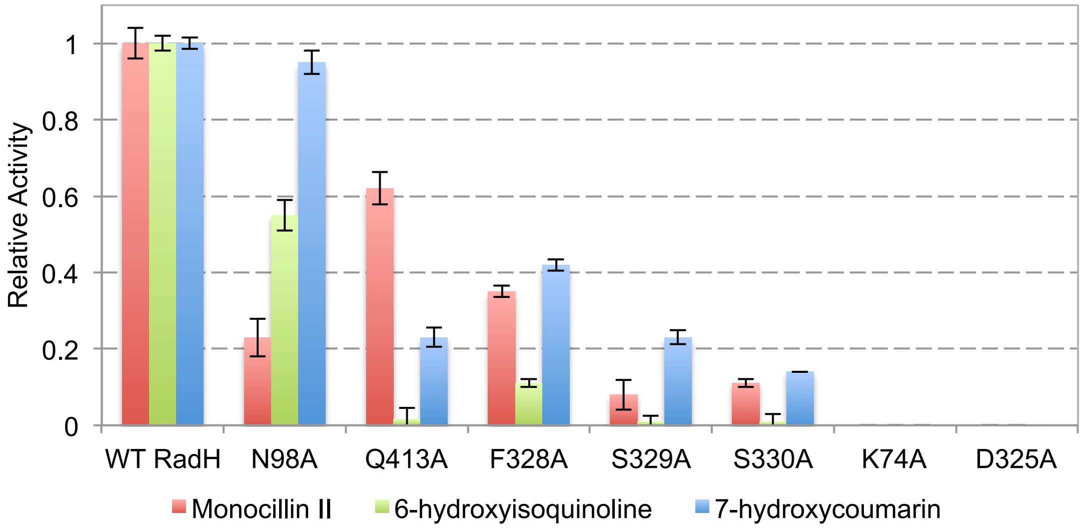

Figure S20 Relative activities of RadH active site variants towards monocillin II (red bar graphs), 6-hydroxisoquinoline (green bar graphs) and 7-hydroxycoumarin (blue bar graphs) are shown. Assay conditions were: RadH (15 µM), Fre (2.5 µM), FAD (1 µM), NADH (2.5 mM), $MgCl_2$ (10 mM) and substrate (0.5 mM) in a total volume of 200 µL in 10 mM potassium phosphate buffer, pH 7.4. The reaction was measured using HPLC after 18 hour incubation for monocillin II **1** and after 2 hour incubation for 6-hydroxyisoquinoline **6** and 7-hydroxycoumarin **8** at 30°C.

```
CLUSTAL O(1.2.4) multiple sequence alignment


RadH            MSIPKSCEVLVAGGGPAGSYAASALAREGVDVVLLEADKHPRYHIGESMLPSIRPLLRFI    60
Rdc2            MSVPKSCTILVAGGGPAGSYAAAALAREGNDVVLLEADQHPRYHIGESMLPSLRPLLRFI    60
Gsfl            MAIPQSCTVLVAGGGPGGSYTAAALAREGVDVVLLEADCHPRYHIGESLLPSMRYLLRFI    60
AcOYAhal        MAIPQKATVLVIGGGPGGSYSASALAREGIDTVVLEADVFPRYHIGESLVASIRPFLKFI    60
AclH            MSIPNRCTVLVVGGGPAGSYAAAALAREGVDTVLLEADVFPRYHIGESMLPSIRHFLRFI    60
GedL            ------------------------------------------------MLASMRFFLRFI    12
                                                                :: *:* :*:**
                           K74                     N98
RadH            DLEETFEKHGFQKKLGAAFKLTAKREGYTDFVAAHGPNGYSWNVVRSESDELLFKHAAKS    120
Rdc2            DLEDKFDAHGFQKKLGAAFKLTSKREGYTDFIAAHGPRGYSWNVVRSESDEILFNHARES    120
Gsfl            DLEDTFEQHGFQKKLGAFFKLNAKSAGYTDFIRANGPNGYSWNVVRSESDEILFRHATKS    120
AcOYAhal        DLDDTFVNYGFVRKNGAAFKLNNQKEAYTDFILEAGADTFAWNVVRSESDDLMFKHAAKS    120
AclH            DLDSKFDSYGFVNKNGAAFKLNSKPEAYTDFIAAGGPGSHAWNVVRSEADHLMFKHAGEN    120
GedL            DLEEQFDAYGFQKKYGATFKINSKREAYTDFSASLGPGGYAWNVIRSEADDLIFRYAGEQ    72
                **:. *  :** .* ** **:. :  .****    *   .:***:***:*.::*.:* :.

RadH            GALTFQGVKVDSLEFEPYDSD----FPSGGKVANPGRPVAARWSAK-DGRSGTISFQYLV    175
Rdc2            GAQAFQGIKINAIEFEPYEEE----YPNGEKVANPGKPTSAKWSSK-DGSSGDIAFKYLV    175
Gsfl            GAKTFENVSLKSVNFEPYEND---KFTSQDKLTNPGRPVSAEWKTK-DGCSGTISFDYLV    176
AcOYAhal        GAQTFDGVRVTSIEFTDDDDD---------DDNNTNRPVSASWKAK-DGRTGSIEFDYLV    170
AclH            GAQVFDGVKVNSIEFEQIDGL---T--VDPSLSELGRPVSATWSCKATGGKGSITFEYLI    175
GedL            GAHIFDGTKVDDIEFLSYDGADGANFTPAAFLVNPGRPVAATWSRK-DGTRGRIKFDYLI    131
                **  *:.  :  ::*   :                : .:*.:* *. *  *  * * *.**:

RadH            DATGRAGITSTKYLKNRKFNEGLKNLAIWGYYKGARPWAEGTPRENQPYFEGMRDGAGWC    235
Rdc2            DATGRIGIMSTKYLKNRHYNEGLKNLAIWGYYKNNIPWAQGTPRENQPFFEGMRDGAGWC    235
Gsfl            DATGRVGILSTKYLKNRKFNESFRNIAMWGYFKGNIPPSPGTDRENQPISEGMRDGSGWV    236
AcOYAhal        DASGRAGITSTKYLKNRTFNNYLKNVASWGYWEGATPYGMGTPVEGQPFFEALQDGSGWV    230
AclH            DATGRAGLVSTKYMKNRRYNQGLKNVASWGYWSNAGSYGVGTPREGDPYFEAIEDGSGWV    235
GedL            DASGRAGIISTKYLKNRTVNEGLRNIANWSYWKGAKVYGEGSDQQGSPFFEALTDGSGWC    191
                **:** *: ****:***  *: ::*:* *.*:..    . *:  :..*  *.: **:**

RadH            WTIPLHNGTVSVGAVLRSDLFFAKKKSLGEDVTNAMIMAECMKLCPTIKELLEP--AELV    293
Rdc2            WTIPLHNGTVSVGAVMRKDLFFEKKKSLGENATNTQIMAECMKLCPTIGELLAP--AELV    293
Gsfl            WMLPLHNGTVSIGAVVRKDIFQAKKKALPEGTTEAQTLASLVALCPTISSYLEP--AELA    294
AcOYAhal        WFIPLHNNTTSIGIVMNQELSTQKKKLST-TTSSRAFYLESLAGARGISRLLDPTTATLT    289
AclH            WLIPLHNGTTSIGVVMNQEAATAKKRET--GATTKDLYLNTIKNTPGVWQLLDK--AELQ    291
GedL            WAIPLHNGTLSVGVVMRQDLFFGKKKAAG-SPGSLEMYKLCLQSVPGISGLLED--AEIV    248
                * :****.* *:* *:...:    **:                :     :   *    * :
                                               D325
RadH            SDIKQATDYSYSASAYAGPYFRIVGDAGCFIDPFFSSGHHLAMAGALAAAVSIRASMKGD    353
Rdc2            SDIKQATDYSYSATAYAGPNFRIVGDAGCFIDPFFSSGHHLAVAGALAAAVSINASIKGD    353
Gsfl            SGIRQAADYSYSANAYAGPNFRIVGDAGCFIDPFFSSGHHLALSSALAAATSINACIRGD    354
AcOYAhal        SDIKHASDWSYNASAYGSPYLRIVGDAGAFIDPYFSSGVHLAVSGGLSAAVSIAASIRGD    349
AclH            SDLKSASDWSYNASSYASPYLRIVGDAGCFIDPFFSSGVHLALASGLSAALTIRAAQRGD    351
GedL            SDVKMASDWSYSASAYAGPHFRVAGDAGCFIDPYFSSGVHLALVGGLSAATTIQAVRRGE    308
                *.:: *:*:**.*.:*..* :*:.****.****:**** ***: ..*:** :* *  :*:

RadH            CSEYEASNWHARKVDEGYTLFLLVVMAALKQIRMQEEPVLSDIDDDGFDRAFQFLKPVIQ    413
Rdc2            CTEYEASRWHAKKVDEGYTLFLLVVMAALKQIRMQENPVLSDVDEDGFDRAFQFLRPVIQ    413
Gsfl            CNEFDASRWFAKKVDEGYTLFLVVVMAALKQIRMQEQPILSDLDEEGFDRAFTILRPVIQ    414
AcOYAhal        CPEEAAWKWHSQGVANRYGRFLLVVLGATKQIRAGDRPVLNGVGDEGFDEAFMVIRPGEF    409
AclH            CDEQAAVSWHSKKVAEGYTRFLLVVMSALKQISDREKPVLTDFDEDSFNRAFDFFRPIIQ    411
GedL            TSEFSAAKWHSSKVTEGYTRFLLVVMAVLRQLRKQNAAVITDDKEEGFDTAFGLIQPVIQ    368
                  *  *  *.:  * : *  **:**:.. :*:   :  :::..  ::.*: **  .::*

RadH            GSGSAEIVK-RFTKKEVSEAIDFAVLALDNMAGAGEH------------ANETNGSNGT    459
Rdc2            GSGSSEAVK-KFTKEDVAQTIDFAVHALNNMAELDMDIPEHMINGDKEGENGVTNGNNGA    472
Gsfl            GAADKETAP-KAKGESITETIDLCLTALNDLHDTELQRKLTSIVE----------AKG-    461
AcOYAhal        PFFFFSFSFFFLSSEGVGVVVWNCAN---------------------------------    435
AclH            GTADVDKN---LTQAEIAQTIEFCVQAFQTASTDEQDAVMTKVAA-----INSQNGTEGA    463
GedL            GQADTGESEQ----QRMVAGVQFSLERFGQATPEAQRALLDKVQF--------------    409
                                     :   :  .
                   D465                                        T501
RadH            GETNGDAKTLENITVEDEKVLSGIRIL--AKVAPSADMKDLEGTAIDGFMPRLEHGHLGL    517
Rdc2            AKTAGLASNMEKLTNDEEKVLNGLRIL--GKAAPGGTLADFEGTAIDGLMPRLEHGKLGL    530
Gsfl            --TPEQEQLLGKLSPDETAALHRMRAM--HSILPMGELEDFENSNIDGFKARLEKGSLGL    517
AcOYAhal        ------------------------------------------------------------    435
AclH            -----LRELHASLSADERRTLTTIQARQIIRSEDQMNIDNFTIDVIDGMVPRLERSSLGL    518
GedL            --AGQNAEELEKLTADELAVLHNIIGRQLKMTKVEKNLDNFTRDVIDGWAPRVERGKLGL    467


RadH            NRV---------------------------------------- 520
Rdc2            NKV---------------------------------------- 533
Gsfl            RRERALCRDHAGD----------LQM----------------- 533
AcOYAhal        ------------------------------------------- 435
AclH            ARFVPKAQTSRED----------GLRATLGLPEKQKSIFSY 549
GedL            QRADTSIMTAEMKDLFQLNRSLDSTKAGIQLPA-------- 500
```

Figure S21 Multiple sequence alignment (key residues shown in black boxes) for fungal Fl-Hal:

RadH, *Chaetomium chiversii*
Rdc2, *Pochonia chlamydosporia*
Gsfl, *Penicillium aethiopicum*
AcOYAhal, *Aspergillus carbonarius*
AclH, *Aspergillus oryzae*
GedL, *Aspergillus terreus*

A

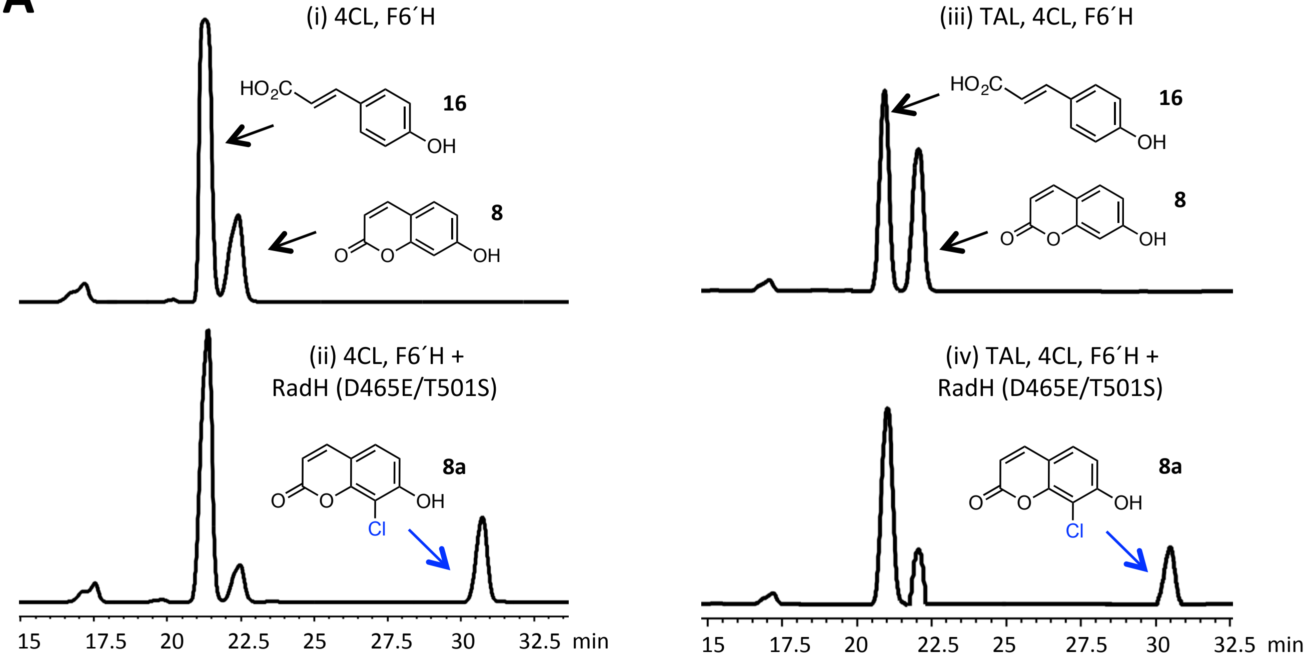

B

E. coli strain	Feeding	7-hydroxycoumarin **8** (mg/L)	8-chloro-7-hydroxy-coumarin **8a** (mg/L)
(i) 4Cl, F6´H	p-coumaric acid (0.5 mM)	7.8 ± 0.4	
(ii) 4Cl, F6´H + RadH (D465E/T501S)	p-coumaric acid (0.5 mM)	6.5 ± 0.6	0.8 ± 0.2
(iii) TAL , 4Cl, F6´H	none	2.5 ± 0.1	
	L- Tyrosine (1.0 mM)	5.1 ± 0.3	
(iv) TAL, 4Cl, F6´H + RadH (D465E/T501S)	none	1.4 ± 0.1	1.1 ± 0.1
	L- Tyrosine (1.0 mM)	2.5 ± 0.3	1.9 ± 0.3

Figure S22. (A) HPLC traces showing *p*-coumaric acid (**16**), 7-hydroxycoumarin (**8**) and 8-chloro-7-hydroxycoumarin (**8a**) after fermentation of (i) *E. coli* possesing CL and F´6H with feeding of **16**; (ii) *E. coli* possesing CL, F´6H and RadH (D465E/T501S) with feeding of **16**; (iii) *E. coli* possesing TAL, CL and F6´H; (iv) *E. coli* possesing TAL, CL, F6´H and RadH (D465E/T501S). **(B)** Titres of 7-hydroxycoumarin (**8**) and 8-chloro-7-hydroxycoumarin (**8a**) produced by *E. coli* strains (i-iv).

SUPPLEMENTARY METHODS

1.1 General. All chemical reagents and solvents were purchased from Sigma-Aldrich Company Ltd, Fisher Scientific UK Ltd, or Alfa Aesar and were used without further purification.

1.2 Cloning, expression, and purification of enzymes. The gene for RadH from *Chaetomium chiversii* was synthesized (GenScript, Piscataway, USA) and cloned into the pET 28b expression vector (Invitrogen) using restriction sites Nde1 and Xho1. The RadH-containing vector (pET28b) was used to transform *E. coli* Rosetta 2 (DE3) cells (Novagen) for the overexpression of the RadH halogenase. LB medium containing kanamycin (50 µg/mL) and chloramphenicol (35 µg/mL) was inoculated with the transformed cells and incubated at 37 °C overnight. The cells were diluted 1:100 in a 2xYT medium broth (Formedium) and incubated shaking at 30 °C, 180 rpm until an optical density (OD_{600nm}) was 0.5. The temperature was dropped to 24 °C until the OD reached 0.6 and the protein expression was induced with addition of IPTG (0.1 mM), before growing overnight at 20 °C, followed by harvesting of cells (4 °C, 30 min, 4000 x *g*).
Cell pellets, were resuspended in 25 mL lysis buffer (50 mM phosphate, 500 mM NaCl, and 10 mM imidazole, pH 7.4), protease inhibitor tablets (Roche) were added and the cells disrupted by sonication. The lysate was centrifuged (4 °C, 30 min, 10,000 x *g*) and the soluble cell extract loaded onto a Ni-NTA column. The bound protein was then washed with 50 mM phosphate buffer (pH 7.4) containing 60 mM imidazole and 500 mM NaCl. Pure RadH was eluted in phosphate buffer containing 300 mM imidazole, protein samples were subjected to buffer exchange with 100 mM phosphate buffer containing 10% glycerol using a Vivaspin 20 centricon (30,000 MWCO) before storing at -20 °C. The flavin reductase enzyme (Fre) from *E. Coli* was purified as described previously.[S15]

1.3 Mutagenesis, error prone PCR and screening of mutant library. Site-directed mutagenesis of the *RadH* gene was performed on the pET28b–RadH plasmid (described above in section 1.2) using the QuikChange kit (Stratagene) to mutate residues. The primers that were used (MWG Eurofins, Germany) are shown below:
K73A_forward primer 5'-GAA AAA CAT GGC TTC CAG GCA AAA CTG GGT GCG GCC -3';
K73A_reverse primer 5'- GGC CGC ACC CAG TTT TGC CTG GAA GCC A TG TTT TTC-3';
K74A _ forward primer 5'-AAA CAT GGC TTC CAG AAA GCA CTG GGT GCG GCC TTT-3';
K74A _ reverse primer 5'- AAA GGC CGC ACC CAG TGC TTT CTG GAA GCC ATG TTT-3';
K80A_ forward primer 5'-CTG GGT GCG GCC TTT GCA CTG ACC GCC AAA C-3'; K80A_

reverse primer 5'-GTT TGG CGG TCA G TGC AAA GGC CGC ACC CAG-3'; K84_ forward primer 5'-A TTT AAA CTG ACC GCC GCA CGT GAA GGTTATACG-3'; K84_ reverse primer 5'-CGT ATA ACC TTC ACG TGC GGC GGT CAG TTT AAA T-3'; N98A_forward primer 5'-CT CAT GGC CCG GCT GGT TAC TCT-3'; N98A_reverse primer 5'-CA AGA GTA ACC AGC CGG GCC ATG-3'; D325A_forward primer 5'-GGT TGC TTC ATC GCT CCG TTT TTC A-3'; D325A_reverse primer 5'-GCT GAA AAA CGG AGC GAT GAA GCA-3'; F328A_forward primer 5'- ATC GAT CCG TTT GCC AGC TCT GGC-3'; F328A_reverse primer 5'-TG ATG GCC AGA GCT GGC AAA CGG AT-3'; S329A_forward primer 5'- GAT CCG TTT TTC GCC TCT GGC CAT CA-3'; S329A_reverse primer 5'-GTG ATG GCC AGA GGC GAA AAA CGG -3'; S330A_forward primer 5'- CCG TTT TTC AGC GCT GGC CAT CAC-3'; S330A_reverse primer 5'-CAG GTG ATG GCC AGC GCT GAA AAA-3'; Q413A_forward primer 5'- AAA CCG GTC ATT GCA GGC TCT GGT-3'; Q413A_reverse primer 5'-CT ACC AGA GCC TGC AAT GAC CGG-3'.

The GeneMorph II EZClone domain mutagenesis kit (Agilent Technologies) was used to create a random mutagenesis library of RadH using the error prone PCR method. The following primers were used for the PCR reaction and the standard experimental conditions described in the kit were used. RadH_forward primer 5'-ATG AGT ATT CCG AAA TCG TGT GAA-3'; RadH_reverse primer 5'-CTC GAG CAC GCG ATT CAG ACC CAG-3'. The mutations were confirmed by complete plasmid DNA sequencing (MWG Eurofins, London, UK). The individual colonies from the agar plate containing transformed BL21(DE3) pLysS cells with RadH mutants were grown in 96 deep well blocks in auto induction super broth media (Formedium) for 3 days at 25 °C, 250 rpm. The cells were then centrifuged (4 °C, 20 min, 4,000 x *g*) and the supernatant were removed and the centrifuged cells were then lysed by multiple freeze and thaw cycles. The whole cell crude assay was performed with the lysed cells at 30 °C with shaking at 800 rpm for 3 h with Fre (1 µM), FAD (1 µM), NADH (0.5 mM), $MgCl_2$ (10 mM) and 7-hydroxycoumarin (0.5 mM) to a total volume of 300 µL in 10 mM potassium phosphate buffer, pH 7.4. Reactions were monitored for production of 6-chloro-7-hydroxycoumarin using an Infinite® M1000 PRO plate reader (TECAN) exciting the samples at 360 nm and reading the fluorescence emission above 520 nm. The 24 mutants showing highest fluorescence emission were further assayed using UPLC to quantify product formation and by repeating the assay with purified enzymes of known concentration using the protocols described below.

1.4 RadH biotransformations, kinetics and regioselectivity. Purified RadH (15 µM) was incubated at 30 °C with shaking at 800 rpm for 2 h with Fre (2.5 µM), FAD (1 µM), NADH (2.5 mM), $MgCl_2$ (10 mM) and substrate (0.5 mM) to a total volume of 200 µL in 10 mM potassium

phosphate buffer, pH 7.4. The reactions were quenched by heat shock at 95 °C for 5 min or with an equal amount of methanol and precipitated protein was removed by centrifugation, before analysis *via* HPLC on an Agilent Technologies 1260 system using an Agilent Zorbax Eclipse Plus C18 4.6 x 100 mm x 3.5 μm column. The absorbance was measured at 325 nm with a 5 min gradient 5-75% H_2O/acetonitrile + 0.1% formic acid. The kinetic assays for the RadH reaction were performed by using RadH at various concentrations (from 5.0-12.5 μM) based on the observed substrate conversion. The assay reaction mixture was prepared by adding Fre (2.5 μM), substrate (50 μM - 5 mM) in ethanol stock solution (< 2.5% ethanol in final reaction mixture), $MgCl_2$ (10 mM) and FAD (10 μM) in a 20 mM potassium phosphate buffer, pH 7.4. The reaction was initiated by adding 2.5 mM NADH. The amount of product formed at 5, 7.5 and 10 minutes were measured by using a HPLC system (Agilent Technologies 1260 HPLC system) equipped with an Agilent Zorbax Eclipse Plus C18 4.6 x 100 mm x 3.5 μm HPLC column after quenching the reaction using ethanol. The absorbance was measured at 325 nm with a 5 min gradient 5 - 75% H_2O/acetonitrile + 0.1% formic acid. The product formation rate was plotted against the substrate concentration to obtain a Michaelis Menten type curve in the Origin Pro software and is fitted with the nonlinear curve fitting equation to find the kinetic parameters.

1.5 Homology modelling and substrate docking of RadH. The SWISS-MODEL homology server identified geranylgeranyl reductase (ggr) from *sulfolobus acidocaldariu* (pdb id 3ATQ) as one of the best templates for the homology model building of RadH from *Chaetomium chiversii,* based on the sequence identity and coverage between both proteins. Subsequently, the SWISS-MODEL homology server[S16,S17] was used to develop the homology model of RadH. AutoDock Vina[S18] was then used to dock the substrate radicicol in the active site RadH homology model as per the standard docking procedure. The binding of ligand in the active site of ggr and the position of active site Lys (K74) were used to guide and define the docking position of radicicol in the active site of the RadH model.

1.6 Plasmids construction for the 7-hydroxycoumarin and 8-chloro-7-hydroxycoumarin pathway. A synthetic gene (Genewiz, UK), codon optimised for *E.coli* expression, encoding tyrosine ammonia lyase (TAL), from *Saccharothrix espanaensis* (WP_015103237.1), was cloned into first cloning site of pACYCDuet-1 expression vector (Novagen) using restriction sites NcoI and HindIII to create pACYC_TAL plasmid. The gene for 4-coumaryl-CoA ligase (4CL) from *Streptomyces coelicolor* (WP_011029620.1) was amplified from a pQLinkN vector (provided by Prof. Jean-Loup Faulon, The University of Manchester) and cloned into the first cloning site of pRSFDuet-1 expression vector (Novagen) using restriction sites NcoI and Hind III. The gene for

feruloyl CoA 6′ hydroxylase (IbF6′H) from *Ipomoea batatas* (BAL22348.1) was synthesized (by GeneArt, Thermo Fischer Scientific, USA) with an N-terminal Glutathione-S-transferase (GST) fusion sequence (AAM73721.1). This entire gene was codon optimised for *E. coli* expression and was then cloned into the second cloning site of pRSFDuet-1 expression vector (with the 4Cl gene in the first cloning site as described above) using restriction sites NdeI and XhoI restriction enzymes to create pRSF_4CL_IbF6′H plasmid. RadH from *Chaetomium chiversii* was synthesized (GenScript, Piscataway, USA) and cloned into pCDFDuet-1 vector using restriction sites NcoI and HindIII to prepare pCDFDuet_RadH plasmid. The primers used for gene cloning are as follows:
Se_TAL_ forward primer AGG AGA TAT ACC ATG GAG ATG ACG CAG GTC GTG GAA CGC ; Se_TAL_ reverse primer ATG CGG CCG CAA GCT TTT AAC CGA AAT CCT TCC CAT C ; IbF6H_ forward primer AAG GAG ATA TAC ATA TGA TGT CCC CTA TAC TAG GTT A ; IbF6H_ reverse primer CTT TAC CAG ACT CGA GTT ATT CGA TGC GCG CAA ACG C ; St_4CL_ forward primer AGG AGA TAT ACC ATG GGG ATG GGA TCC ATG TTC CGC AGC GAG TAC ; St_4Cl_ reverse primer ATG CGG CCG CAA GCT TTC AGT GGT GGT GGT GGT GGT GTC GCG GCT CCC TGA GCT GTC GG
pCDFDuet_RadH(D465E/T501S) double mutant plasmid was created using the QuikChange kit (Stratagene) for site-directed mutagenesis along with the following primers (section 1.3): D465E_forward primer 5’- GT GAA ACG AAT GGT GAA GCA AAA ACC CT-3’; D465E_reverse primer 5’-AGG GT TTT TGC TTC ACC ATT CGT TTC ACC-3’; T501S_forward primer 5’- AA GAT CTG GAA GGC AGC GCG ATC GAT GG-3’; T501S_reverse primer 5’-CC ATC GAT CGC GCT GCC TTC CAG ATC TT-3’.

1.7 *E. coli* production and analysis of 7-hydroxycoumarin and 8-Chloro-7-hydroxycoumarin.
7-hydroxycoumarin producer strains were generated by transforming *E. coli* BL21 (DE3) cells with the pRSF_4CL_IbF6′H plasmid (requires feeding p-coumaric acid) or by co-transforming pRSF_4CL_IbF6′H and pACYC_TAL plasmids. The resulting *E. coli* strains were each transformed with pCDFDuet_RadH_D465E/T501S double mutant plasmid to produce 8-chloro-7-hydroxycoumarin producing strain. The *E. coli* transformants grown on LB agar plates that contained the selection antibiotics were picked and grown overnight in LB medium. This medium was then diluted to an optical density (OD_{600}) of 0.01 in a fresh 50 ml LB media (by 1/1000 dilution) supplemented with required antibiotics in a 250 ml Erlenmeyer flask and shaken at 37 ˚C, 220 rpm. Protein expression was induced when OD_{600} was 0.6 with 0.25 mM IPTG and the strains were grown for another 12 hours at 24 ˚C, 220 rpm. The cells were then spun down and resuspended in a fresh 10 ml LB media to an OD600 of 3.0. *E. coli* strains lacking the TAL enzyme (strains i and ii, see Figure S22) were supplemented with 0.5 mM p-coumaric acid. *E. coli* strains

with the TAL enzyme (strains iii and iv, see Figure S22) were cultivated with 1.0 mM L-Tyrosine and in separate experiments without additional L-Tyrosine. The cultures were left to grow for 3 days at 24 ˚C, 220 rpm. Cells were then spun down by centrifugation (4 ˚C, 20 min, 4,000 x *g*). The supernatant was extracted with 15 ml of chilled ethyl acetate. The solvent was evaporated and the extracted material was then dissolved in ethanol for further HPLC analysis. Quantification of 7-hydroxycoumarin and 8-chloro-7-hydroxycoumarin production was determined using an HPLC system (Agilent Technologies 1260 HPLC system) equipped with an Agilent Zorbax Eclipse Plus C18 4.6 x 100 mm x 3.5 µm HPLC column. The absorbance was measured at 325 nm with a 60 min gradient of 5 – 95 % H_2O/acetonitrile + 0.1% formic acid solvent system.

2. Compound characterisation

2.1 Products by RadH enzymatic reaction. Products were characterised using 1D and 2D NMR, LRMS, HRMS and UV. Larger scale assays for NMR characterisation were carried out to obtain chlorinated products for characterisation using RadH (1.5-6.0 mol%), Fre (0.25-1.0 mol%), substrate (5 mM) in ethanol stock solution, $MgCl_2$ (10 mM), FAD (10 µM), NADH (2-3 equiv.) in 10 mM potassium phosphate buffer, pH 7.4. Assays were run at room temperature. If the reaction did not proceed to sufficient conversion monitored by analytical HPLC after 24 hrs more RadH enzyme was added up to 6 mol%. Reactions were quenched by heat-shock at 95 °C for 5 min and precipitated protein was removed by centrifugation (4°C, 10 min, 12000 x *g*) before analysis by HPLC by the same method as mentioned above.

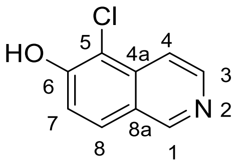

6a

5-chloro-6-hydroxyisoquinoline (6a)[S20] was prepared according to the general procedure described above with 1.5 mol% RadH, 0.25 mol% Fre and 6-hydroxyisoquinoline to give 5-chloro-6-hydroxyisoquinoline (22% yield). λ_{max} (H_2O/CH_3CN) 220 nm, 255 nm and 332 nm; ^1H NMR (400 MHz, MeOD4) δ 9.55 (1H, s, ArH1), 8.53 (1H, d, $J_{3,4}$ = 6.8 Hz, ArH4), 8.49 (1H, d, $J_{3,4}$ = 6.8 Hz, ArH3), 8.36 (1H, d, $J_{7,8}$ = 8.4 Hz, ArH8), 7.96 (1H, d, $J_{7,8}$ = 9.0 Hz, ArH7); ^{13}C NMR (201 MHz, $MeOD_4$, HSQC, HMBC) δ 162.6 (Cq, C6), 147.4 (CH, C1), 140.2 (Cq), 134.0 (CH, C4), 132.9 (CH, C8), 124.6 (CH, C7), 124.4 (Cq), 121.0 (CH, C3), 114.8 (Cq); LRMS-ESI (m/z) 180.1 (^{35}Cl),

182.0 (^{37}Cl) $[M+H]^+$; HRMS-ESI (m/z): calcd for $[M+H]^+$ $C_9H_7NO^{35}Cl$ 180.0216 and $C_9H_7NO^{37}Cl$ 182.0186, found 180.0209 and 182.0225.

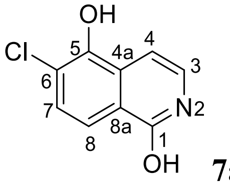

7a

6-chloro-1,5-dihydroxyisoquinoline (7a) was prepared according to the general procedure described above with 6 mol% RadH, 1 mol% Fre and 1.5-dihydroxyisoquinoline to give 6-chloro-1,5-dihydroxyisoquinoline (5% yield). λ_{max} (H_2O/CH_3CN) 210 nm, 243 nm, 282 nm (shoulder), 292 nm, 333 nm and 348 nm; ^{1}H NMR (400 MHz, $MeOD_4$) δ 7.78 (1H, d, $J_{3,4}$ = 8.8 Hz, ArH4), 7.44 (1H, d, $J_{3.4}$ = 8.8 Hz, ArH3), 7.19 (1H, d, $J_{7,8}$ = 7.1 Hz, ArH8), 6.99 (1H, $J_{7.8}$ = 7.1 Hz, ArH7); ^{13}C NMR (201 MHz, $MeOD_4$, HSQC, HMBC) δ 163.0 (Cq), 148.0 (Cq), 130.0 (Cq), 127.5 (CH, C3), 127.3 (CH, C8), 125.4 (Cq), 122.8 (Cq), 118.5 (CH, C4), 100.9 (CH, C7); LRMS-ESI (m/z) 196.1 (^{35}Cl), 198.1 (^{37}Cl) $[M+H]^+$; HRMS-ESI (m/z): calcd for $[M-H]^-$ $C_9H_5NO_2{}^{35}Cl$ 194.0009 and $C_9H_4O_3{}^{37}Cl$ 196.9819, found 194.0003 and 196.9883.

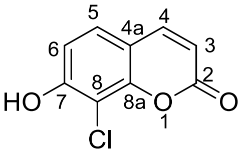

8a

8-chloro-7-hydroxycoumarin (8a) was prepared according to the general procedure described above with 1.5 mol% RadH, 0.25 mol% Fre and 7-hydroxycoumarin to give 8-chloro-7-hydroxycoumarin (31% yield). λ_{max} (H_2O/CH_3CN) 220 nm, 255 (shoulder) nm and 320 nm; ^{1}H NMR (400 MHz, $MeOD_4$) δ 7.77 (1H, d, $J_{3,4}$ = 9.6 Hz, ArH3), 7.31 (1H, d, $J_{5,6}$= 8.7 Hz, ArH6), 6.81 (1H, d, $J_{3,4}$ = 8.7 Hz, ArH5), 6.15 (1H, $J_{4,5}$ = 8.6 Hz, ArH4); ^{13}C NMR (201 MHz, $MeOD_4$, HSQC, HMBC) δ 161.1 (Cq, C2), 157.4 (Cq), 151.4 (Cq), 144.5 (CH, C3), 126.8 (CH, C6), 112.7 (CH, C5), 112.6 (C4), 111.6 (Cq), 107.4 (Cq); LRMS-ESI (m/z) 197.0 (^{35}Cl), 199.0 (^{37}Cl) $[M+H]^+$; HRMS-ESI (m/z): calcd for $[M–H]^-$ $C_9H_4O_3{}^{35}Cl$ 194.9849 and $C_9H_4O_3{}^{37}Cl$ 196.9819, found 194.9849 and 196.9823.

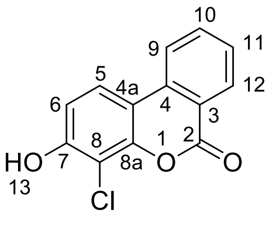

9a

8-chloro-7-hydroxy-3,4-benzocoumarin (9a) was prepared according to the general procedure described above with 0.5 mol% RadH, 0.1 mol% Fre and 7-hydroxy-3,4-benzocoumarin to give 8-

chloro-7-hydroxy-3,4-benzocoumarin (33% yield). λ_{max} (H_2O/CH_3CN) 225 nm, 270 nm and 330 nm; ^{1}H NMR (400 MHz, AcetoneD$_6$) δ 9.59 (1H, s, ArH13), 8.31-8.27 (2H, m, ArH10, ArH11), 8.13 (1H, d, $J_{5,6}$ = 9.0 Hz, ArH5), 7.94-7.90 (1H, m, ArH9,ArH12), 7.65-7.62 (1H, m, ArH9, ArH12), 7.10 (1H, d, $J_{5,6}$ = 9.0 Hz, ArH6); ^{13}C NMR (200 MHz, MeOD$_4$, HSQC, HMBC) δ 160.8 (Cq, C2), 156.5 (Cq), 148.8 (Cq), 136. 4 (CH, C9/12), 135.6 (CH, C9/12), 130.6 (C10/11), 129.16 (CH, C10/11), 123.3 (CH, C5), 119.7 (Cq,), 113.7 (CH, C6), 111.5 (Cq), 108.2 (Cq); LRMS-ESI (m/z) 247.1 (^{35}Cl), 249.1 (^{37}Cl) $[M+H]^+$; HRMS-ESI (m/z): calcd for $[M+H]^+$ $C_{13}H_8O_3{}^{35}Cl$ 247.0162 and $C_{13}H_8O_3{}^{37}Cl$ 249.0132, found 247.0155 and 249.0151.

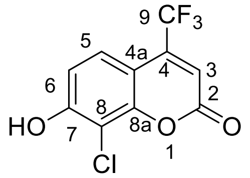

10a

8-chloro-7-hydroxy-4-trifluoromethylcoumarin (10a) was prepared according to the general procedure described above with 6 mol% RadH, 1 mol% Fre and 7-hydroxy-4-trifluoromethycoumarin to give 8-chloro-7-hydroxy-4-trifluoromethylcoumarin (11% yield). λ_{max} (H_2O/CH_3CN) 200 nm, 260 nm and 330 nm; ^{1}H NMR (400 MHz, MeOD$_4$) δ 7.57 (1H, m, ArH5), 7.02 (1H, d, $J_{5,6}$ = 9.0 Hz, ArH6), 6.74 (1H, s, ArH3); ^{13}C NMR (201 MHz, MeOD$_4$, HSQC, HMBC) δ 158.8 (Cq, C2), 158.43 (Cq,), 152.0 (Cq), 141.0 (Cq), 123.8 (Cq, $J_{C,F}$ = 267.5 Hz, 283.4 Hz, 274.5 Hz, C9), 123.6 (CH, C5), 113.2 (CH, C6), 111.8 (CH, C3), 108.4 (Cq), 106.7 (Cq); LRMS-ESI (m/z) 265.0 (^{35}Cl), 267.0 (^{37}Cl) $[M+H]^+$; HRMS-ESI (m/z): calcd for $[M-H]^-$ $C_{10}H_3{}^{35}ClF_3O_3$ 262.9723 and $C_{10}H_3{}^{37}ClF_3O_3$ 264.9693, found 262.9717 and 264.9695.

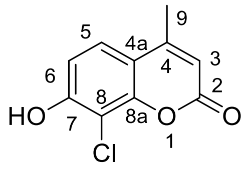

11a

8-chloro-7-hydroxy-4-methycoumarin (11a) was prepared according to the general procedure described above with 6 mol% RadH, 1 mol% Fre and 7-hydroxy-4-methylcoumarin to give 8-chloro-7-hydroxy-4-methycoumarin (17% yield). λ_{max} (H_2O/CH_3CN) 210 nm, 250 nm (shoulder) and 320 nm; ^{1}H NMR (400 MHz, MeOD$_4$) δ 7.59 (1H, d, $J_{5,6}$ = 8.8 Hz, ArH6), 6.97 (1H, d, $J_{5,6}$ = 8.6 Hz, ArH5), 6.19 (1H, s, ArH3), 2.45 (3H, s, H9); ^{13}C NMR (201 MHz, MeOD$_4$, HSQC, HMBC) δ 161.2 (Cq, C2), 157.4 (Cq,), 154.4 (Cq), 127.5 (Cq), 150.8 (Cq), 123.5 (CH, C5), 113.3 (Cq), 112.5 (CH, C6), 110.2 (CH, C3), 107.6 (Cq), 17.2 (C9); LRMS-ESI (m/z) 211 (^{35}Cl), 213

(^{37}Cl) $[M+H]^+$; HRMS-ESI (m/z): calcd for $[M+H]^+$ $C_{10}H_8{}^{35}ClO_3$ 211.0162 and $C_{10}H_8{}^{37}ClO_3$ 213.0132 found 211.0164 and 213.0151.

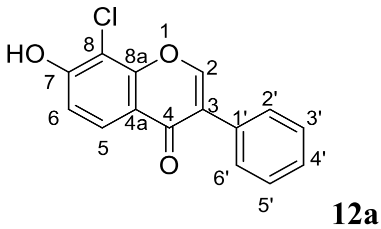

12a

8-chloro-7-hydroxy-3-phenyl-4H-chromen-4-one (12a) was prepared according to the general procedure described above with 0.5 mol% RadH, 0.1 mol% Fre and 7-hydroxy-3-phenyl-4H-chromen-4-one to give 8-chloro-7-hydroxy-3-phenyl-4H-chromen-4-one (16% yield). λ_{max} (H_2O/CH_3CN) 225 nm, 270 nm and 330 nm; ^{1}H NMR (400 MHz, $MeOD_4$) δ 8.14-8.13 (2H, s, ArH2'/6'), 7.96 (1H, d, $J_{5,6}$ = 8.8 Hz ArH5), 7.65-7.60 (3H, m, ArH3'/4'/5'), 7.10 (1H, d, $J_{5,6}$ = 8.8 Hz, ArH6), 6.93 (1H, s, ArH2); ^{13}C NMR (201 MHz, $MeOD_4$, HSQC, HMBC) δ 178.4 (Cq, C4), 163.7 (Cq), 153.8 (Cq,), 134.7 (Cq), 131.7 (CH, C4'), 128.9 (CH, C3'/5'), 126.1 (CH, C2'/C6'), 123.9 (CH, C5), 123.8 (Cq), 116.9 (Cq), 114.7 (CH, C6), 107.9 (Cq), 105.8 (CH, C2); LRMS-ESI (m/z) 271.0 (^{35}Cl), 273.0 (^{37}Cl) $[M-H]^-$; HRMS-ESI (m/z): calcd for $[M-H]^-$ $C_{15}H_8O_3{}^{35}Cl$ 271.0167 and $C_{15}H_9O_3Cl^{37}$ 273.0132, found 271.0157 and 273.0128.

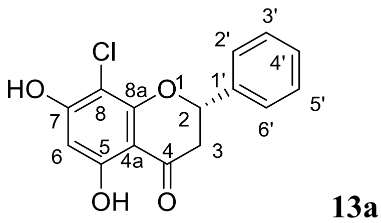

13a

8-Chloro- 5,7-dihydroxy-2-phenylchroman-4-one (8-chloropinocembrin) (13a) was prepared according to the general procedure described above with 6 mol% RadH, 1 mol% Fre and pinocembrin to give 8-chloropinocembrin (35% yield). λ_{max} (H_2O/CH_3CN) 289, 325 nm; ^{1}H NMR (400 MHz, $CDCl_3$) δ 7.71-7.55 (2H, m, ArH2'/6'), 7.48-7.40 (3H, m, ArH3'-5'), 6.08 (1H, s, ArH6), 5.62 (1H, dd, J = 12.3, 3.2 Hz, H2), 3.18 (1H, dd, J = 17.2, 12.4 Hz, H3), 2.92 (1H, dd, , J = 17.2, 3.3, H3); ^{13}C NMR (100 MHz, $MeOD_4$, HSQC, HMBC) δ 195.6 (Cq, C4), 162.4 (Cq), 161.8 (Cq), 158.0 (Cq), 138.6. (Cq), 128.4 (CH, C4'), 128.3 (2 CH, C3',C5'), 125.9 (2CH, C2', C6'), 102.4 (Cq), 99.5 (Cq), 95.9 (CH, C6), 79.5 (CH, C2), 42.3 (CH2, C3) LRMS-ESI (m/z), $[M-H]^-$ 298.01, HRMS-ESI (m/z): calcd for $[M+H]^+$ $C_{15}H_{12}O_4{}^{35}Cl$ 291.0419 and $C_{15}H_{12}O_4{}^{37}Cl$ 293.0394, found 291.0412 and 293.0383.

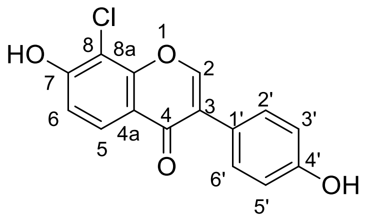

14a

8-chloro-7-hydroxy-3-(4-hydroxyphenyl)-4H-chromen-4-one (14a) was prepared according to the general procedure described above with 0.5 mol% RadH, 0.1 mol% Fre and 7-hydroxy-3-(4-hydroxyphenyl)-4H-chromen-4-one to give 8-chloro-7-hydroxy-3-(4-hydroxyphenyl)-4H-chromen-4-one (20% yield). λ_{max} (H_2O/CH_3CN) 260 nm and 315 nm; ^1H NMR (400 MHz, $MeOD_4$) δ 8.25 (1H, s, ArH2), 7.97 (1H, d, $J_{5,6}$ = 9.0 Hz ArH5), 7.42-7.40 (2H, m, ArH2'/6'), 7.01 (1H, d, $J_{5,6}$ = 9.0 Hz, ArH6), 6.88-6.86 (2H, m, ArH3'/5'); ^{13}C NMR (201 MHz, $MeOD_4$, HSQC, HMBC) δ 176.4 (Cq, C4), 161.9 (Cq), 157.4 (Cq), 154.2 (Cq), 152.9 (Cq), 136.8 (Cq), 130.1 (CH, C2'/C6'), 124.9 (Cq), 124.2 (CH, C5), 122.6 (Cq), 116.2 (Cq), 115.9 (CH, C6); 114.8 (CH, C3'/5'), 107.48 (CH, C2); LRMS-ESI (m/z) 287.0 (^{35}Cl), 289.0 (^{37}Cl) $[M-H]^-$; HRMS-ESI (m/z): calcd for $[M+H]^+$ $C_{15}H_{10}O_4{}^{35}Cl$ 289.0262 and $C_{15}H_{10}O_4{}^{37}Cl$ 291.0238, found 289.0262 and 291.0230

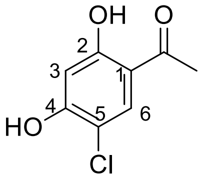

15a

1-(5-chloro-2,4-dihydroxyphenyl)ethanone (15a)[S21] was prepared according to the general procedure described above with 6 mol% RadH, 1 mol% Fre and 1-(2,4-dihydroxyphenyl)ethanone to give 1-(5-chloro-2,4-dihydroxyphenyl)ethanone (5% yield).

λ_{max} (H_2O/CH_3CN) 225nm and 285 nm; ^1H NMR (400 MHz, $MeOD_4$) δ 7.82 (1H, s, ArH), 6.37(1H,s, ArH), 2.54 (3H, s, CH_3); LCMS $[M+H]^+$ calculated for $C_8H_6ClO_3$ 187.0, found 187.0; HRMS-ESI (m/z): calcd for $[M-H]^-$ $C_8H_6O_3{}^{35}Cl$ 185.0005 and $C_8H_6O_3{}^{37}Cl$ 186.9978 found 185.0008 and 187.0015.

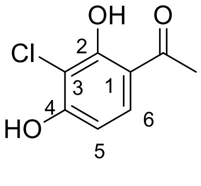

15b

1-(3-chloro-2,4-dihydroxyphenyl)ethanone (15b)[S20] was prepared according to the general procedure described above with 6 mol% RadH, 1 mol% Fre and 1-(2,4 dihydroxyphenyl)-ethanone to give 1-(3-chloro-2,4-dihydroxyphenyl)ethanone (10% yield). λ_{max} (H_2O/CH_3CN) 225nm, 235, 270 nm and 330 nm; ^1H NMR (400 MHz, $MeOD_4$) 7 .70 (1H, d, J = 9.0 Hz, ArH), 6.51(1H, d, J = 9.0 Hz, ArH), 2.57 (3H, s, CH_3); LCMS $[M+H]^+$ calculated for $C_8H_8ClO_3$ 187.0, found 187.0;

HRMS-ESI (m/z): calcd for $[M-H]^-$ $C_8H_6O_3{}^{35}Cl$ 185.0005 and $C_8H_6O_3{}^{37}Cl$ 186.9978 found 185.0006 and 186.9992.

2.2 Synthesis of Substrates & Product Standards

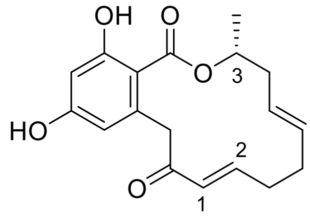

1

(R)-14,16-dihydroxy-3-methyl-3,4,7,8-tetrahydro-1H-benzo[c][1]oxacyclotetradecine-1,11(12H)-dione (1)S23 was synthesised as a colourless solid using literature known procedures starting from orsellinic acid. ^{1}H NMR (500 MHz, Acetone-d_6): δ 11.52 (1H, br. s, ArO*H*), 9.25 (1H, br. s, ArO*H*), 6.61-6.67 (1H, m, C2-*H*), 6.33 (1H, d, *J* = 2.5 Hz, Ar*H*), 6.32 (1H, d, *J* = 2.5 Hz, Ar*H*), 5.83 (1H, d, *J* = 15.5 Hz, C1-*H*), 5.23-5.36 (3H, m, 2 x alkene & C3-*H*), 4.06 (1H, d, *J* = 17.0 Hz, ArCH_2), 3.89 (1H, d, *J* = 17.0 Hz, ArCH_2), 2.65 (1H, ddd, *J* = 14.5, 8.0, 4.0 Hz, methylene), 2.13-2.30 (5H, m, methylene), 1.30 (3H, d, *J* = 6.5 Hz, methyl); ^{13}C NMR (126 MHz, CD_2Cl_2) δ 198.2 (ArCH_2*C*O), 170.6 (Ar*C*OOR), 166.1(ArC_q), 161.5 (ArC_q), 148.4 (*C*2), 140.6 (ArC_q), 132.5 (ArC_q), 130.6, (*C*1), 127.6 (alkene), 113.0 (Ar*C*H), 106.4 (Alkene), 103.1 (Ar*C*H), 73.0 (*C*3), 49.1 (Ar*C*H_2), 37.4 (*C*H_2), 31.7 (*C*H_2), 31.6 (*C*H_2), 18.6 (*C*H_3); LRMS-ESI $[M-H]^-$ 315.1; HRMS-ESI (m/z): calcd for $[M-H]^-$ $C_{18}H_{19}O_5$ 315.1238, found 315.1232.

8-bromo-7-hydroxycoumarin (8b)[S24] A 100 mL three-necked round bottom flask was equipped with a reflux condenser and 2-bromoresorcinol (0.60 g, 3.17 mmol) and propiolic acid (0.39 mL, 6.28 mmol). The resulting solution was stirred and heated to 110°C. Subsequently three drops of concentrated H_2SO_4 was added and stirred for 30 minutes and the reaction was monitored via TLC. The resultant red waxy product was neutralized with saturated $NaHCO_3$, washed with EtOAc, dried over $MgSO_4$ and filtered. After purification via HPLC (5%-75% ACN/water) 8-bromo-7-hydroxycoumarin was obtained as colourless powder (0.41 g, 54%); R_f (Hexane:EtOAc 1:1): 0.47; ^{1}H NMR (400 MHz, Acetone-d$_6$) δ 11.48 (1H, s, OH), 8.04 (1H, d, $J_{5,6}$ = 8.6 Hz, ArH3), 7.61 (1H, d, $J_{5,6}$ = 9.5 Hz, ArH6), 7.03 (1H, d, $J_{5,6}$ = 9.5 Hz, ArH5), 6.35 (1H, d, $J_{5,6}$ = 8.6Hz, ArH4); ^{13}C NMR (100 MHz, $MeOD_4$, HMQC, HMBC) δ 159.8 (Cq, C21), 158.2 (Cq), 152.1 (Cq), 144.5 (CH, C3), 128.3 (CH, C6), 112.7 (CH, C5), 112.3 (Cq); 111.8 (CH, C4), 96.7 (Cq); LRMS-ESI (m/z) 241.0 (^{79}Br), 242.9 (^{81}Br) $[M+H]^+$; HRMS-ESI (m/z): calcd for $[M+Na]^+$ $C_9H_5{}^{79}BrO_3$ 262.9320 and

$C_9H_5{}^{81}BrO_3$ 264.92993 found 262.9320 and 264.9300. IR: υ_{max}/cm^{-1}; 3086.56 (O-H), 1675.09 (C=O ester), 1597.41, 1541.88 (C=C), 1234.52, 1306.02 (C-O).

7-hydroxy-3,4-benzocoumarin (9)[S25] 2-bromobenzoic acid (2.01g, 10 mmol) and resorcinol (2.20 g, 20 mmol) were heated under nitrogen atmosphere to 60 °C for 15 minutes. Deoxygenated aq. $CuSO_4$ (10%, 0.50 mL) was added drop wise to the reaction mixture. Upon addition of aq. $CuSO_4$ the solution went from blue to brown and was heated for another 3 hours and then stirred overnight to afford the crude product. Recrystallization in acetic acid and further purification by flash chromatography gave the pure product as cream powder (0.302g, 15%); R*f* (Hexane:Petether 1:1): 0.64; ^{1}H NMR (400 MHz, DMSO-d$_6$) δ 9.15 (1H, s, ArH13), 8.14-8.09 (2H, m, ArH10, ArH11), 8.01 (1H, d, $J_{5,6}$ = 8.7 Hz, ArH5, ArH6), 7.76-7.72 (1H, m, ArH5,ArH6), 7.46-7.42 (1H, m, ArH9, ArH12), 6.79 (1H, dd, $J_{9,10}$ = 8.0 Hz, $J_{9,11}$ = 2.4 Hz, ArH9, ArH12), 6.68 (1H,d, $J_{5,6}$ = 8.0 Hz, ArH8); ^{13}C NMR (100 MHz, MeOD$_4$, HMQC, HMBC) δ 160.6 (Cq, C2), 159.8 (Cq), 152.1 (Cq,), 135.3 (CH), 135.1 (CH), 129.7 (CH), 127.6 (CH); 124.8 (CH, C9/12), 121.6 (Cq, C3/4), 118.9 (CH, C8) 113.3 (CH), 109.4(Cq), 102.9 (Cq); LRMS-ESI (m/z) 235.1 $[M+Na]^+$; HRMS-ESI (m/z): calcd for $[M+Na]^+$ $C_{13}H_8O_3Na$ 235.0371 found 235.0372 and 249.0151; υ_{max}/cm^{-1} 3291.00 (O-H), 3105.20 (C-H), 1694.66 (C=O ester), 1626.77, 1608.97 (C=C), 1317.64, 1276.89 (C-O).

8-chloro-7-hydroxy-3,4-benzocoumarin (9a) 2-bromobenzoic acid (2.0 1g, 10 mmol) and 2-chlororesorcinol (2.89 g, 20 mmol) were heated under nitrogen atmosphere to 60 °C for 15 minutes and then deoxygenated aq. $CuSO_4$ (10%, 0.5 mL) was added drop wise to the reaction mixture. Upon addition of 10% aq. $CuSO_4$ the solution went from orange to brown. After reflux for 3 hours the solution turned dark red and the product was yielded after stirring over night at room temperature as a cream powder which was further purified by flash chromatography. (0.90 g, 45%); R_f (Hexane:EtOAc 1:1): 0.48; ^{1}H NMR (100, AcetoneD$_6$) δ 9.59 (1H, s, ArH13), 8.24-8.33 (2H, m, ArH10, ArH11), 8.13 (1H, d, $J_{5,6}$ = 9.0 Hz, ArH5, ArH6), 7.89-7.95 (1H, m, ArH9,ArH12), 7.65-7.61 (1H, m, ArH9, ArH12), 7.10 (1H, d, $J_{5,6}$ = 9.0 Hz, ArH5, ArH6); ^{13}C NMR (400 MHz, MeOD$_4$, HMQC, HMBC) δ 160.8 (Cq, C2), 156.5 (Cq, C), 148.8 (Cq,), 136. 4 (CH), 135.6 (CH), 130.6 (CH), 129.16 (CH); 123.3 (CH, C9/12), 119.7 (Cq, C3/4), 113.7 (CH), 111.5 (Cq), 108.2 (Cq); LRMS-ESI (m/z) 247.1 (^{35}Cl), 249.1 (^{37}Cl) [M+H]; HRMS-ESI (m/z): calcd for $[M+H]^+$ $C_{13}H_8O_3{}^{35}Cl$ 247.0162 and $C_{13}H_8O_3{}^{37}Cl$ 249.8810 found 247.0166 and 249.0151; υ_{max}/cm^{-1}; 3245.75 (O-H), 2973.95 (C-H), 1693.48 (C=O ester), 1607.73, 1565.41 (C=C), 1323.05 (C-O).

Supplementary references

S1. Ren, W., Qiao, Z. H., Wang, H., Zhu, L., Zhang, L. Flavonoids: promising anticancer agents. *Med. Res. Rev.* 23, 519-534 (2003).

S2. Ciustea, M, Ciustea, G. Combination therapy and methods for treatment and prevention of hyperproliferative diseases, US 20130045179 A1, 21. February 2013.

S3. Celmer Walter D, Kenneth Koe Billie Adducts of salts of novobiocin, US 2891051 A 16. June 1959.

S4. Kontogiorgis, C., Detsi, A., Hadjipavlou-Litina, D. Expert Opinion on Therapeutic Patent, **22**, 437-454 (2012).

S5. Yamaguchi T., Hempson S.J., Reif G. A. J. *et al.* Calcium restores a normal proliferation phenotype in human polycystic kidney disease epithelial cells. *Am. Soc. Nephrol.* **17**, 178-187 (2006).

S6. (1) Albrecht, B. K. *et al.* Therapeutic comounds and uses therof, WO 2016123387 A1, 4. August 2016. (2) Luo, P**.** Method of preparing hydroxyapatite based drug delivery implant for infection and cancer treatment US 20070190102 A1, 16. August 2007.

S7. Abdelmohsen, K., Stuhlmann, D., Daubrawa, F., Klotz, L. O. Dicumarol is a potent reversible inhibitor of gap junctional intercellular communication. *Arch. Biochem. Biophys*. 15, 241-247 (2005).

S8. Down, B. Granule formation, US8007830 B2, 30. August 2011.

S9. Francis, W. Use of (S)-(+)-Hydroxychloroquine, EP0588430 B1, 27. May 1998.

S10. Marie, S. Preparation of quinapril hydrochloride, EP 1611106 A1, 4. January 2006.

S11. Wang, G., Burow, M. E, Boue, S. M., Wiese, T. E., Jiang, Q. Novel daidzein analogs as treatment for cancer, US 20130184475 A1, 18. July 2013.

S12. Berg, A. Improved microbial production of anthracyclins,WO 2006111561 A1, 26. October 2006.

S13. Trefzer, A. *et al.* Biosynthetic gene cluster of simocyclinone, a natural multihybrid antibiotic. *Antimicrob. Agents Chemother*. **46**, 1174-1182 (2002).

S14. Anderle, C., Li, S.-M., Kammerer, B., Gust, B. & Heide, L. New aminocoumarin antibiotics derived from 4-hydroxycinnamic acid are formed after heterologous expression of a modified clorobiocin biosynthetic gene cluster. *J. Antibiot.* **60**, 504-510 (2007).

S15. Shepherd, S. A. *et al.* Extending the biocatalytic Scope of regiocomplementary flavin-dependent halogenase enzymes. *Chem. Sci.* **6**, 3454-3460 (2015).

S16. Schwede, T., Kopp, J., Guex, N., Peitsch, M.C. SWISS-MODEL: An automated protein homology-modeling server. *Nucleic Acids Res*. **31**, 3381-3385 (2003).

S17 Arnold, K., Bordoli, L., Kopp, J., Schwede, T. he SWISS-MODEL workspace: a web-based environment for protein structure homology modelling. *Bioinformatics* **22**,195-201 (2006).

S18. Trott, O., Olson, A. J. AutoDock Vina: improving the speed and accuracy of docking with a new scoring function, efficient optimization, and multithreading. *J. Comput. Chem*. **31**, 455-461 (2010).

S19. Feher, T. *et al.* Validation of RetroPath, a computer-aided design tool for metabolic pathway engineering. *Biotechnol. J.* **9**, 1446-1457 (2014).

S20. Zeng, J., Lytle, A. K., Gage, D., Johnson, S. J. & Zhan, J. Specific chlorination of isoquinolines by a fungal flavin-dependent halogenase Bioorg. *Med. Chem. Lett.* **23**, 1001-1003 (2013).

S21. Chen, D. *et a*l. Discovery of potent *N*-(isoxazol-5-yl)amides as HSP90 inhibitors. *Eur.J. Med. Chem.* **87**, 765 – 781 (2014).

S22. Jeong, C. H. *et al.* Discovery of hybrid Hsp90 inhibitors and their anti neoplastic effects against gefitinib-resistant non-small cell lung cancer (NSCLC). Bioorg*. Med .Chem. Lett.* **24**, 224-227 (2014).

S23. Moulin, E., Barluenga, S., Winssinger, N. Concise synthesis of pochonin A, an HSP90 inhibitor. *Org. Lett.* **7**, 5637-5639 (2005).

S24. Pardanani. N. H., Trivedi, K. N. Studies in the synthesis of furocoumarins. XIV. Synthesis of psoralene from 2-bromoresorcinol. *Aust. J. Chem.* **25**, 1537-1542 (1972).

S25. Krzeszewski, M., Vakuliuk, O., Gryko, D. T. Color-Tunable Fluorescent Dyes Based on Benzo[*c*]coumarin. *Eur. J. Org. Chem.* **2013**, 5631-5644 (2013).

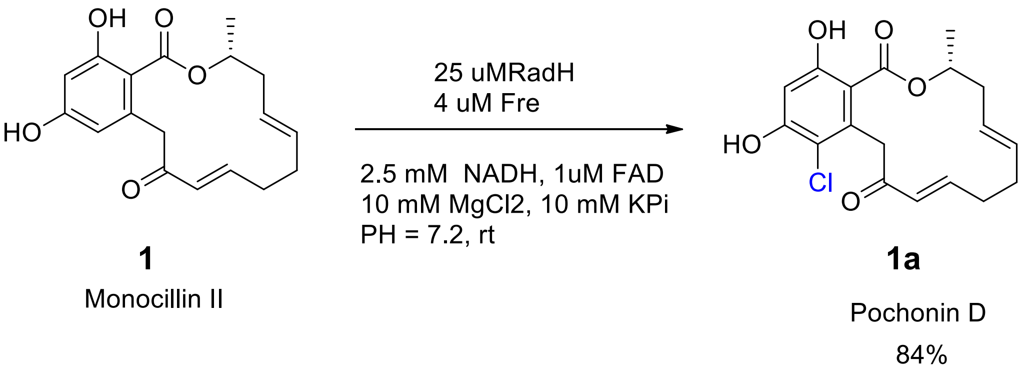

1

Monocillin II

1a

Pochonin D

84%

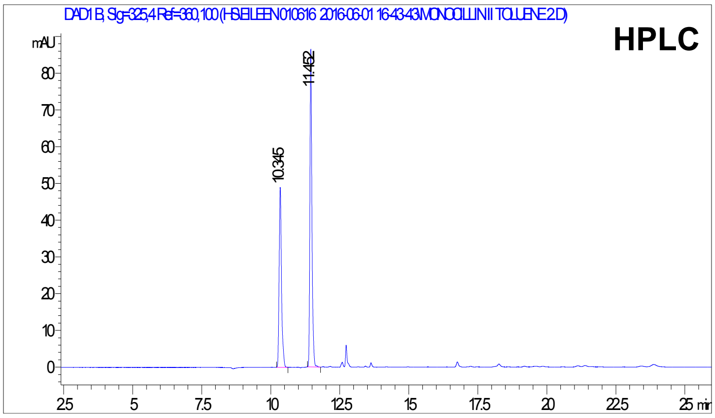

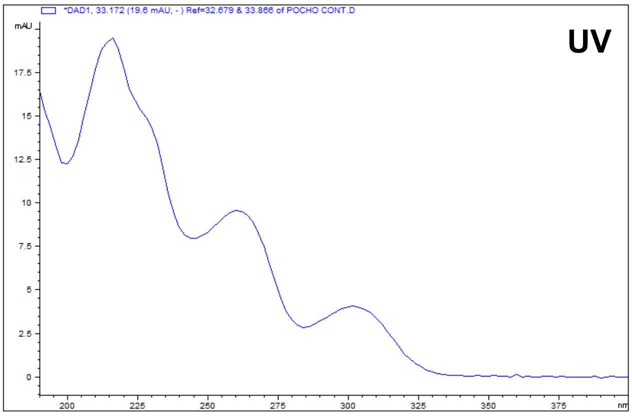

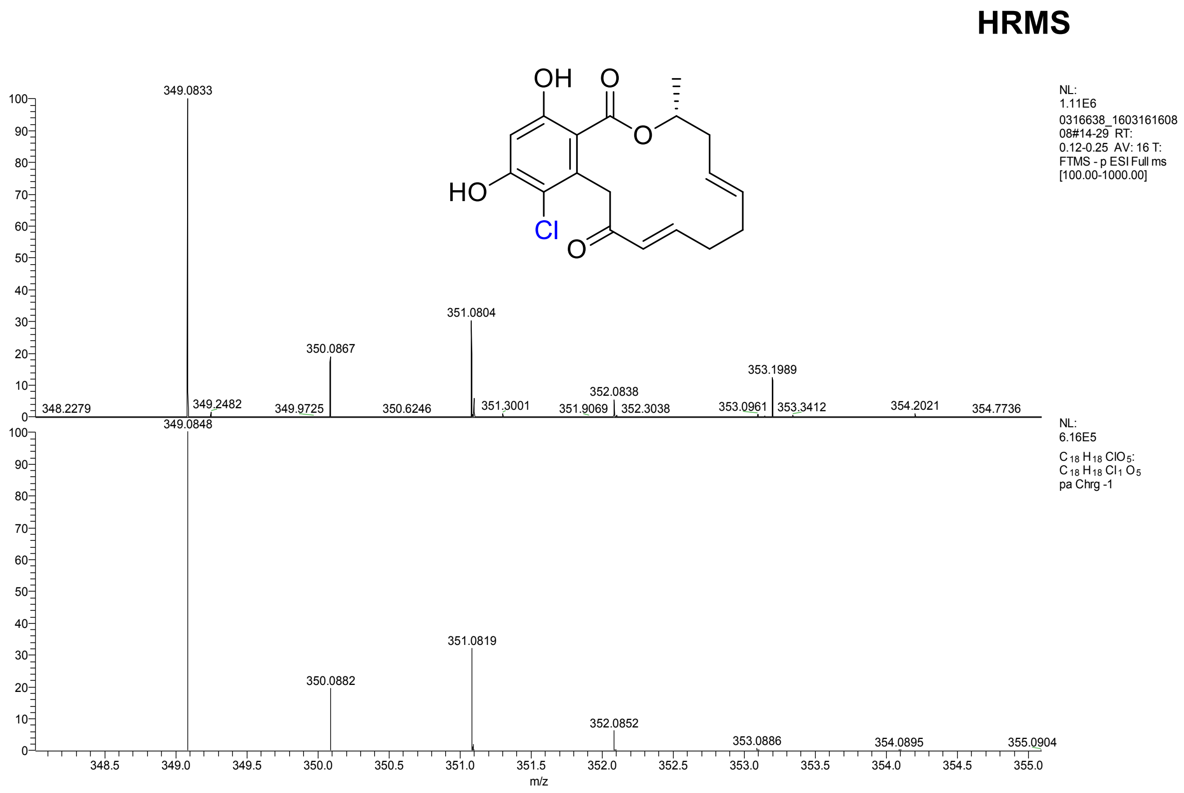

HPLC trace, UV spectrum and high resolution mass of pochonin D (**1a**).

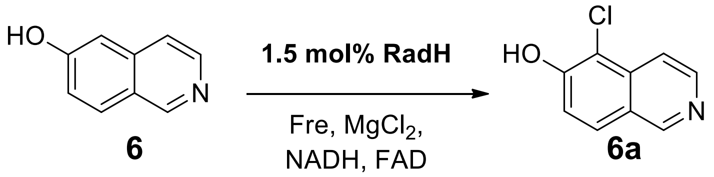

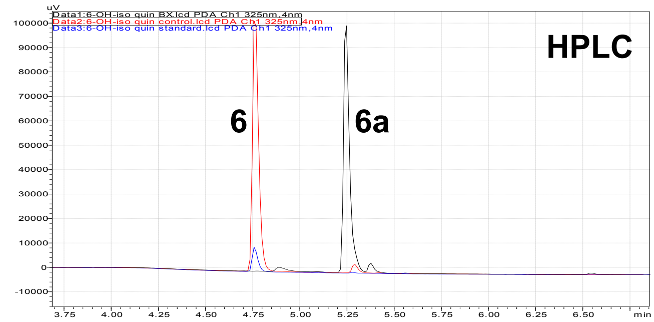

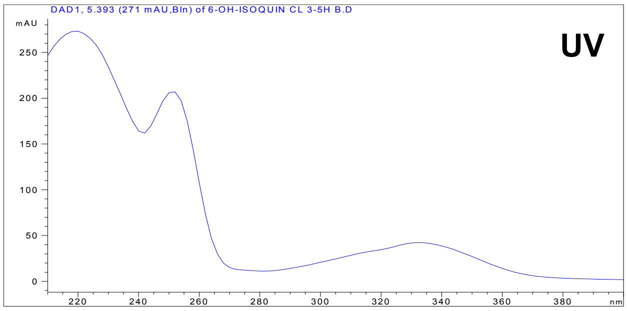

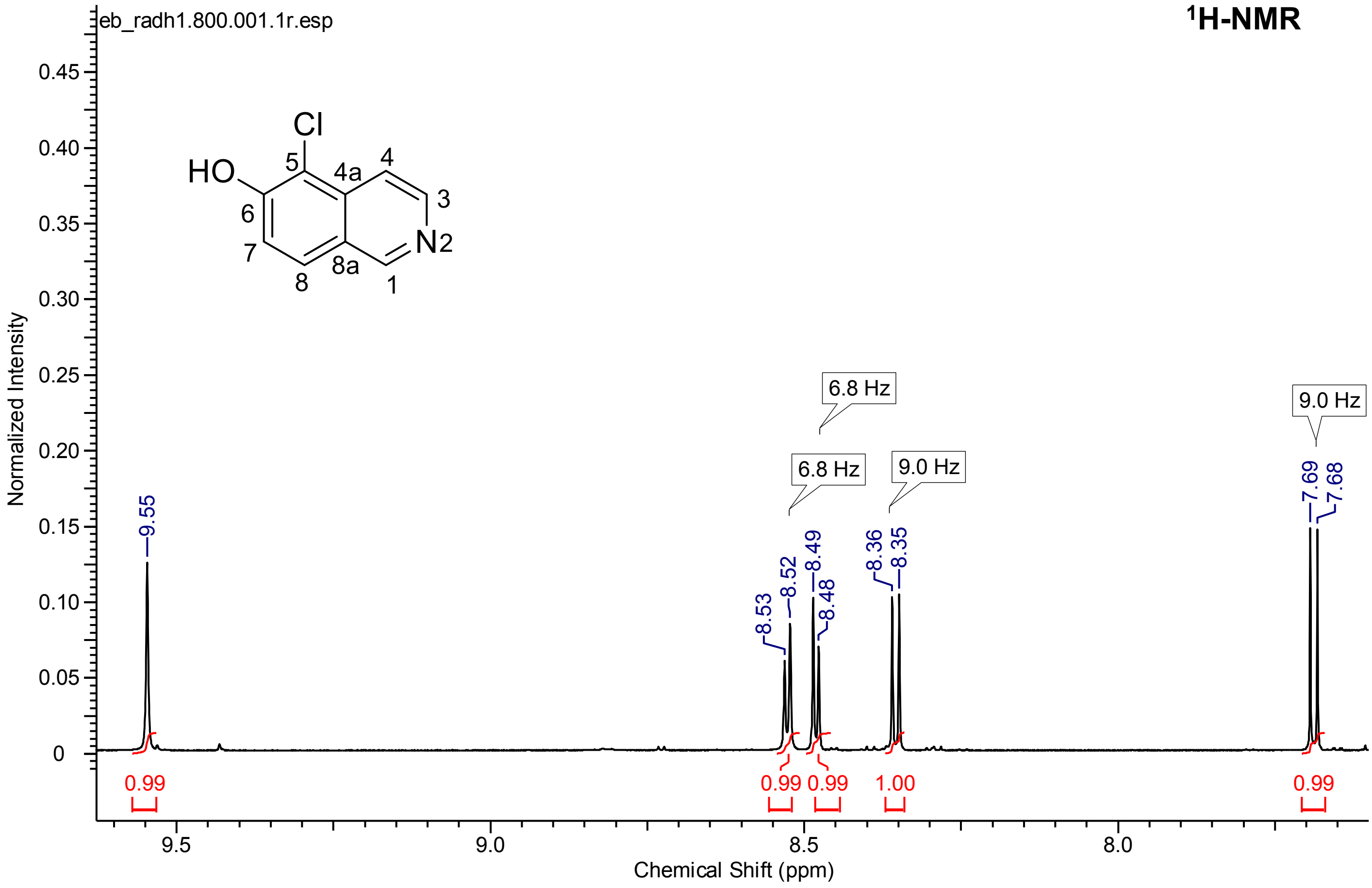

HPLC trace, UV spectrum ^{1}H NMR of 5-chloro-6-hydroxyisoquinoline (**6a**).

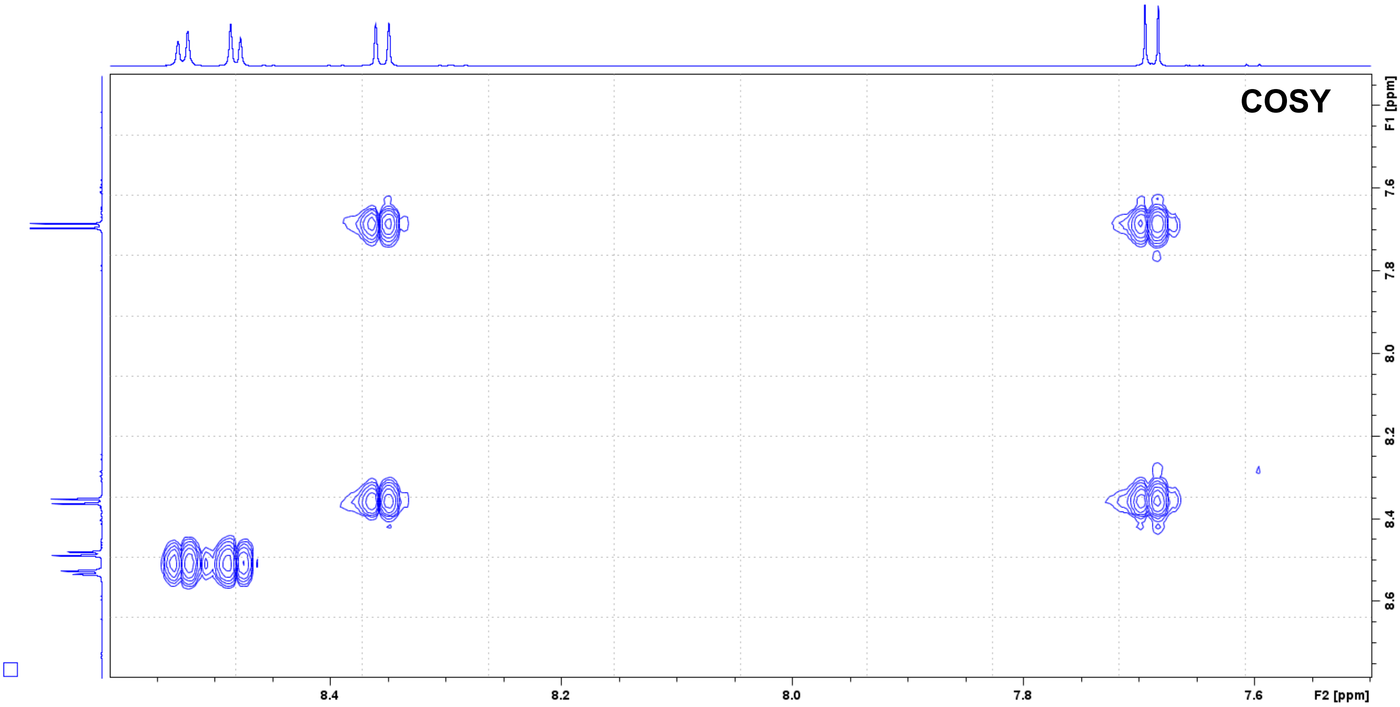

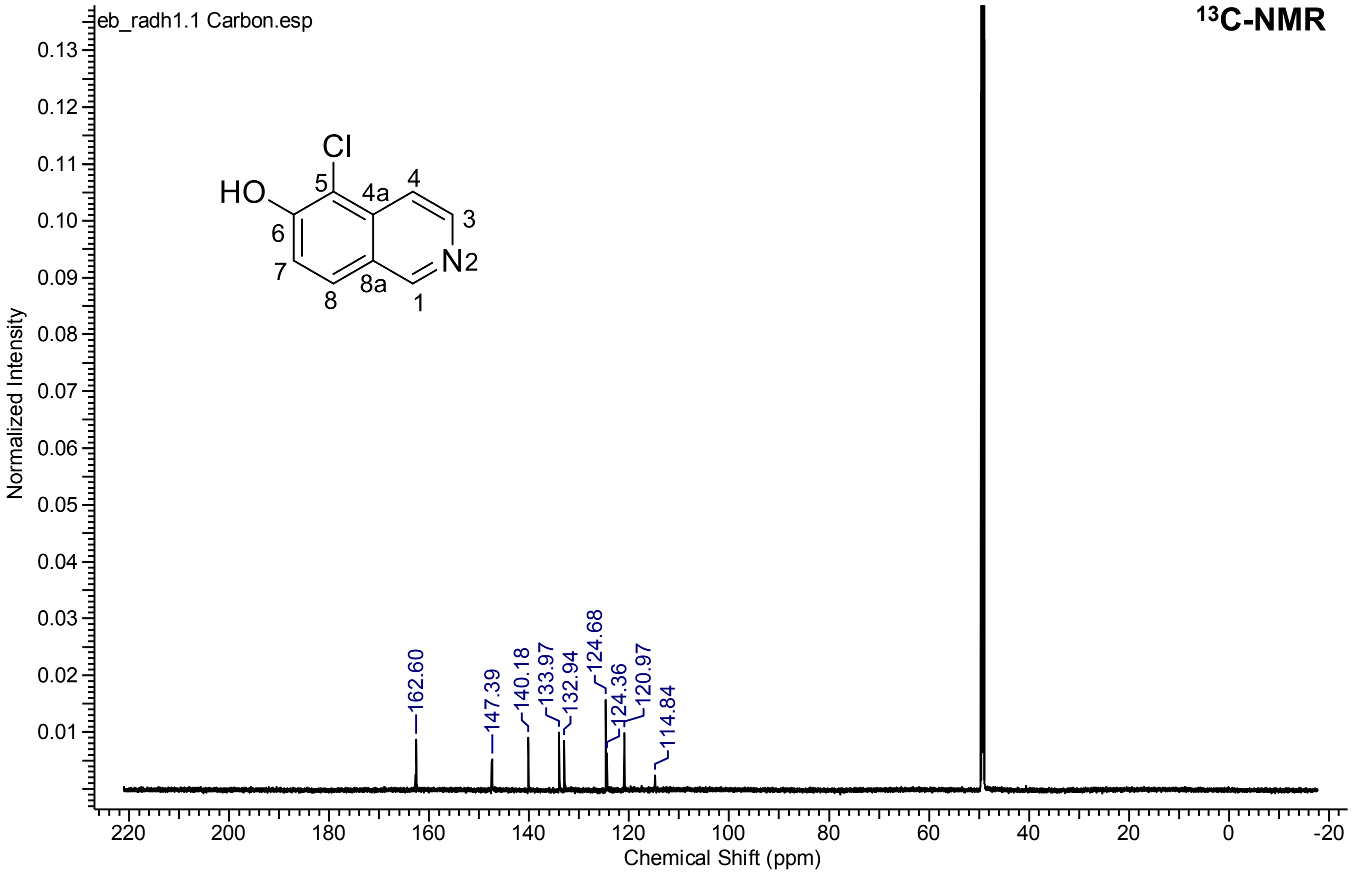

COSY and ^{13}C NMR of 5-chloro-6-hydroxyisoquinoline (**6a**).

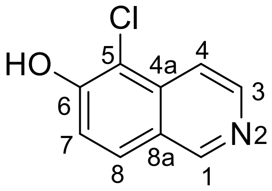

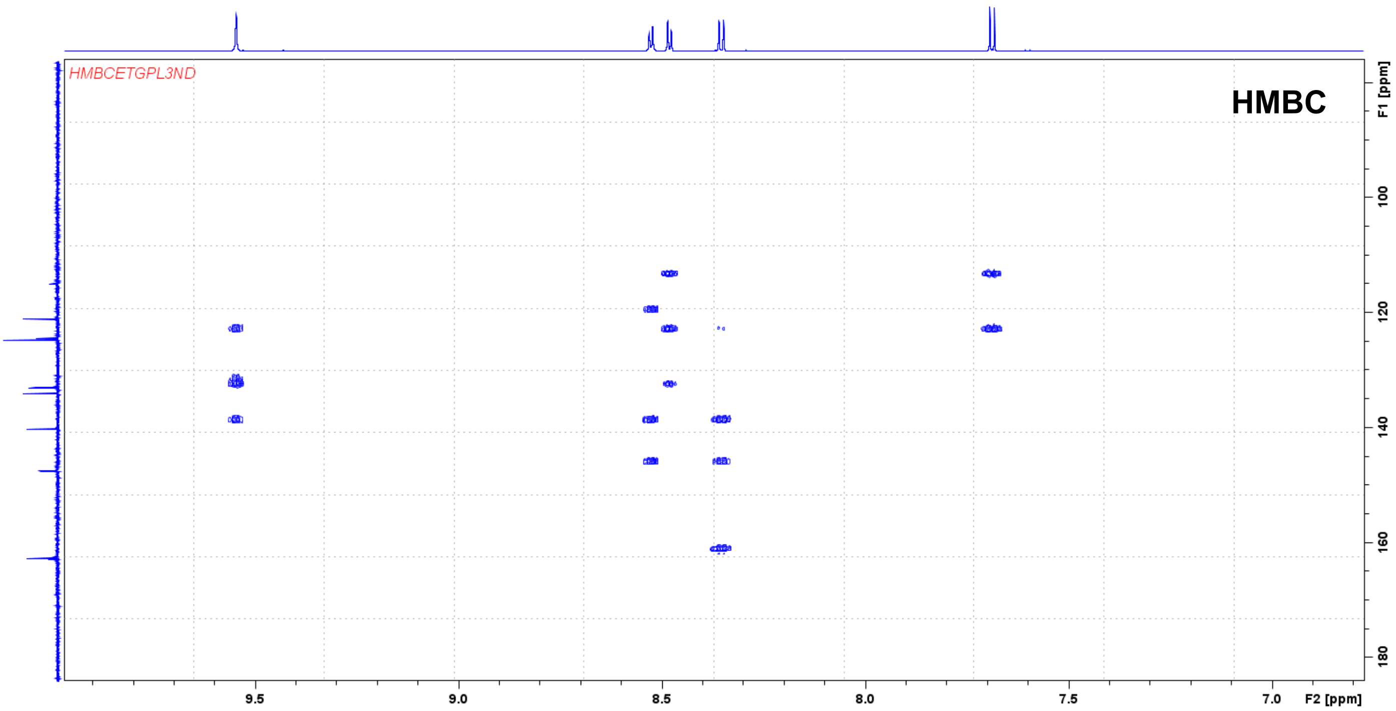

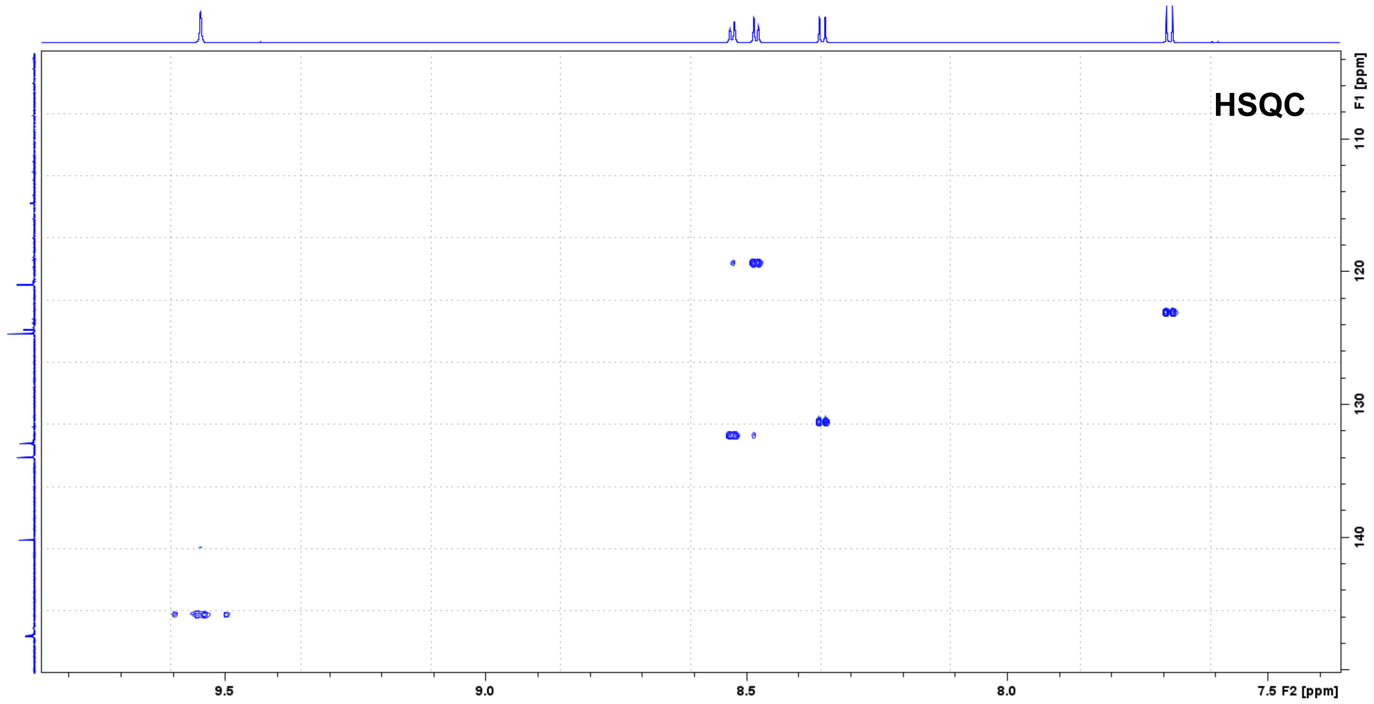

HMBC and HSQC of 5-chloro-6-hydroxyisoquinoline (**6a**).

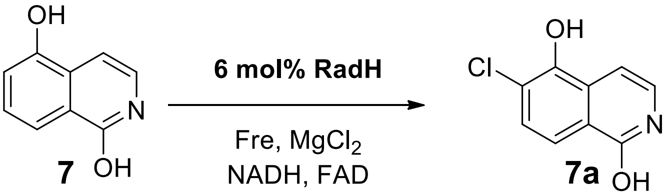

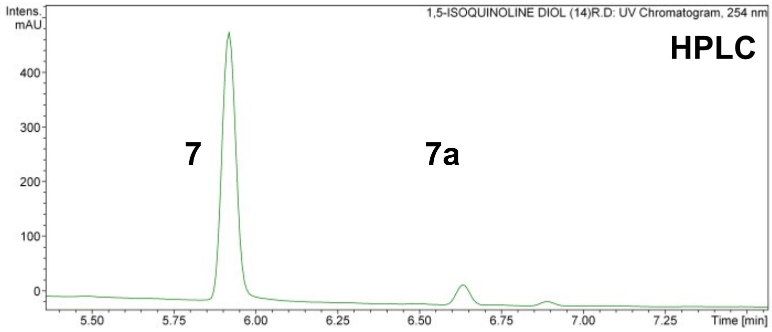

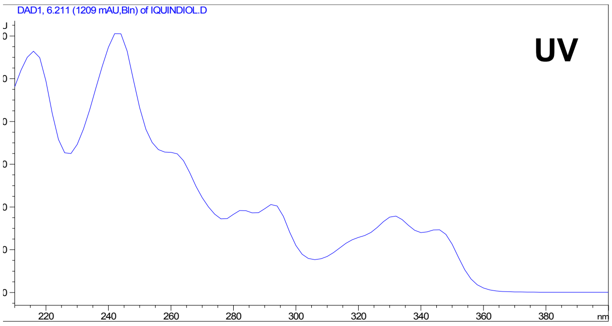

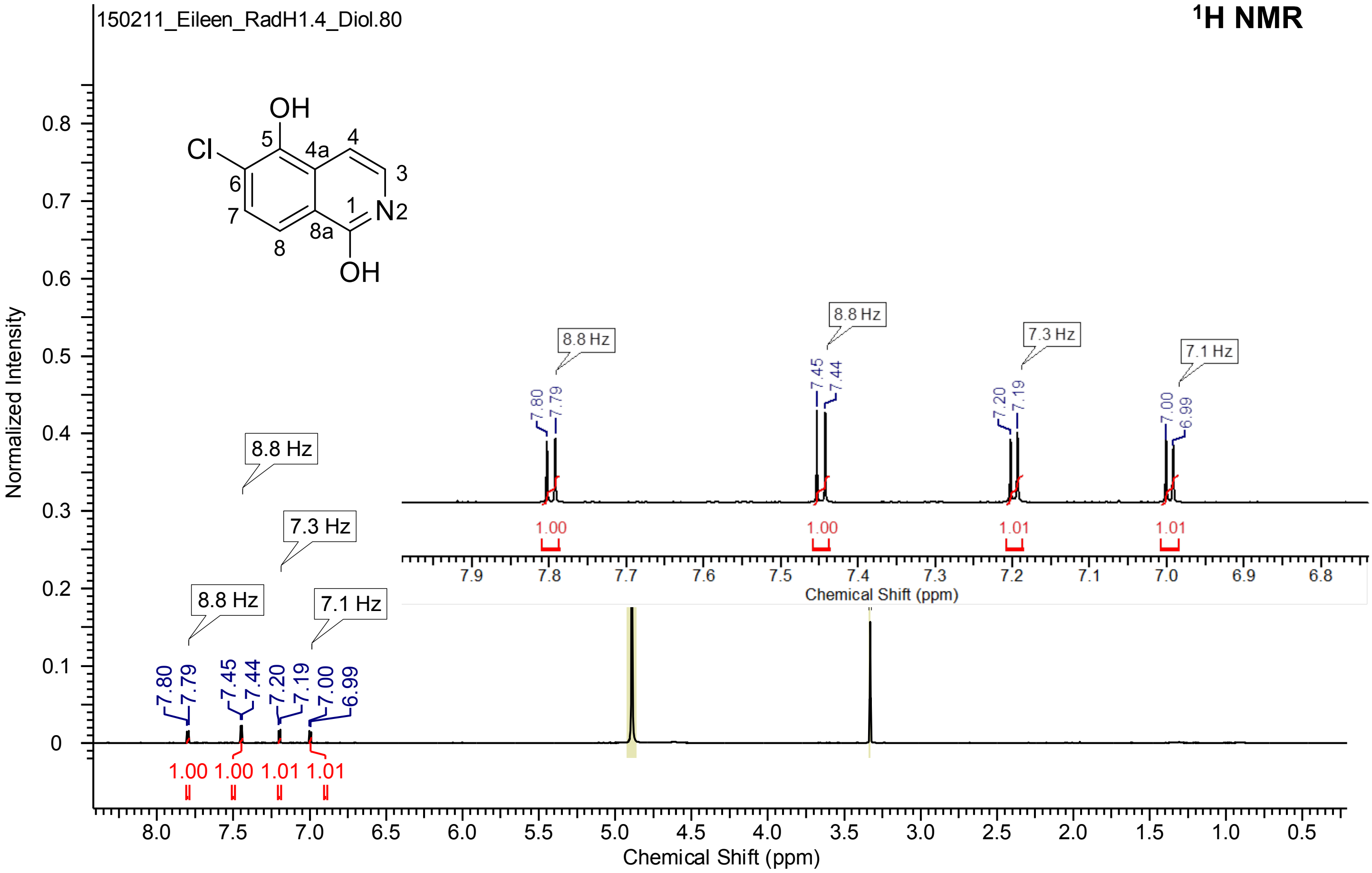

HPLC trace, UV spectrum and ^{1}H NMR of 6-chloro-1,5-dihydroxyisoquinoline (**7a**).

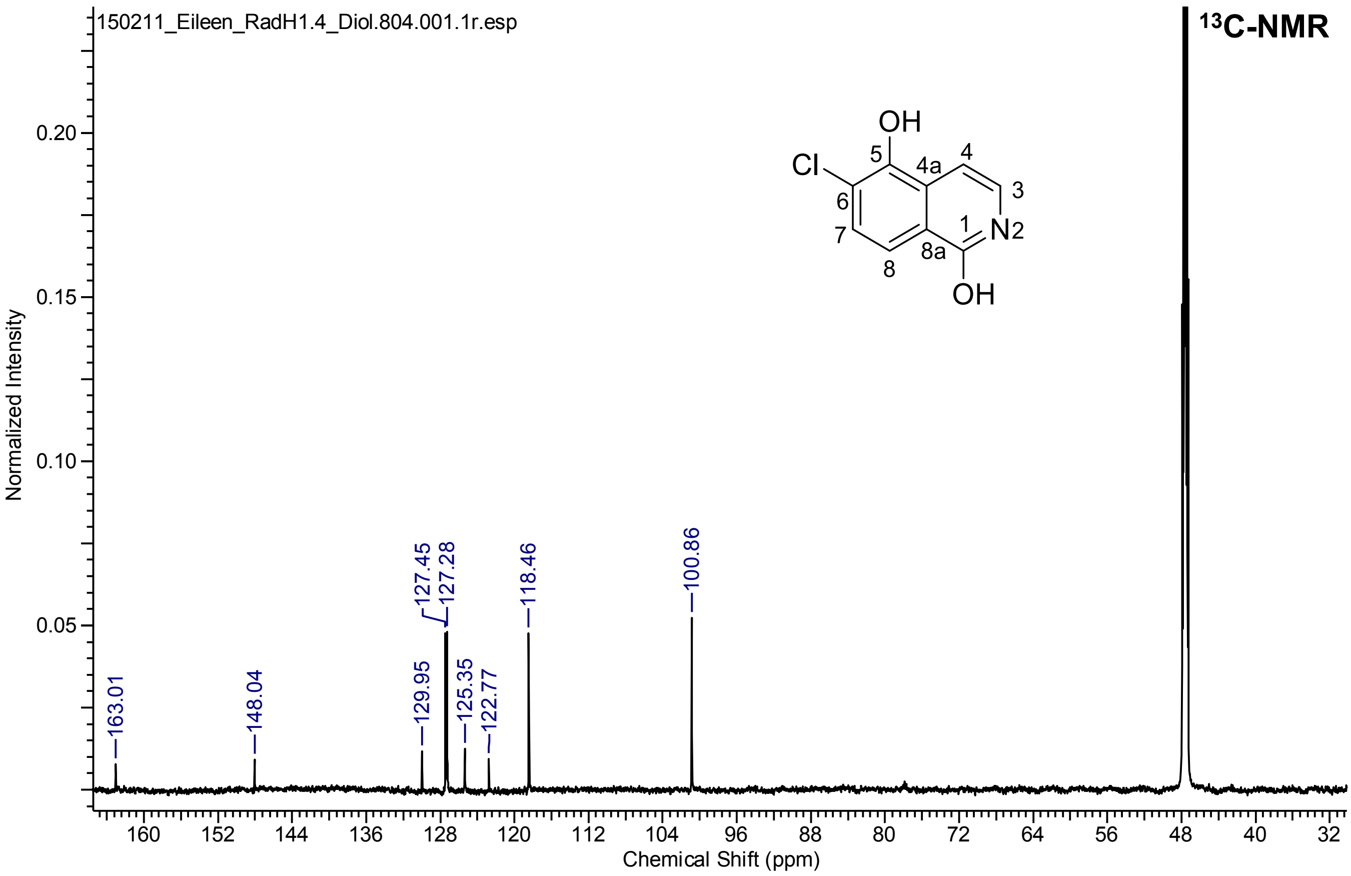

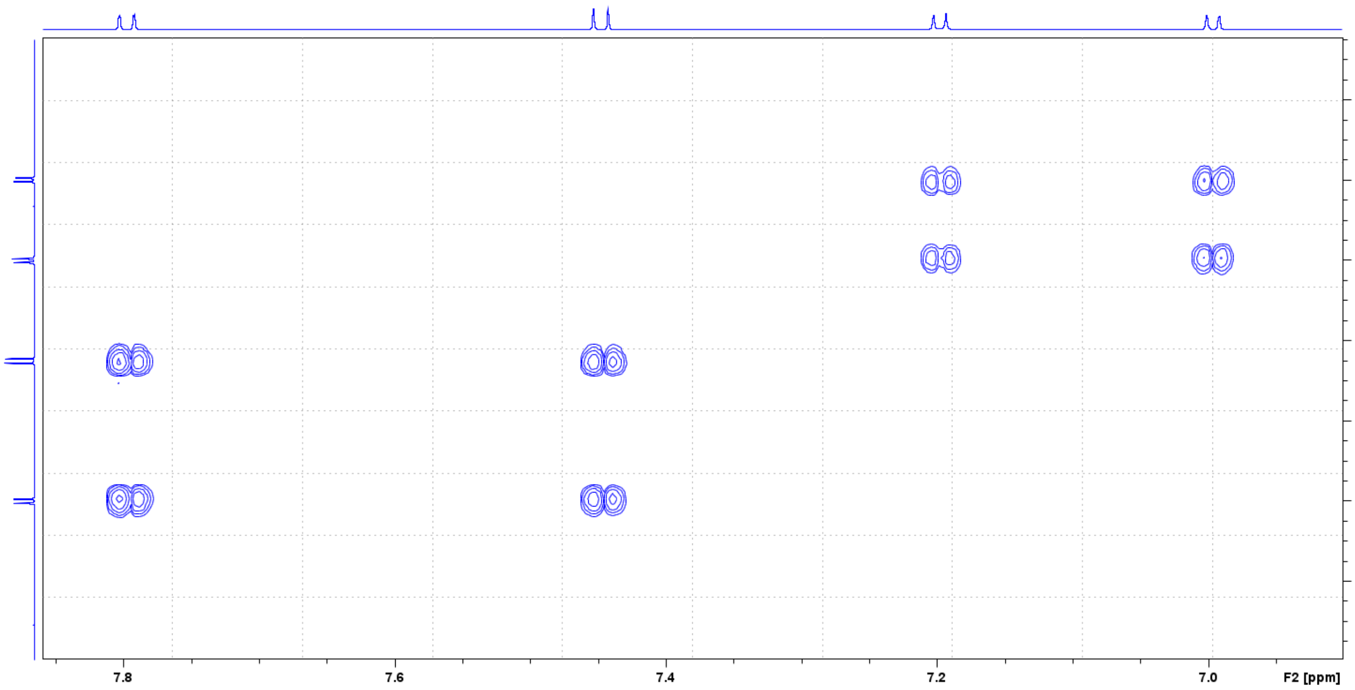

^{13}C NMR and COSY of 6-chloro-1,5-dihydroxyisoquinoline (**7a**).

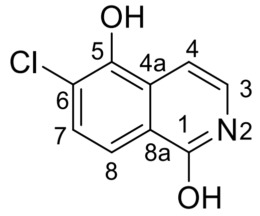

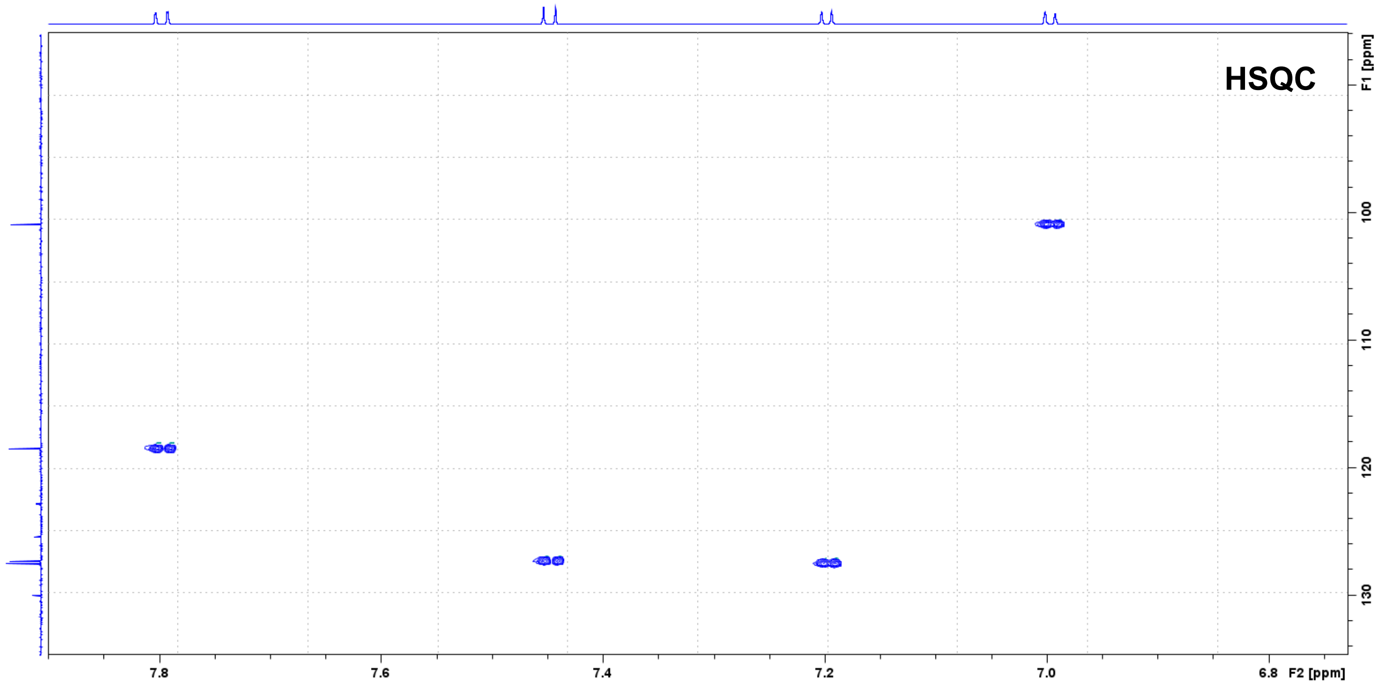

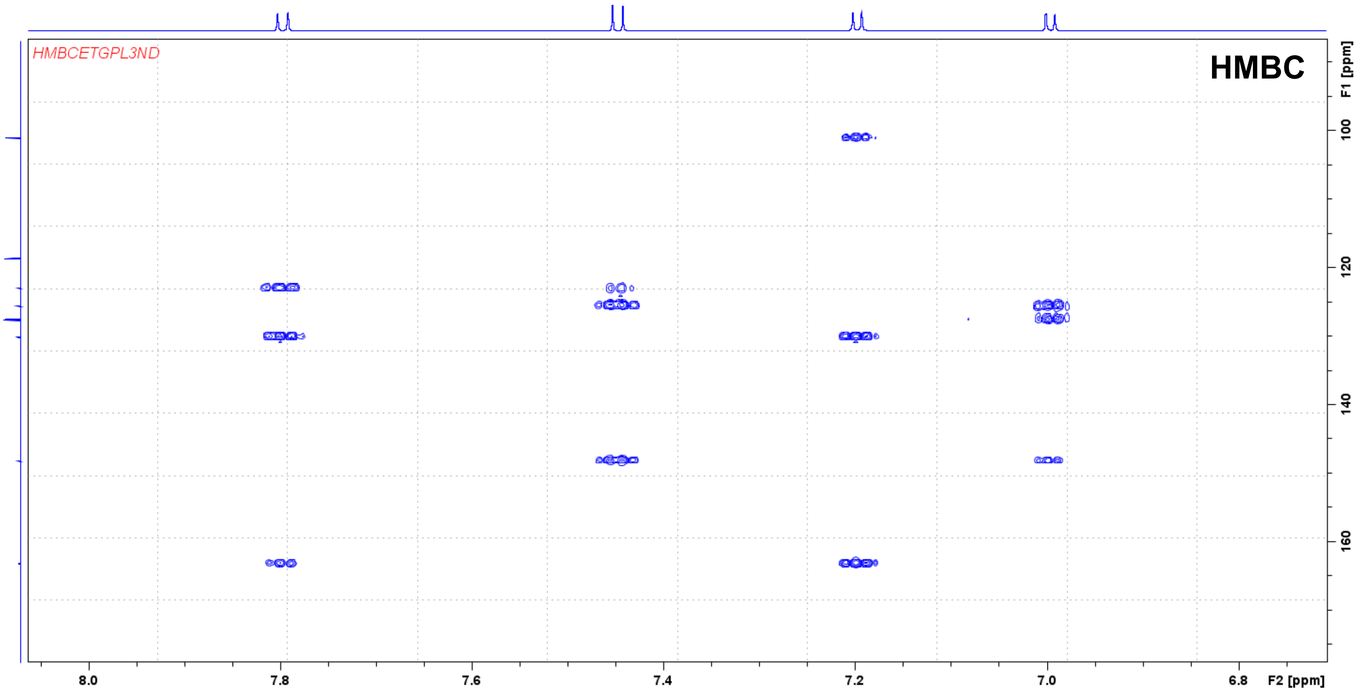

HSQC and HMBC of 6-chloro-1,5-dihydroxyisoquinoline (**7a**).

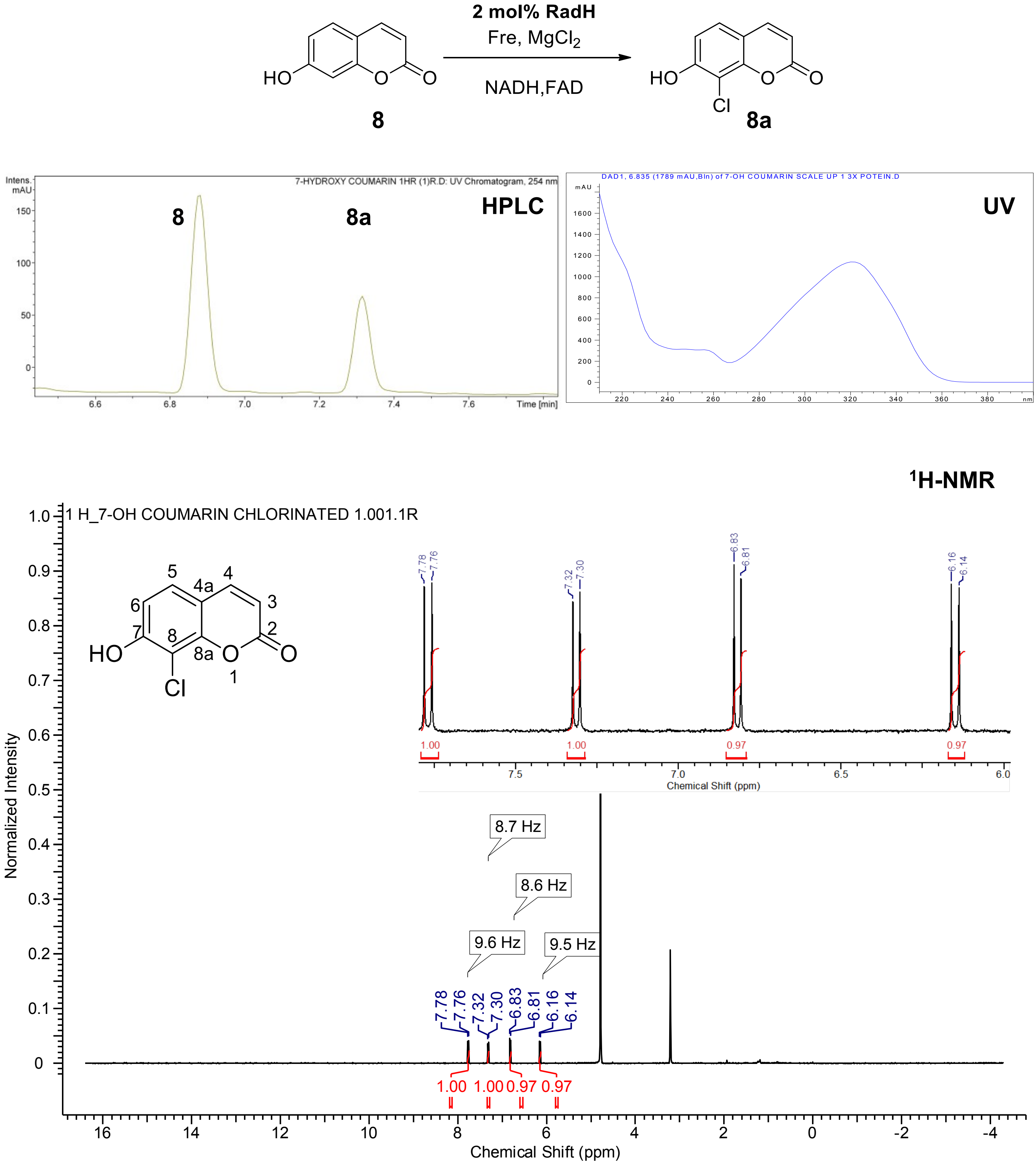

HPLC trace, UV spectrum and ^{1}H NMR of 8-chloro-7-hydroxycoumarin (**8a**).

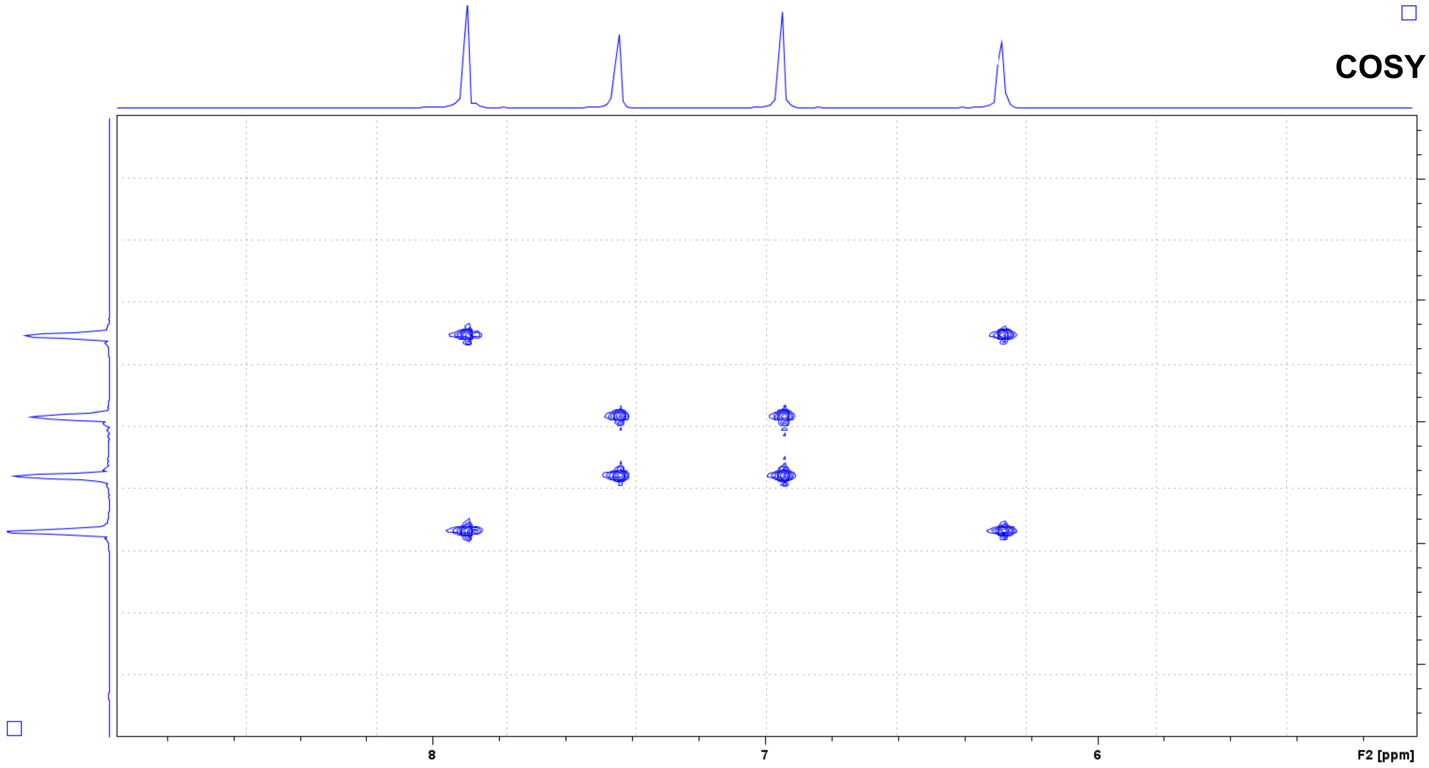

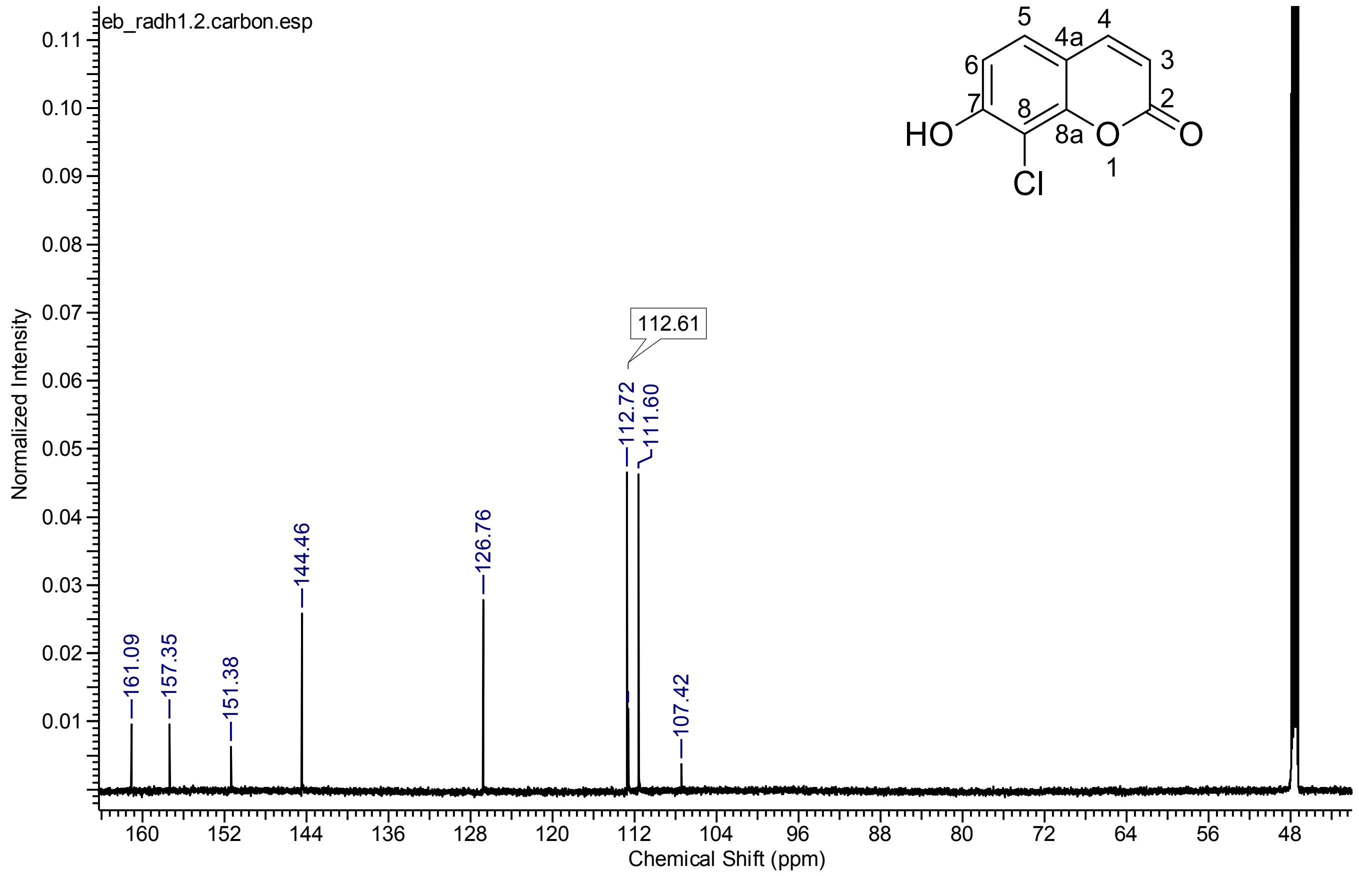

COSY and ^{13}C NMR of 8-chloro-7-hydroxycoumarin (**8a**).

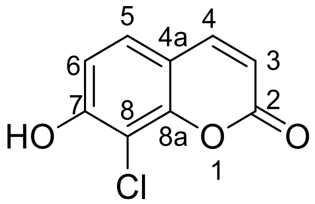

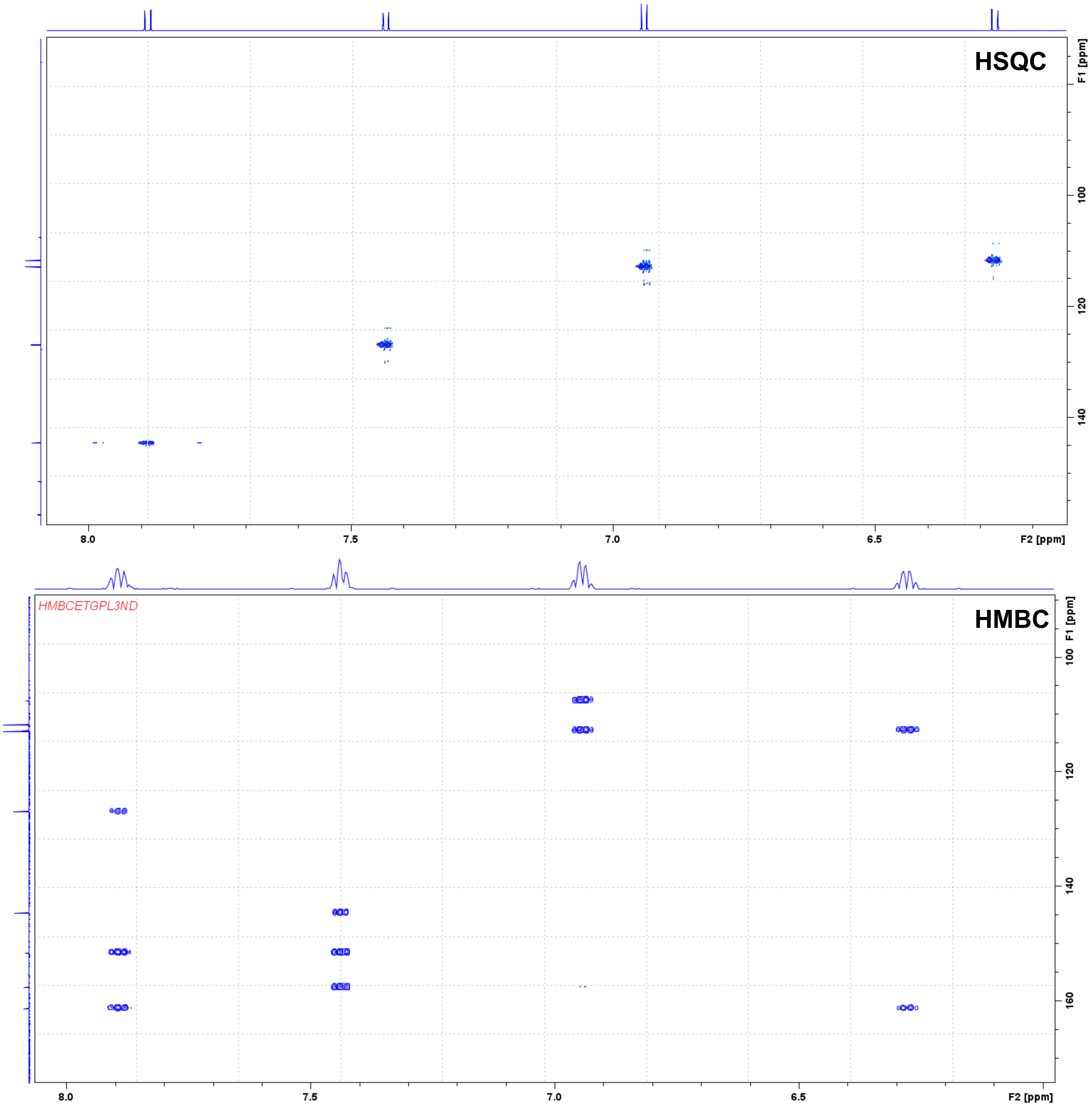

HSQC and HMBC of 8-chloro-7-hydroxycoumarin (**8a**).

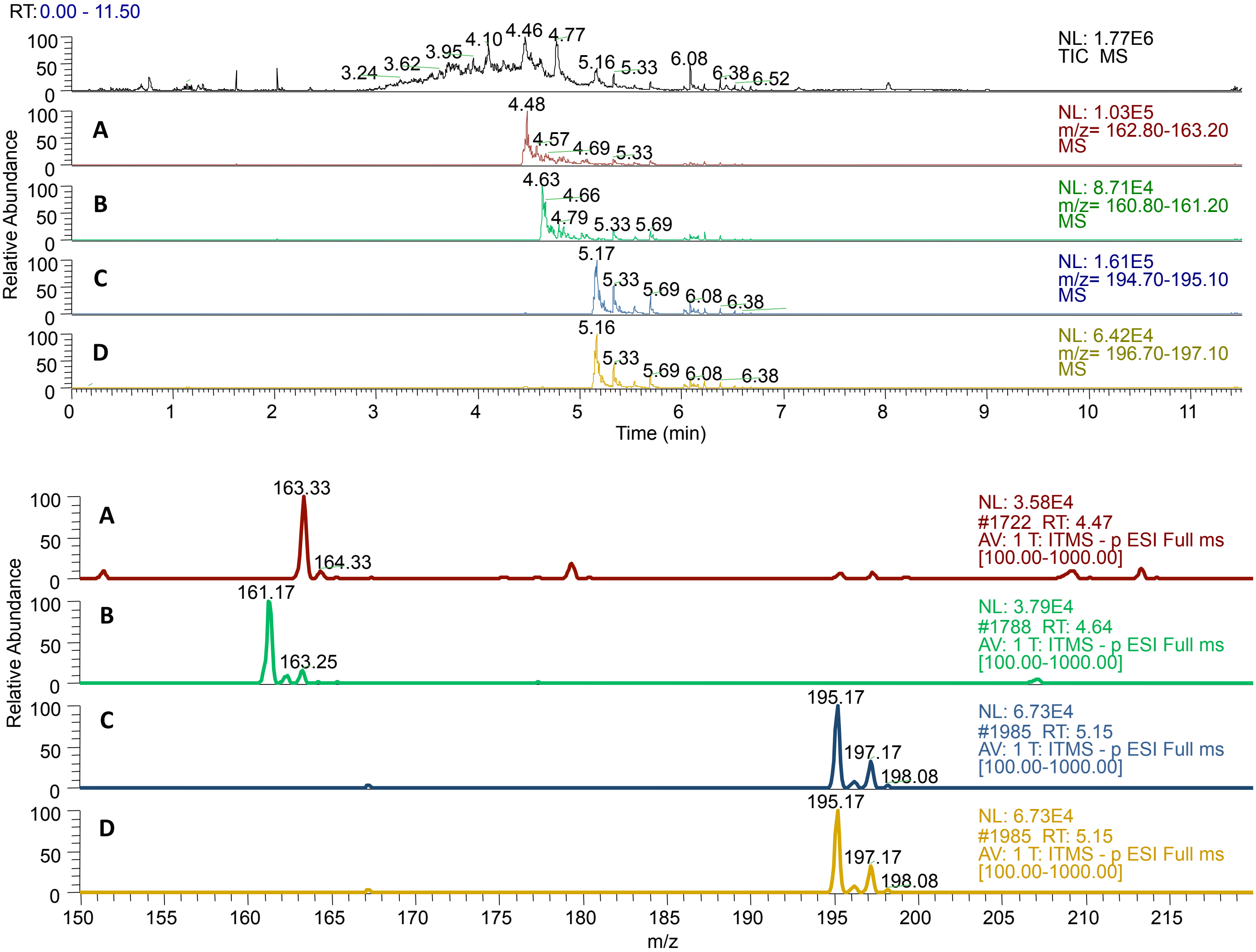

LC-MS analysis of the engineered pathway to 8-chloro-7-hydroxycoumarin production in *E. coli* from p-coumaric acid. Total ion chromatogram of *E. coli* cell culture extract is shown along with the extracted ion chromatograms for p-coumaric acid (**A**), 7-hydroxycoumarin (**B**) 8-chloro-7-hydroxycoumarin (**C** and **D**) and the corresponding MS. The *E. coli* cells contained plasmids that carry genes for 4-coumaryl-CoA ligase (4CL), feruloyl CoA 6′ hydroxylase (F6′H) from and radicicol halogenase (RadH double mutant).

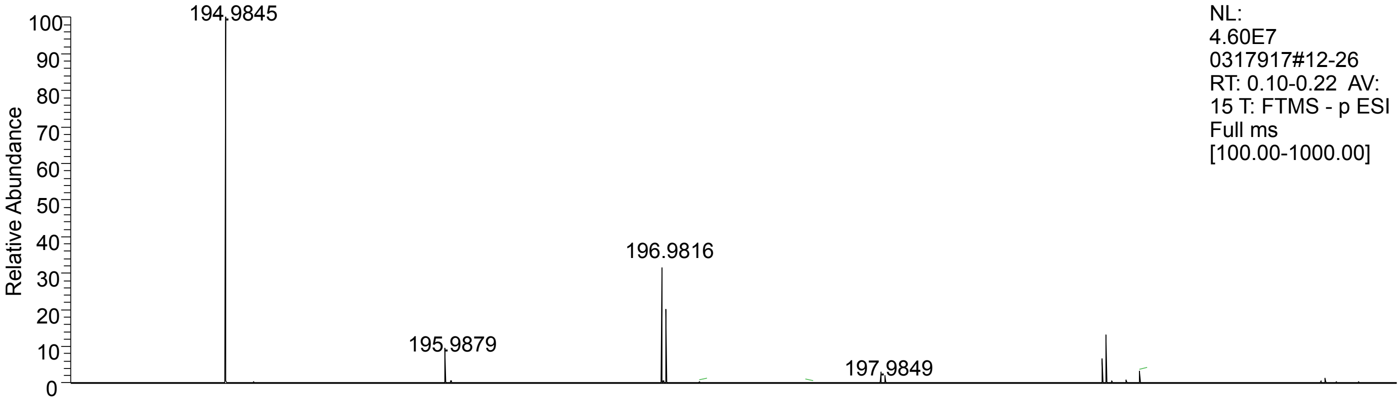

High resolution MS analysis of 8-chloro-7-hydroxycoumarin producted in *E. coli*. MS corresponding to 8-chloro-7-hydroxycoumarin: calculated m/z = 194.9854 and 196.9824, observed 194.9845 (- 4.6 ppm) and 196.9816 (-4.1 ppm).

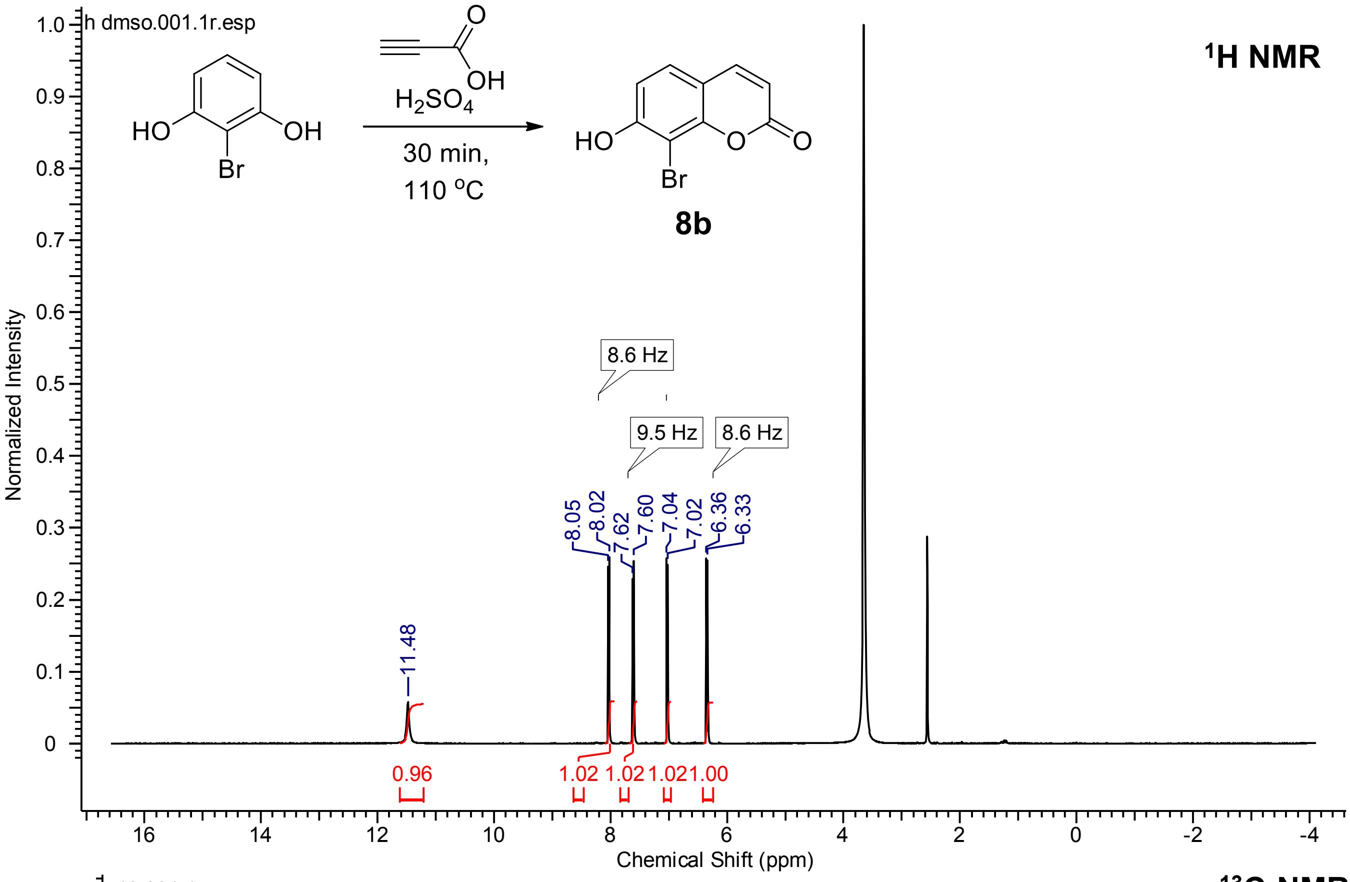

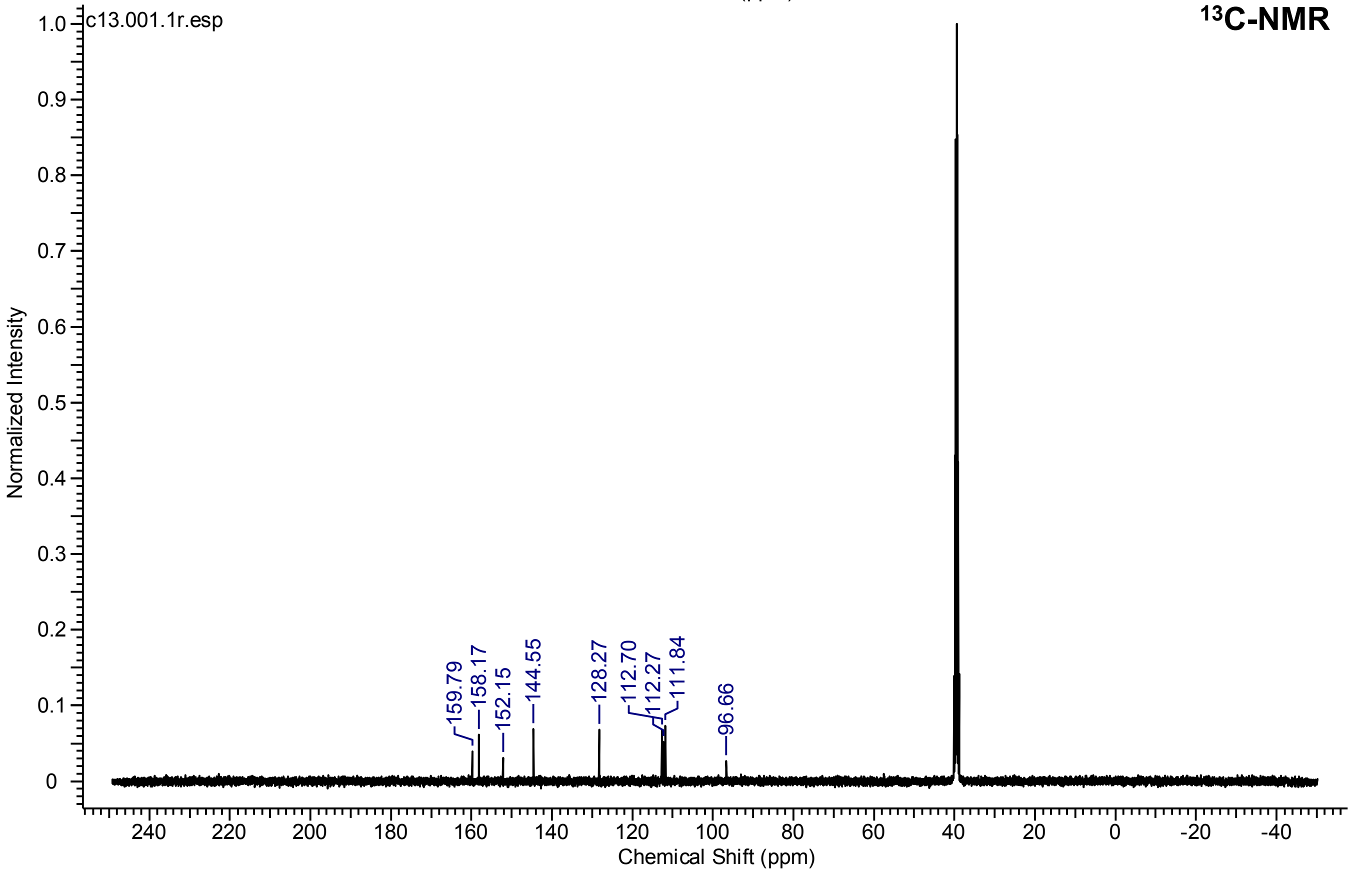

^{1}H NMR and ^{13}C NMR of 8-bromo-7-hydroxycoumarin (**8b**).

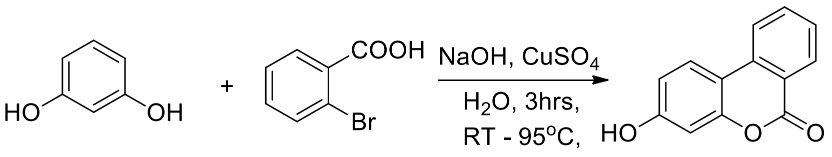

^{1}H-NMR

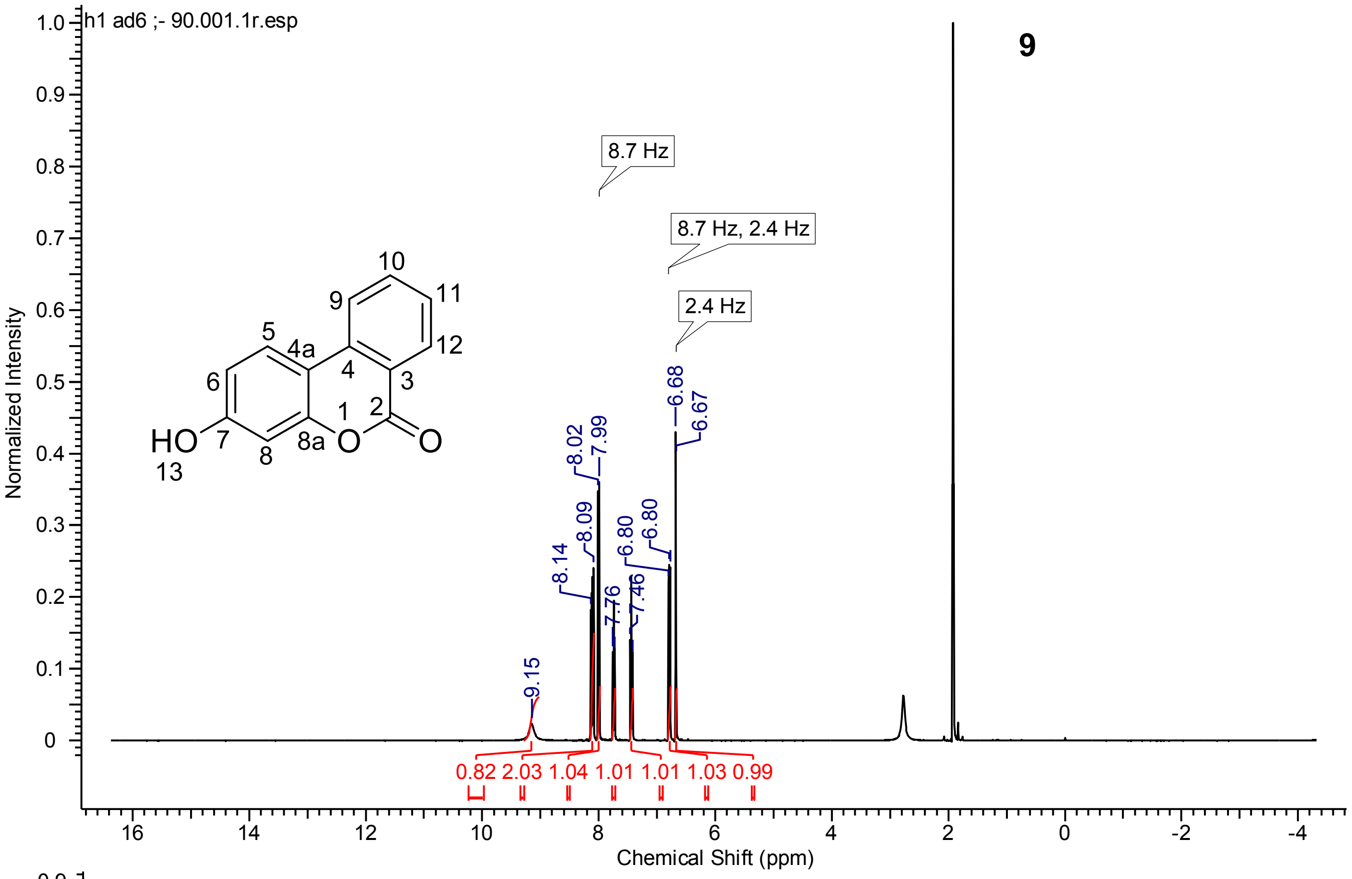

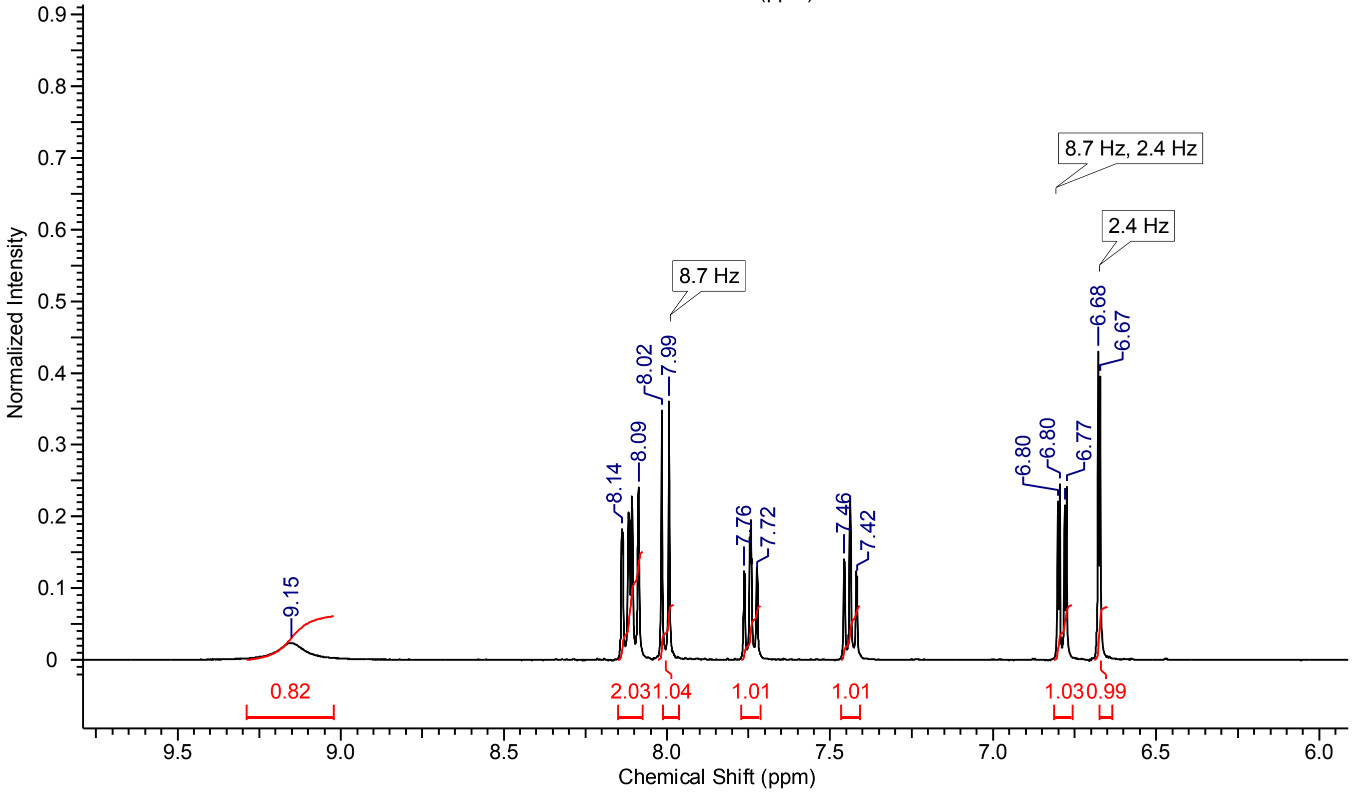

^{1}H NMR of 3-hydroxy-6H-benzo[c]chromen-6-one (**9**).

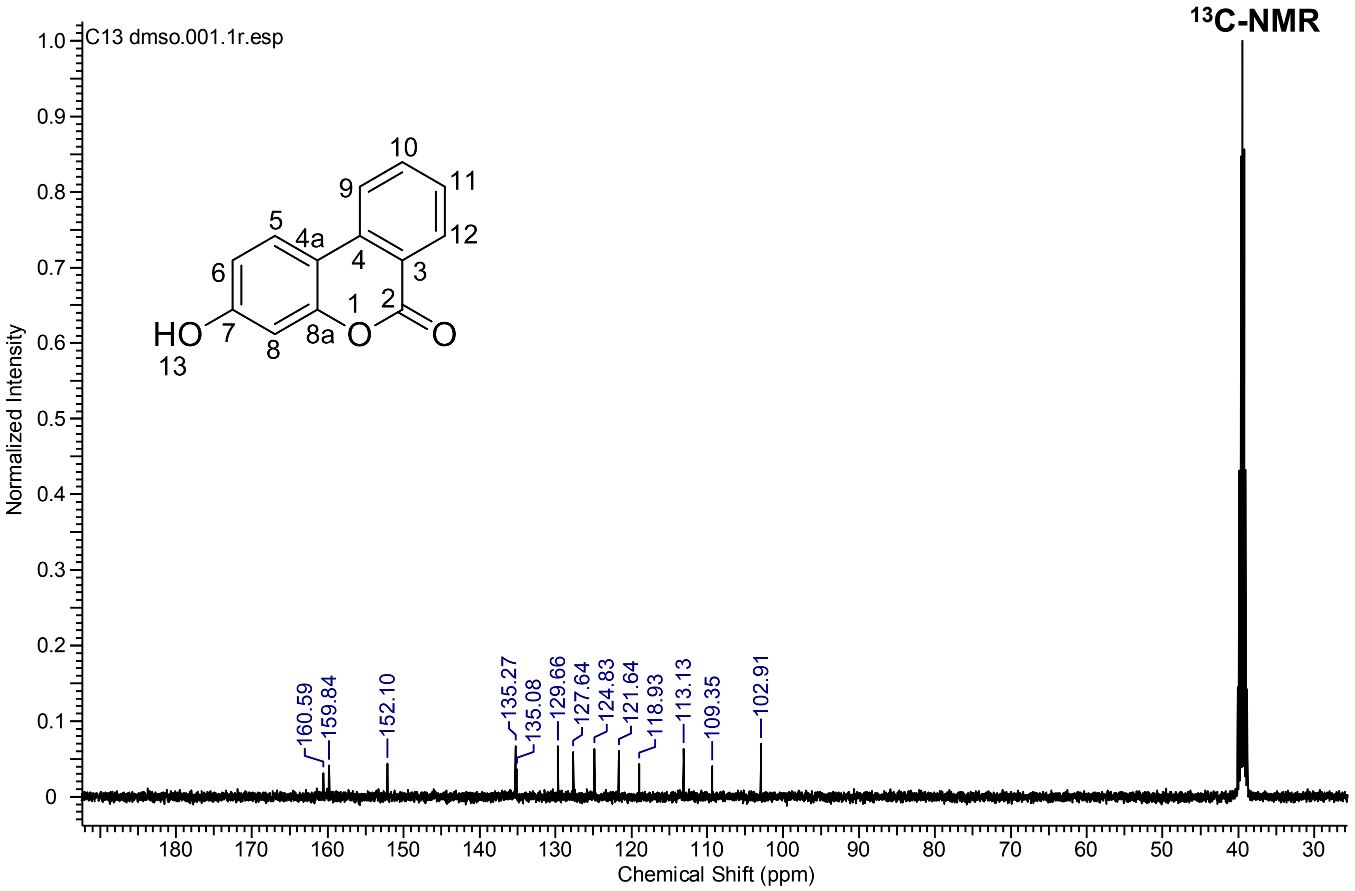

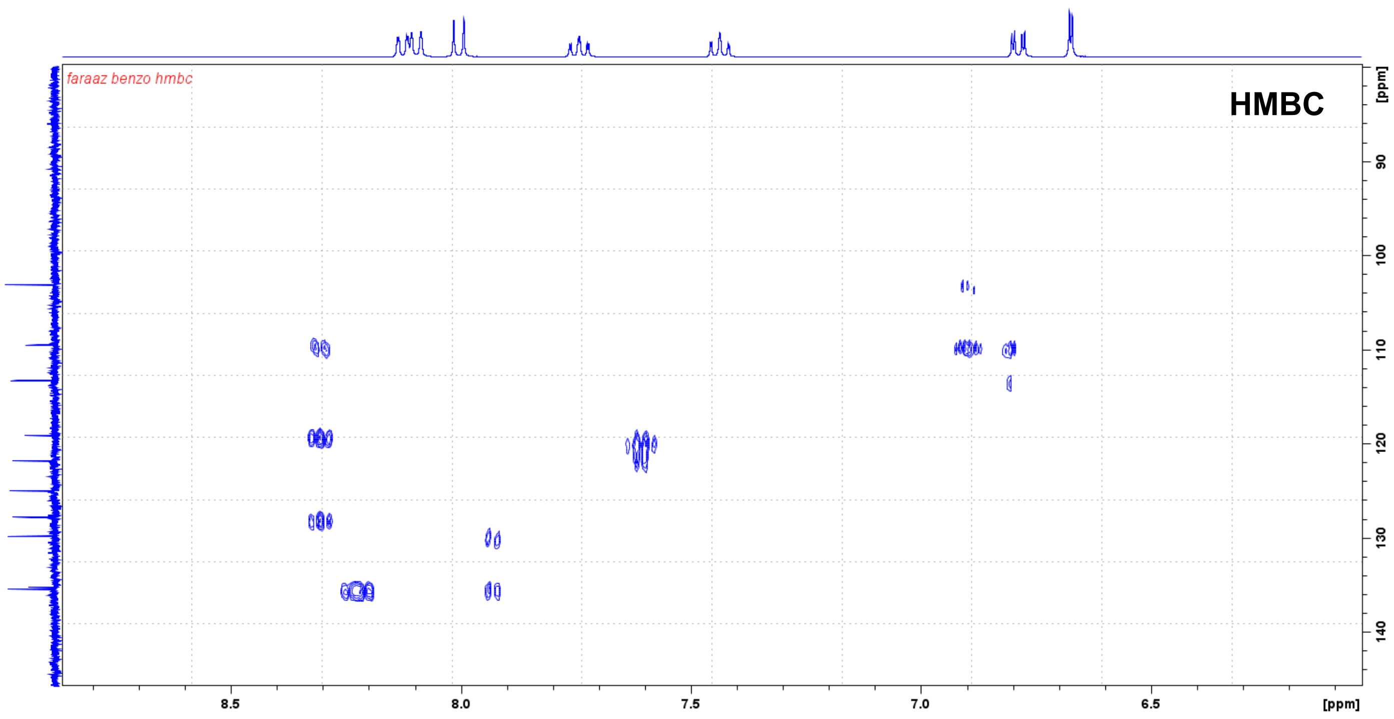

^{13}C NMR and HMBC of 3-hydroxy-6H-benzo[c]chromen-6-one (**9**).

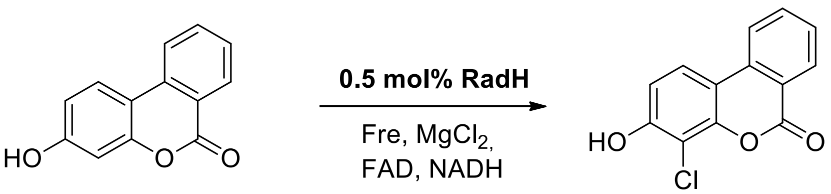

9 9a

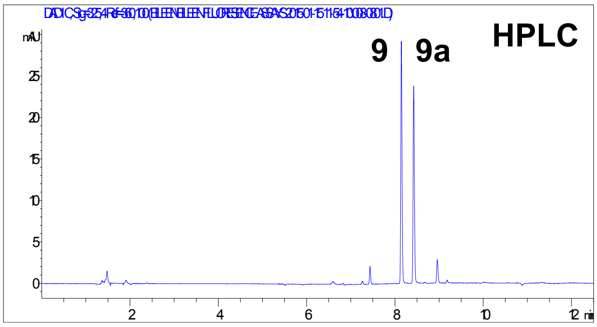

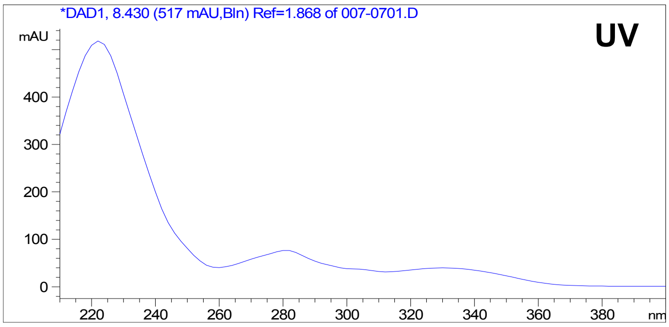

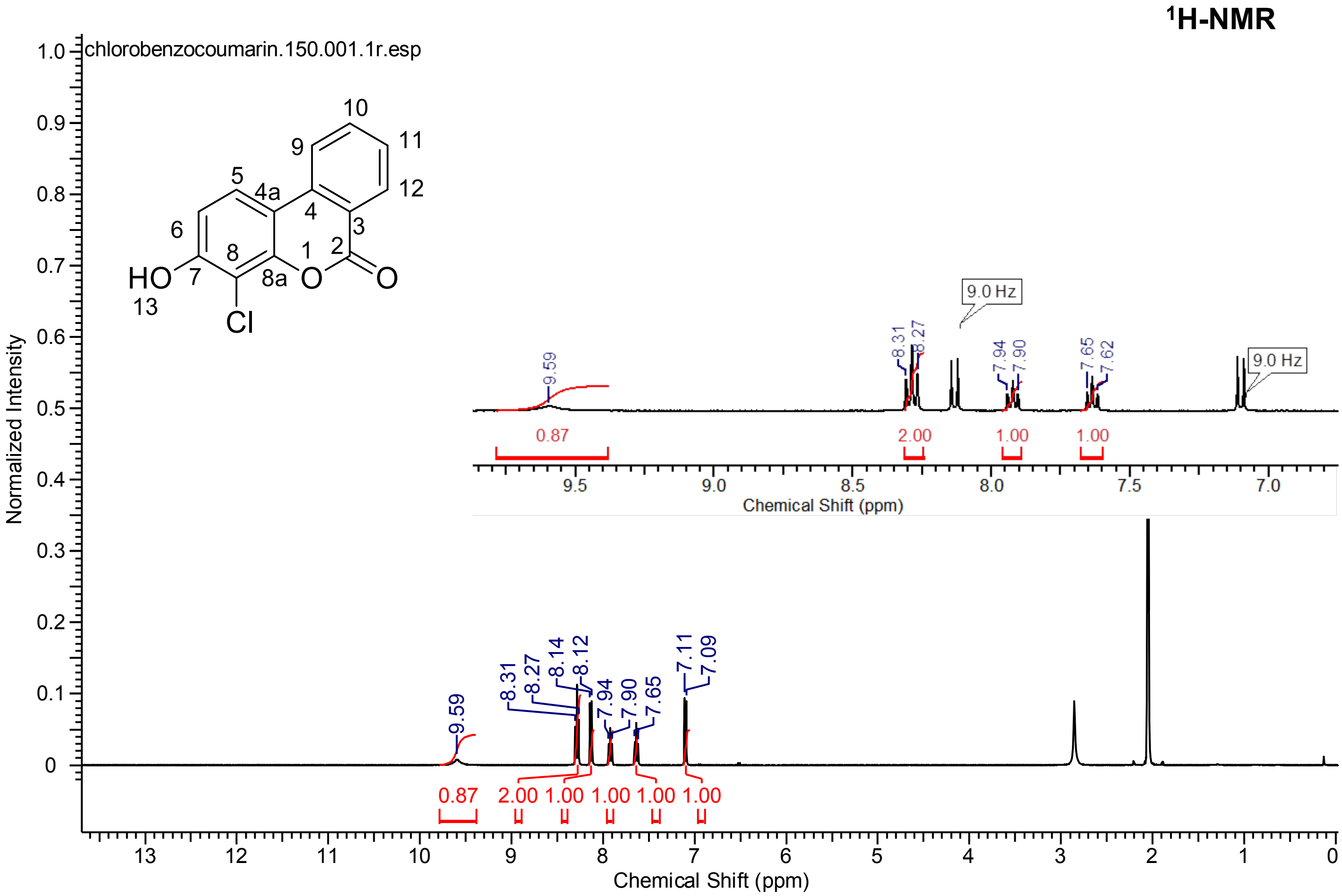

HPLC trace, UV spectrum and ^{1}H NMR of 8-chloro-7-hydroxy-3,4-benzocoumarin (**9a**).

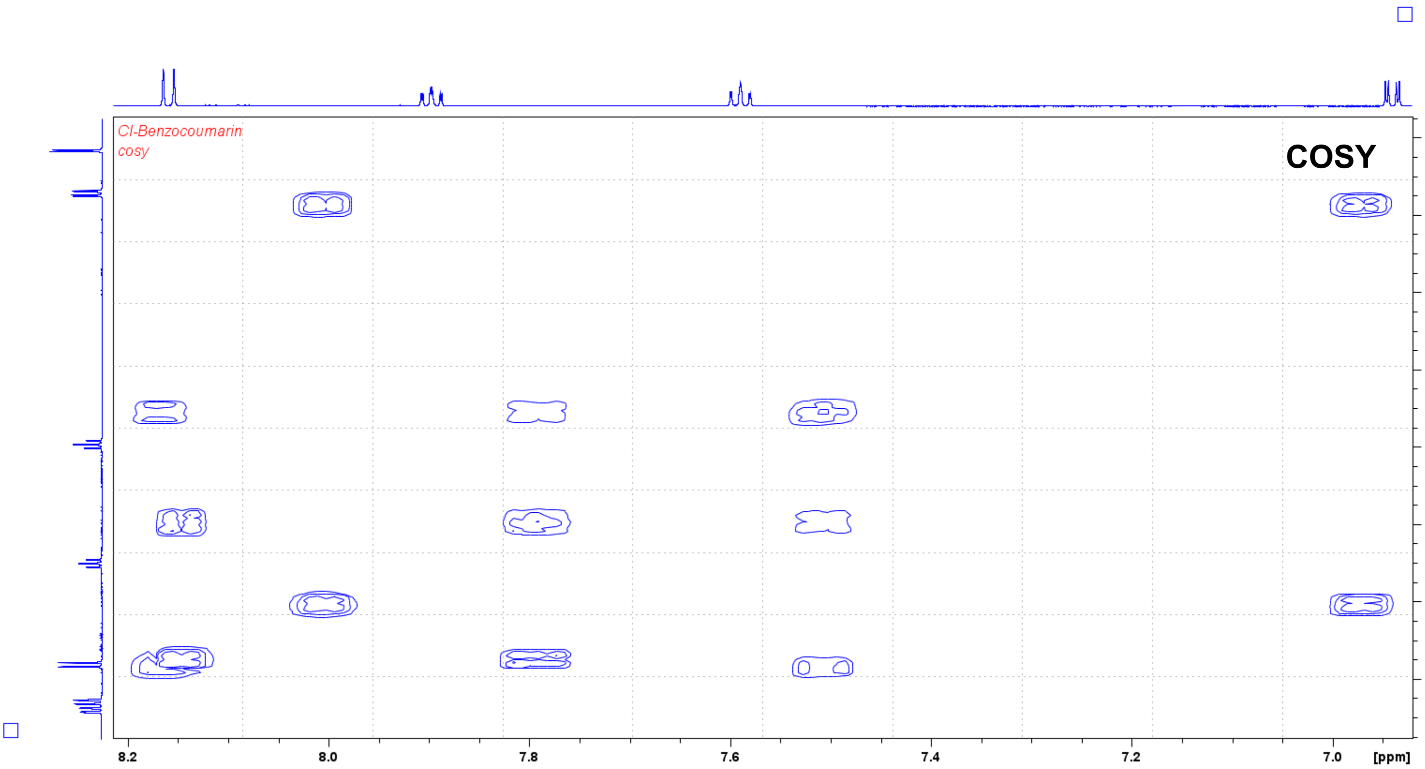

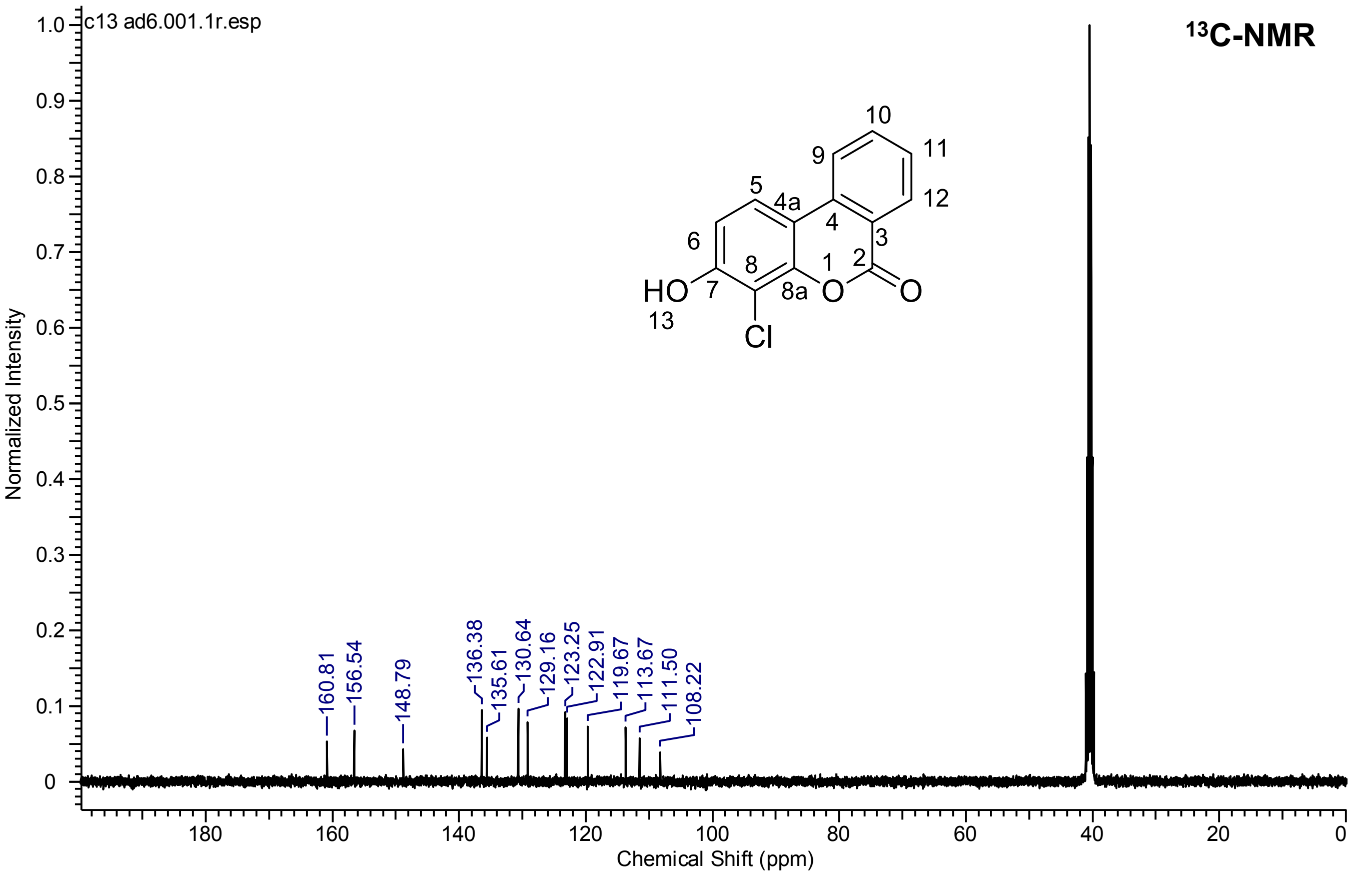

COSY and ^{13}C NMR of 8-chloro-7-hydroxy-3,4-benzocoumarin (**9a**).

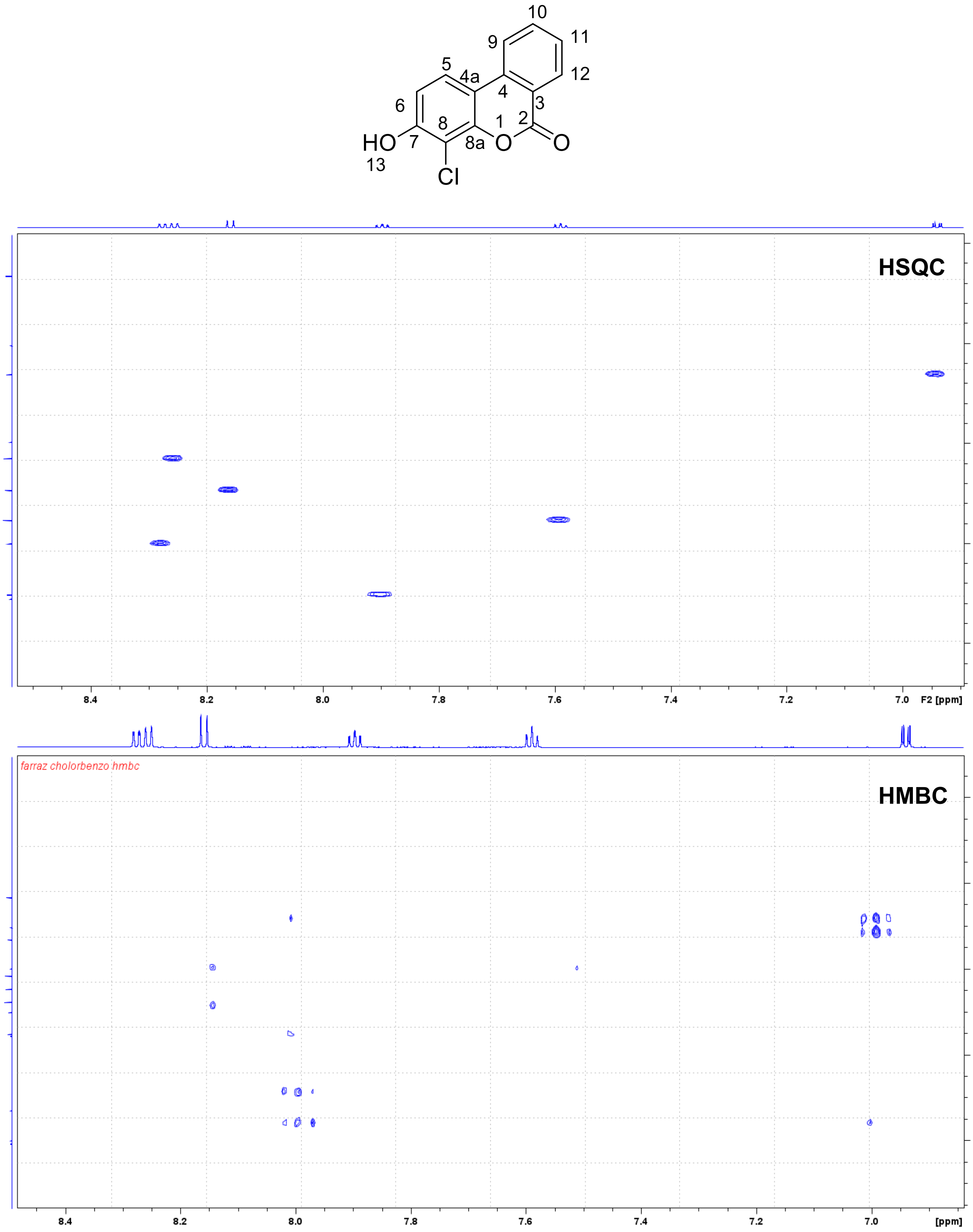

HSQC and HMBC of 8-chloro-7-hydroxy-3,4-benzocoumarin (**9a**).

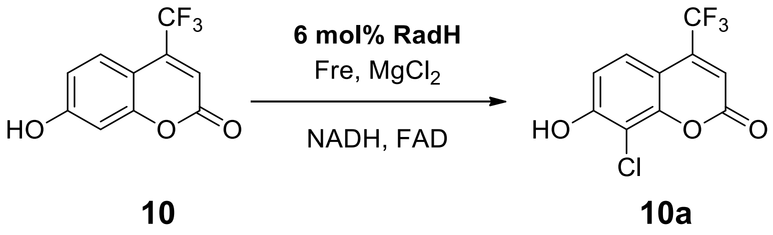

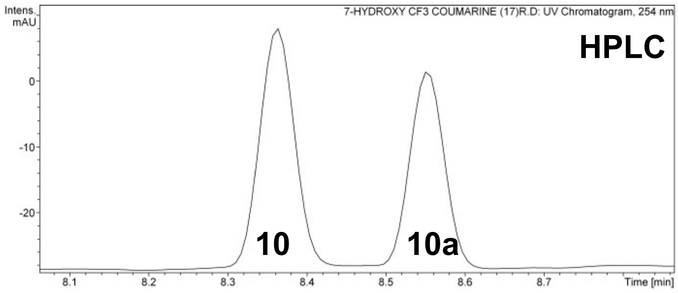

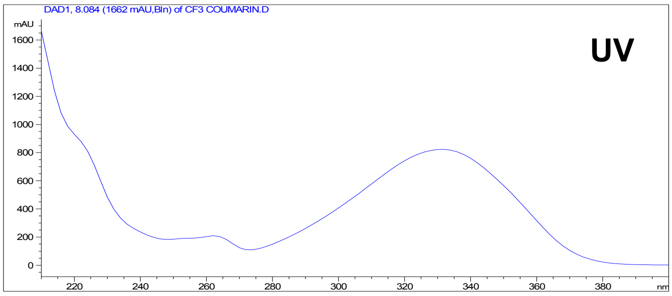

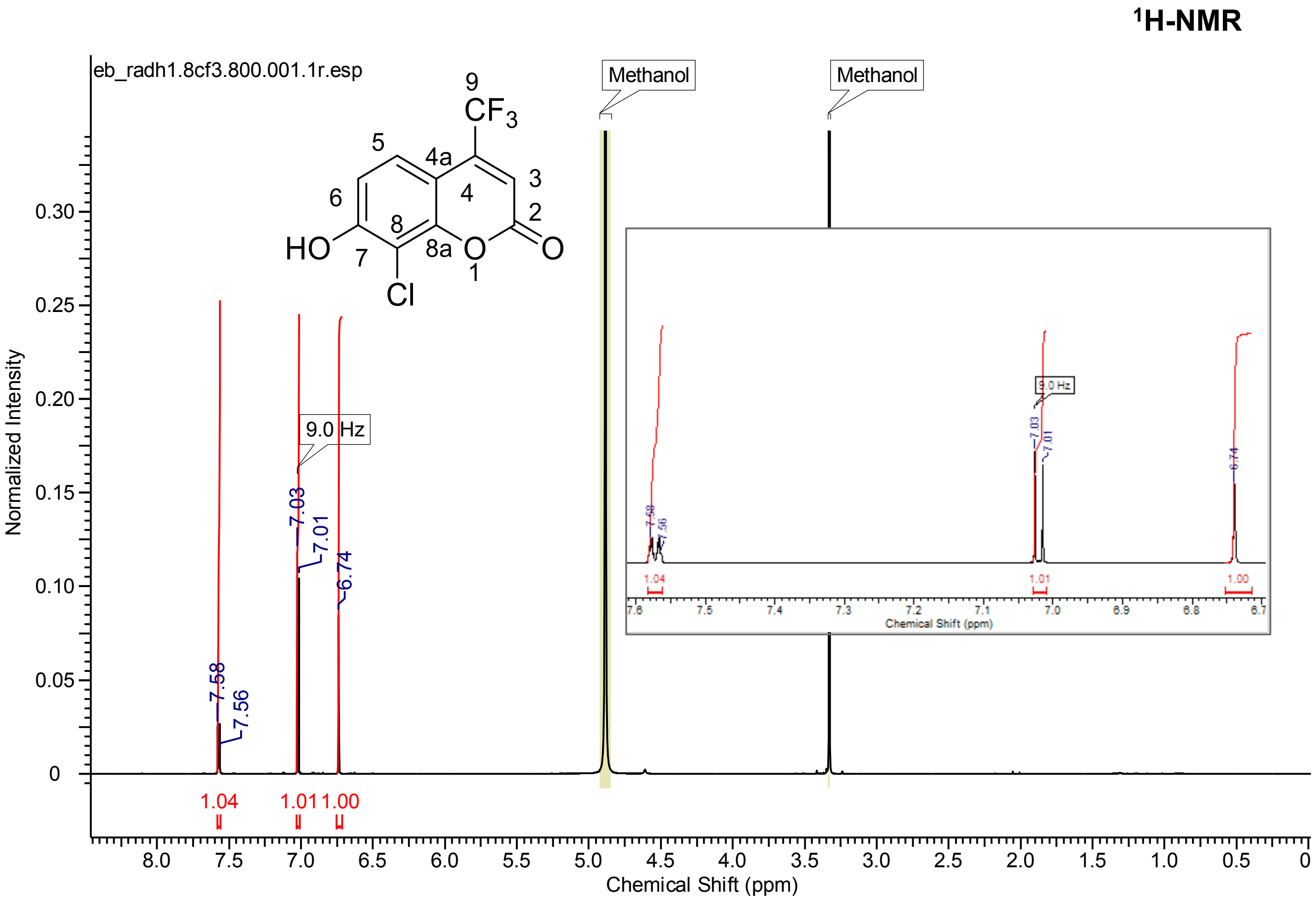

HPLC trace, UV spectrum and ^{1}H NMR of 8-chloro-7-hydroxy-4-(trifluoromethyl)-coumarin (**10a**).

COSY

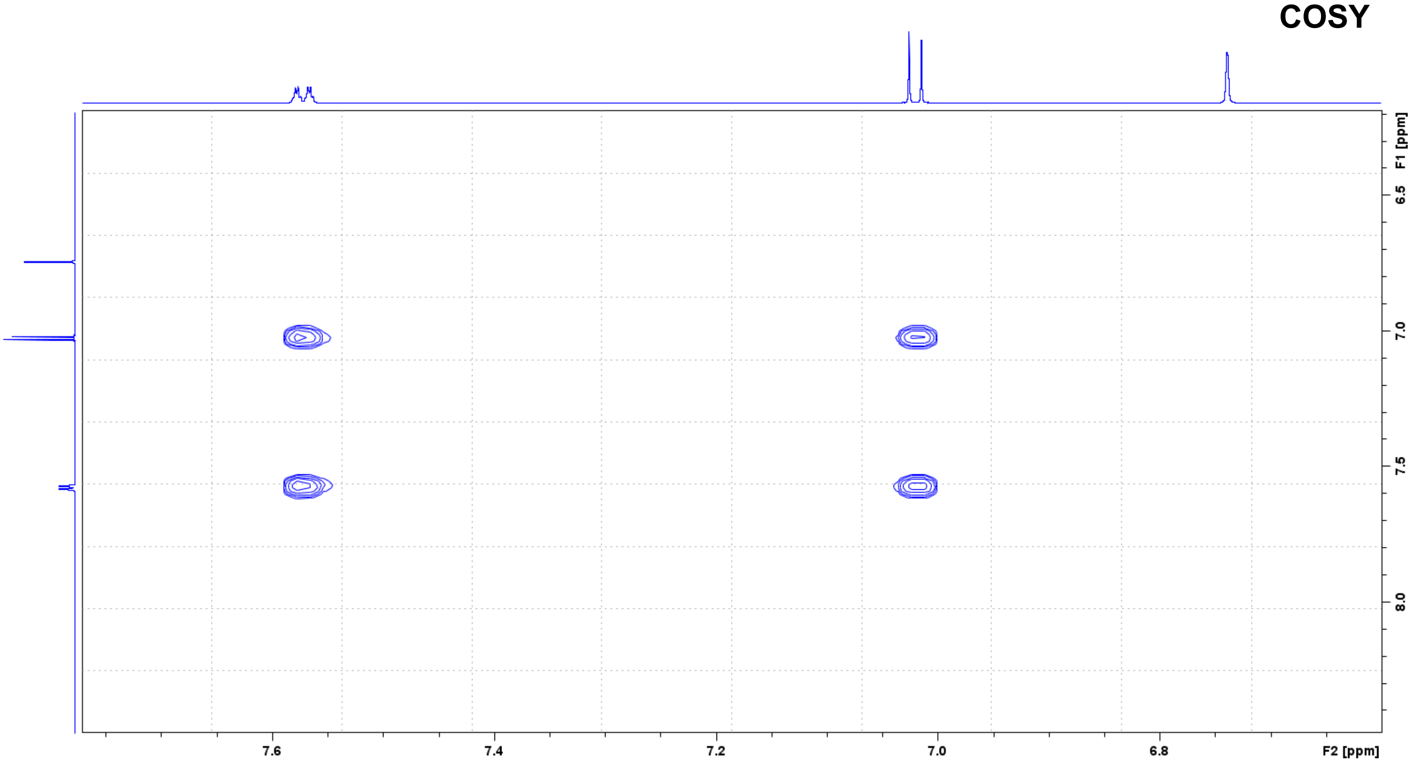

^{13}C-NMR

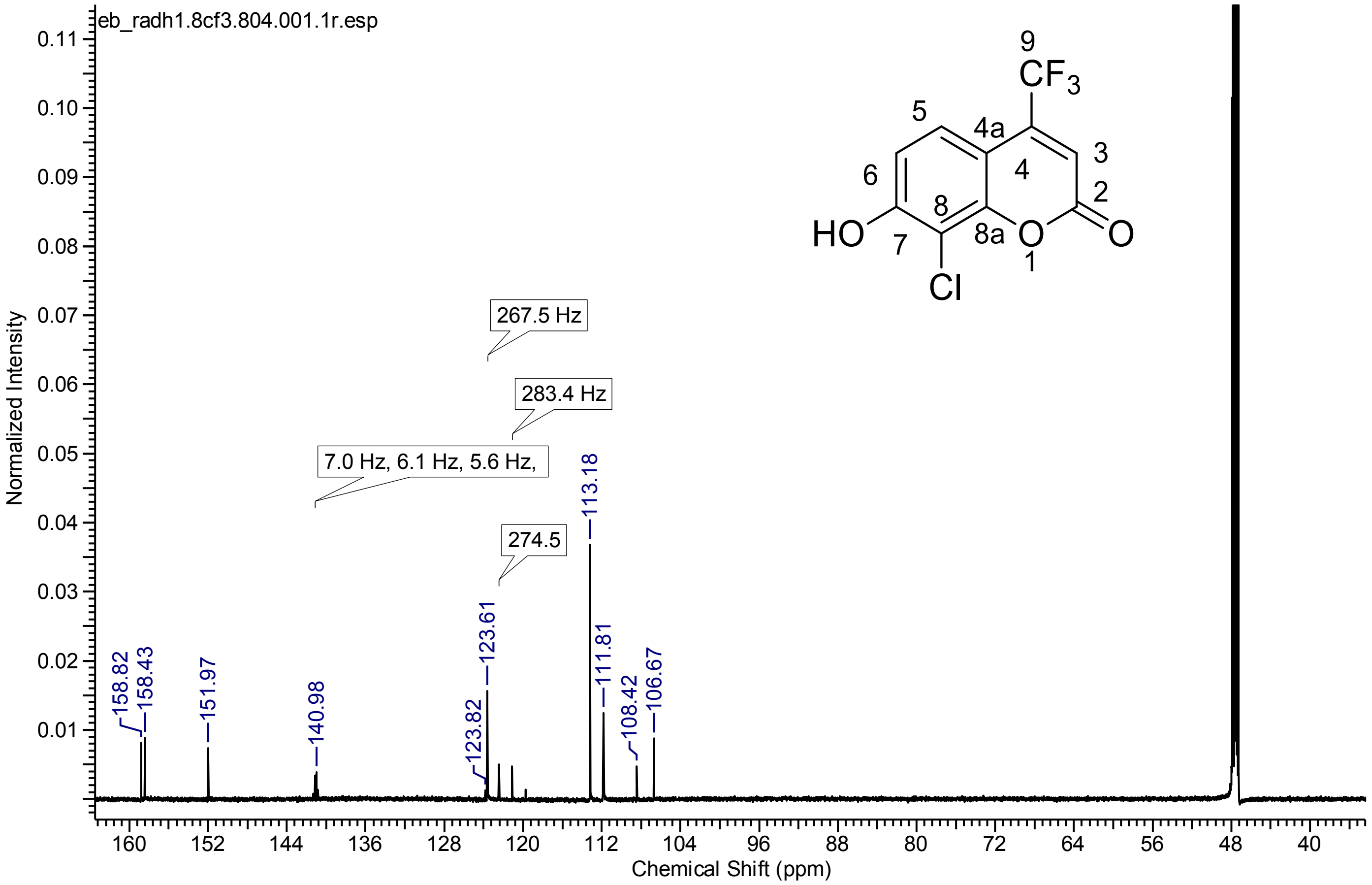

COSY and ^{13}C NMR of 8-chloro-7-hydroxy-4-(trifluoromethyl)-coumarin (**10a**).

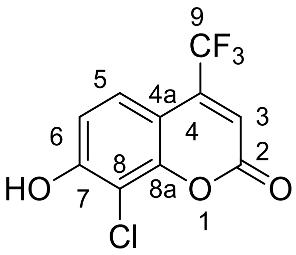

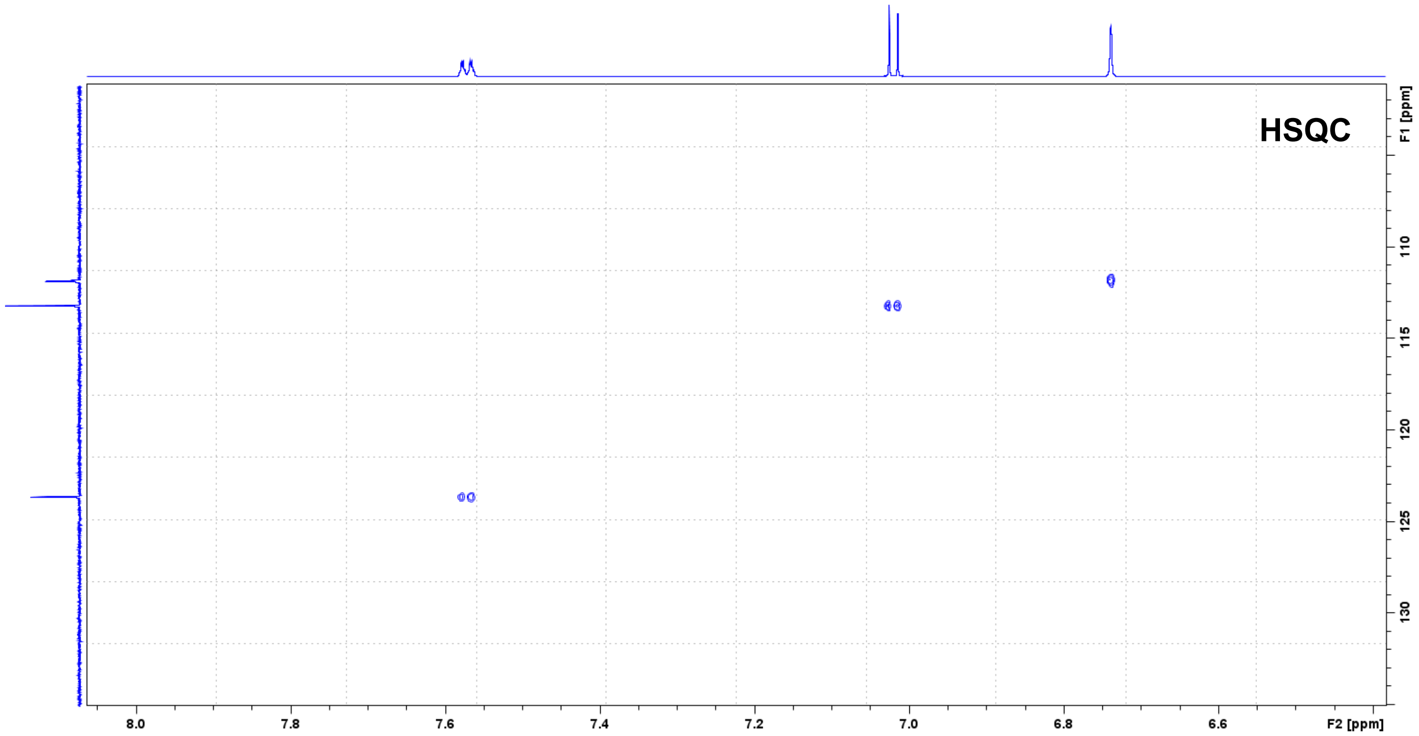

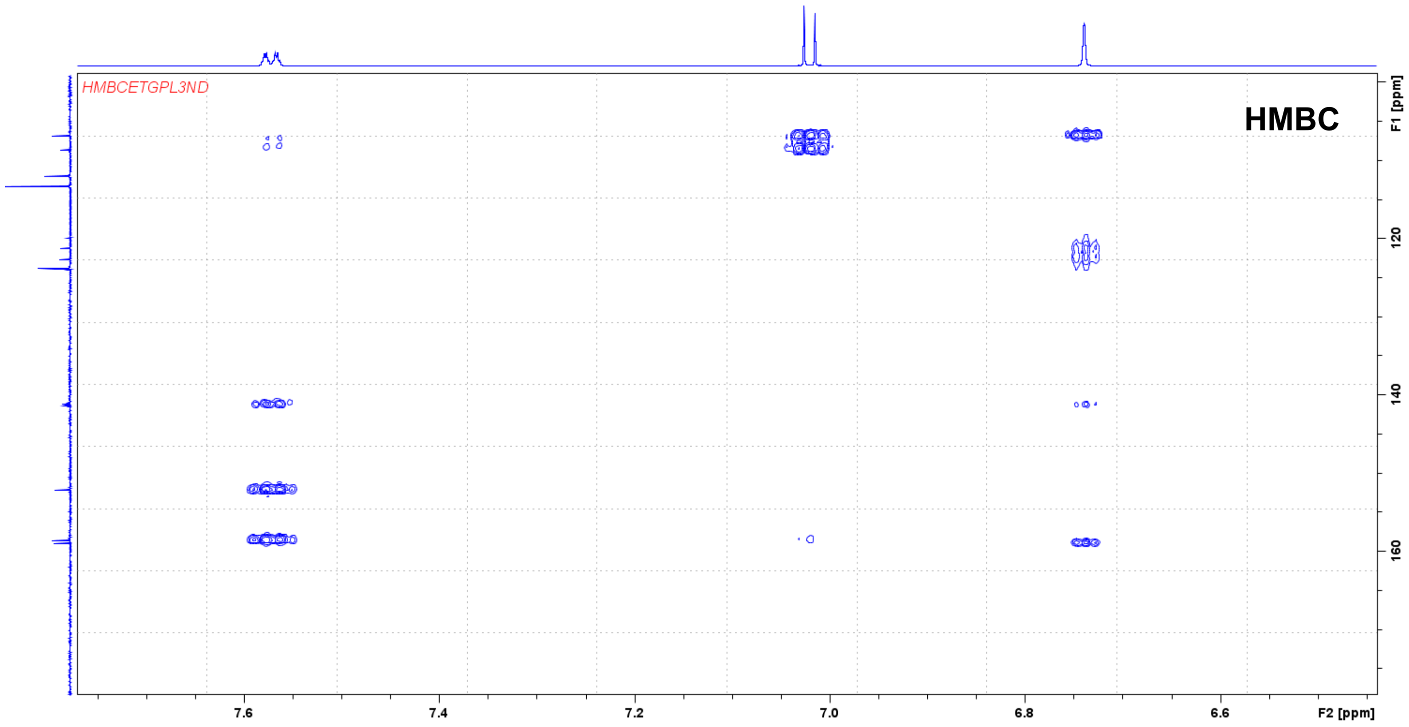

HSQC and HMBC of 8-chloro-7-hydroxy-4-(trifluoromethyl)-coumarin (**10a**).

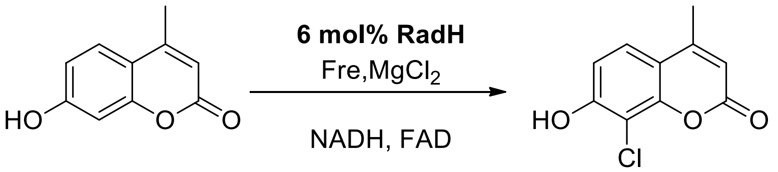

11 **11a**

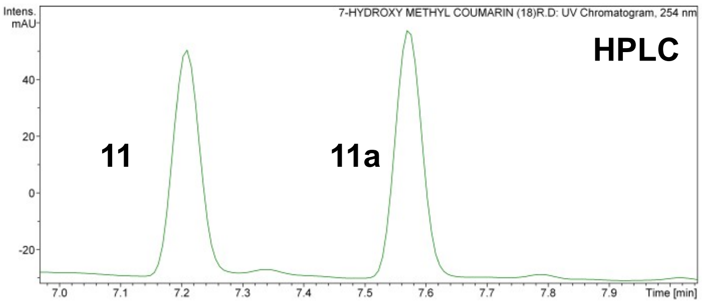

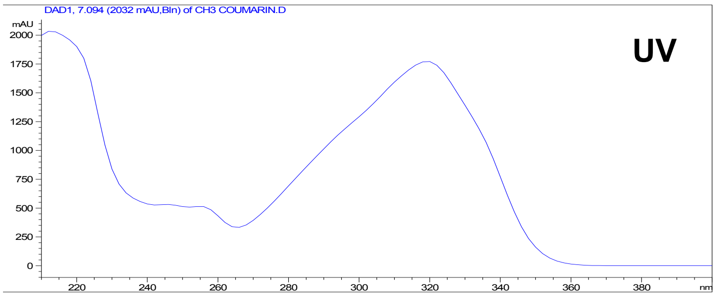

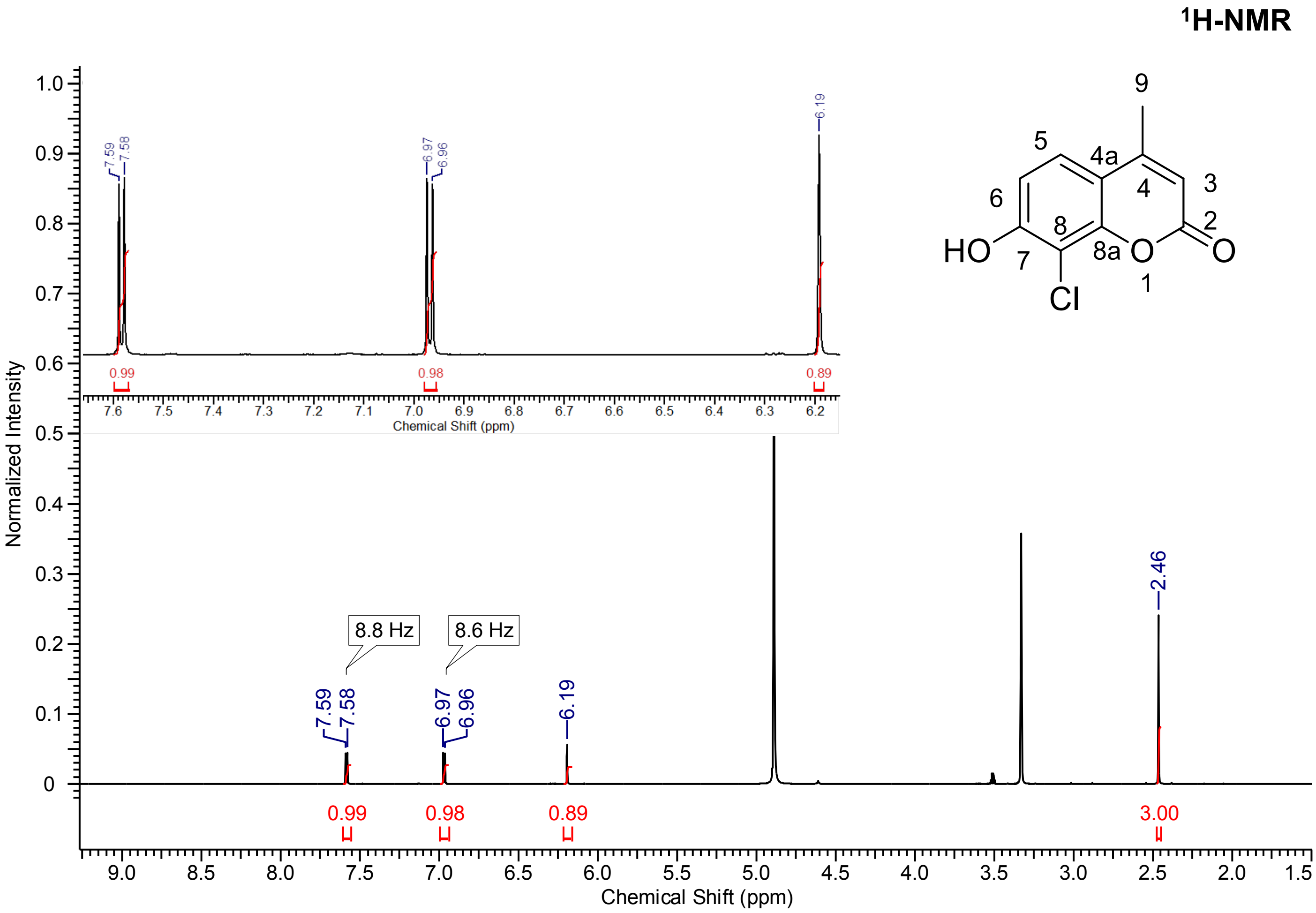

HPLC trace, UV spectrum and ^{1}H NMR of 8-chloro-7-hydroxy-4- methylcoumarin (**11a**).

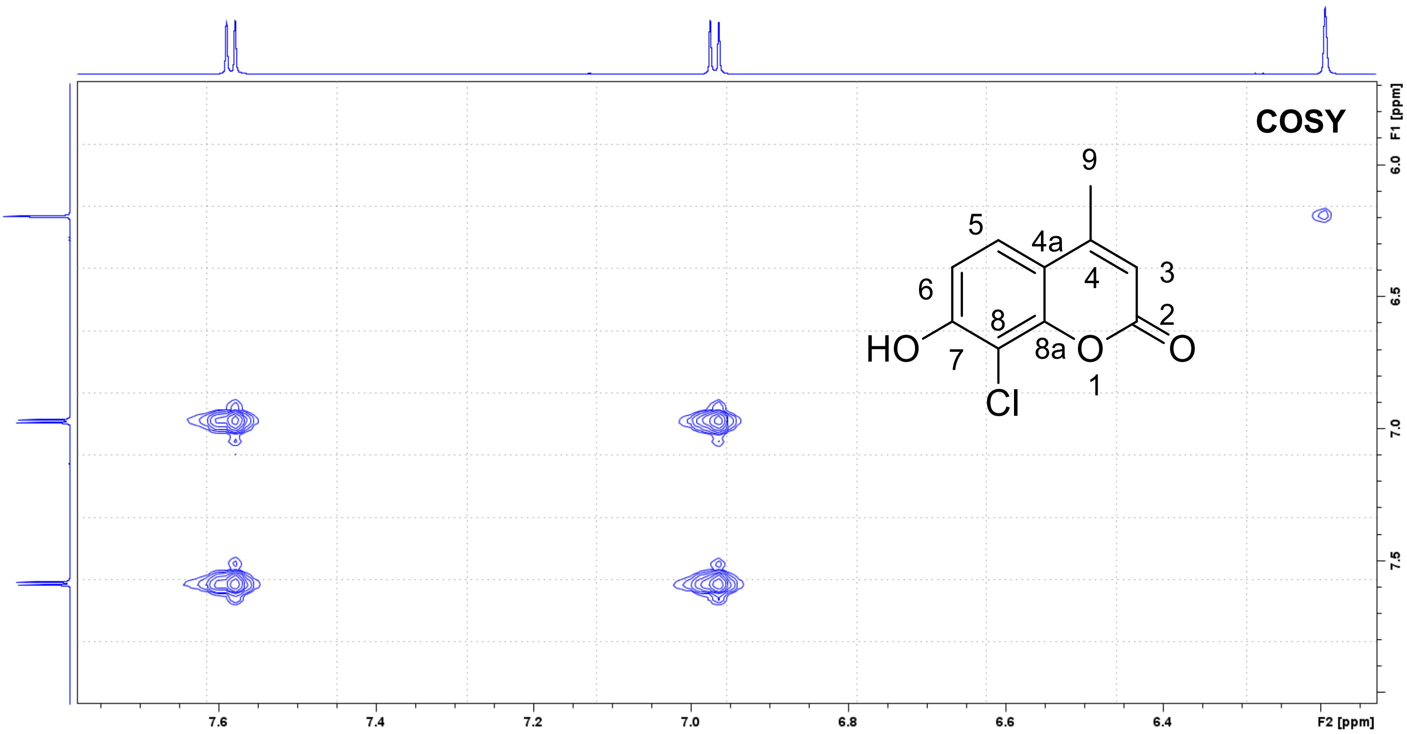

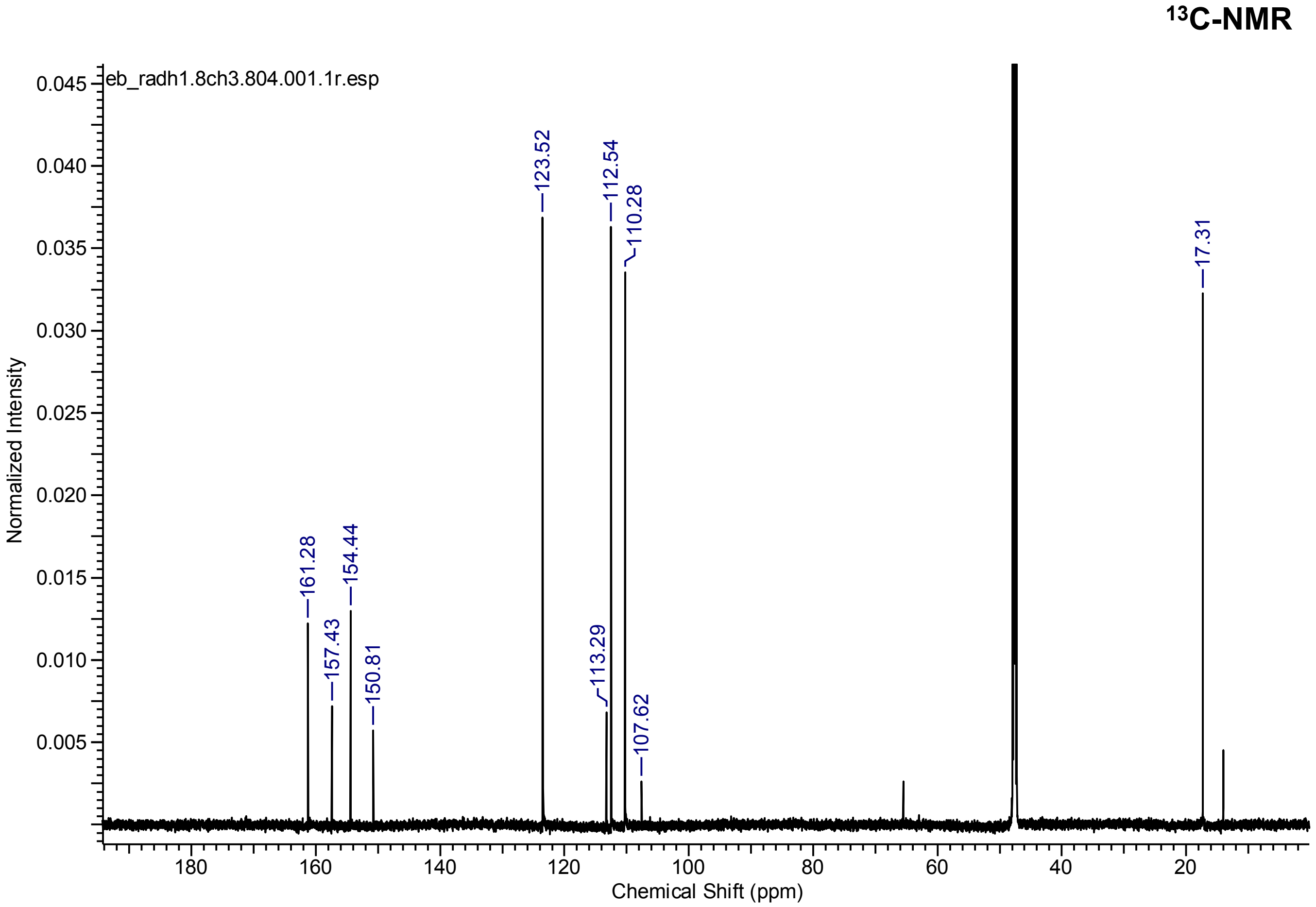

COSY and ^{13}C NMR of 8-chloro-7-hydroxy-4-methylcoumarin (**11a**).

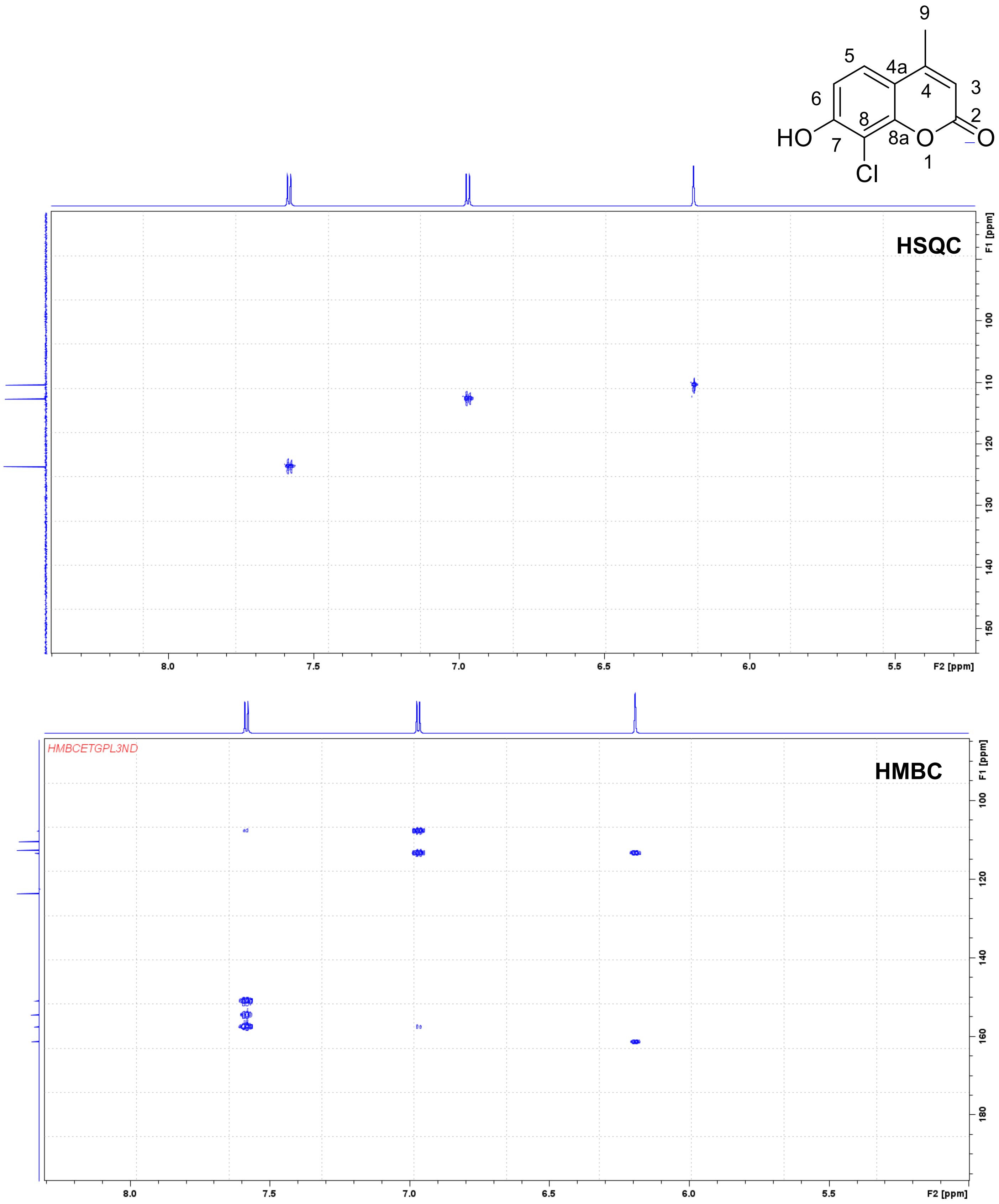

HSQC and HMBC of 8-chloro-7-hydroxy-4-methylcoumarin (**11a**).

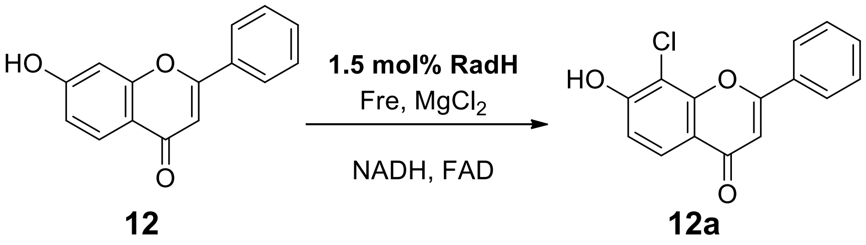

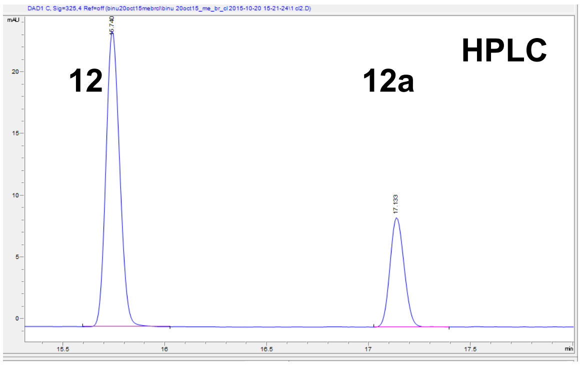

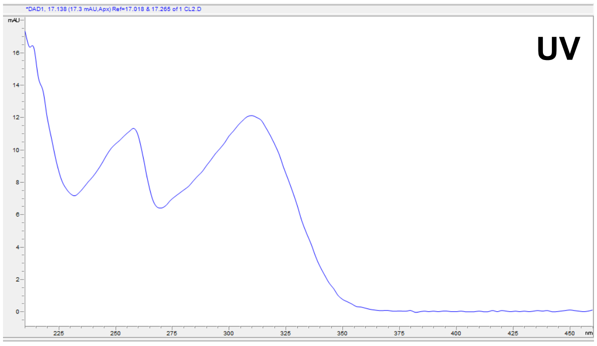

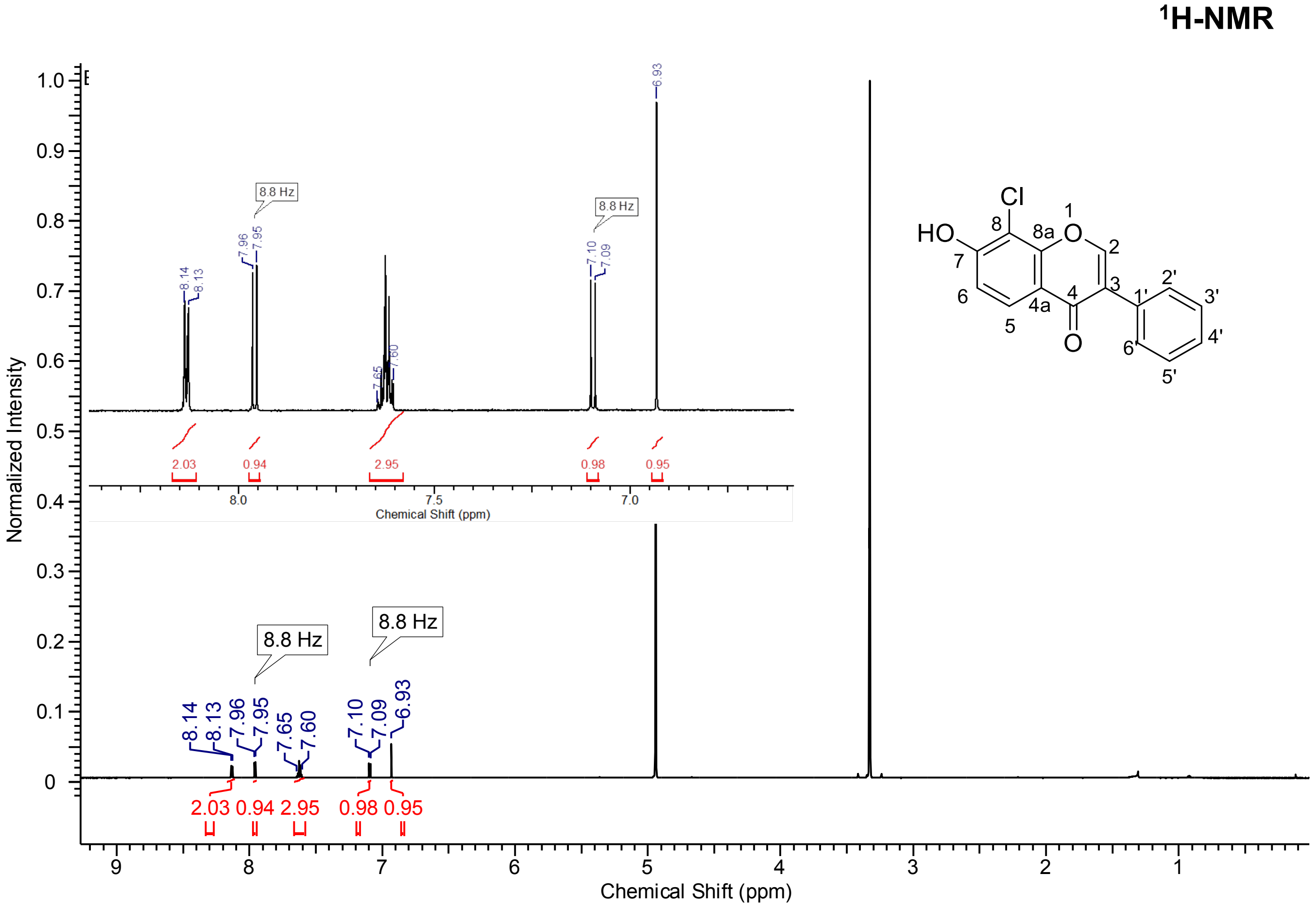

HPLC trace, UV spectrum and ^{1}H NMR of 8-chloro-7-hydroxy-3-phenyl-4H-chromen-4-on (**12a**)

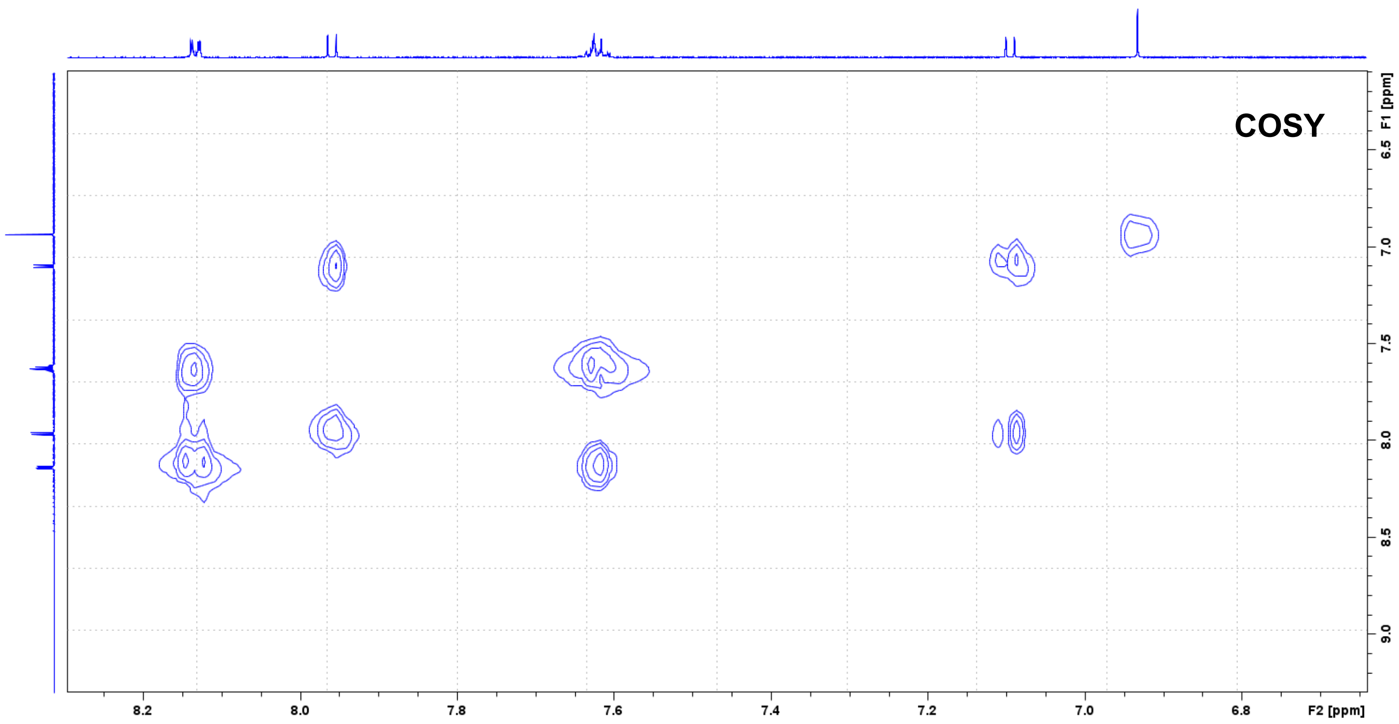

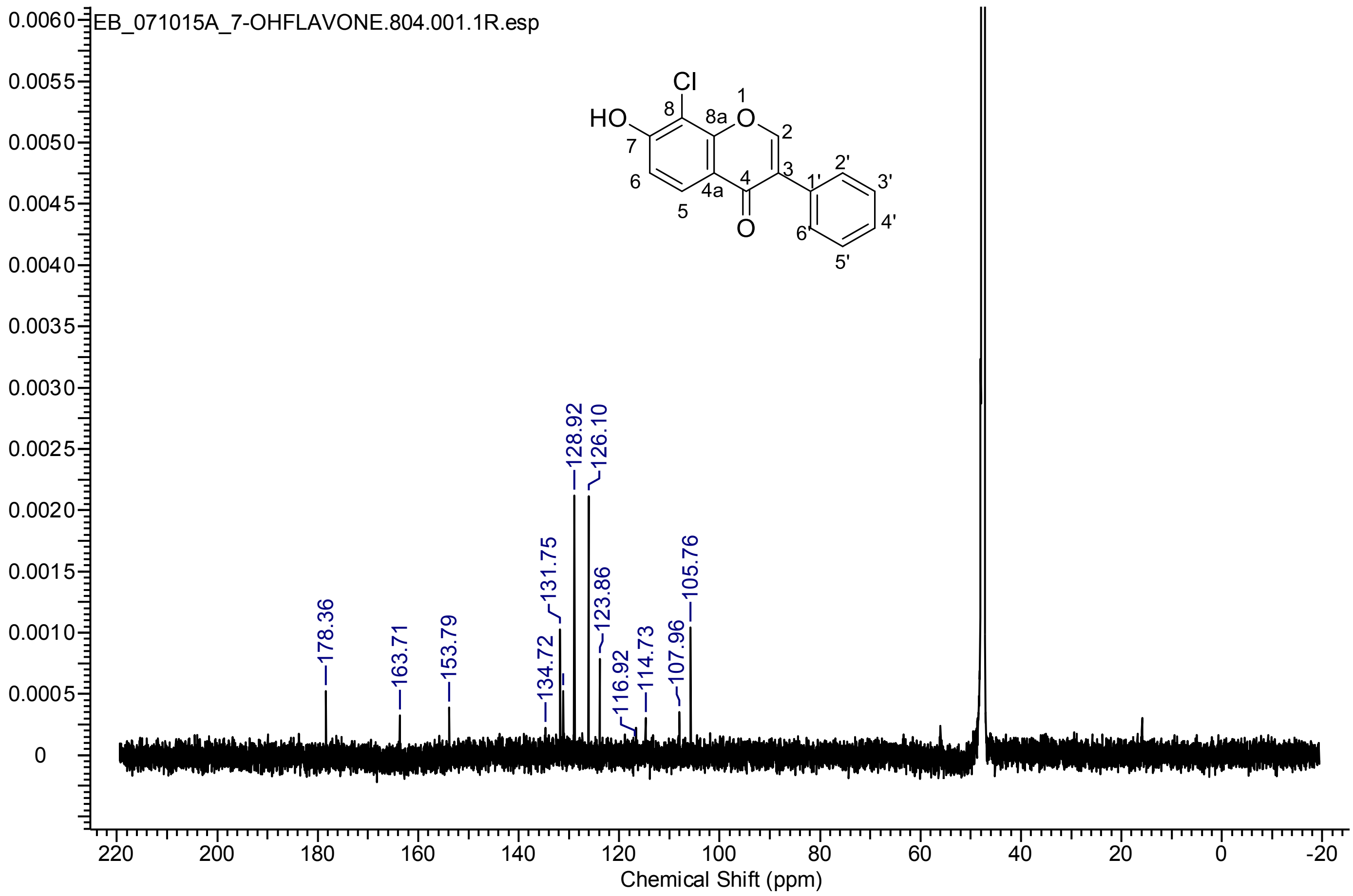

COSY and ^{13}C NMR of 8-chloro-7-hydroxy-3-phenyl-4H-chromen-4-on (**12a**).

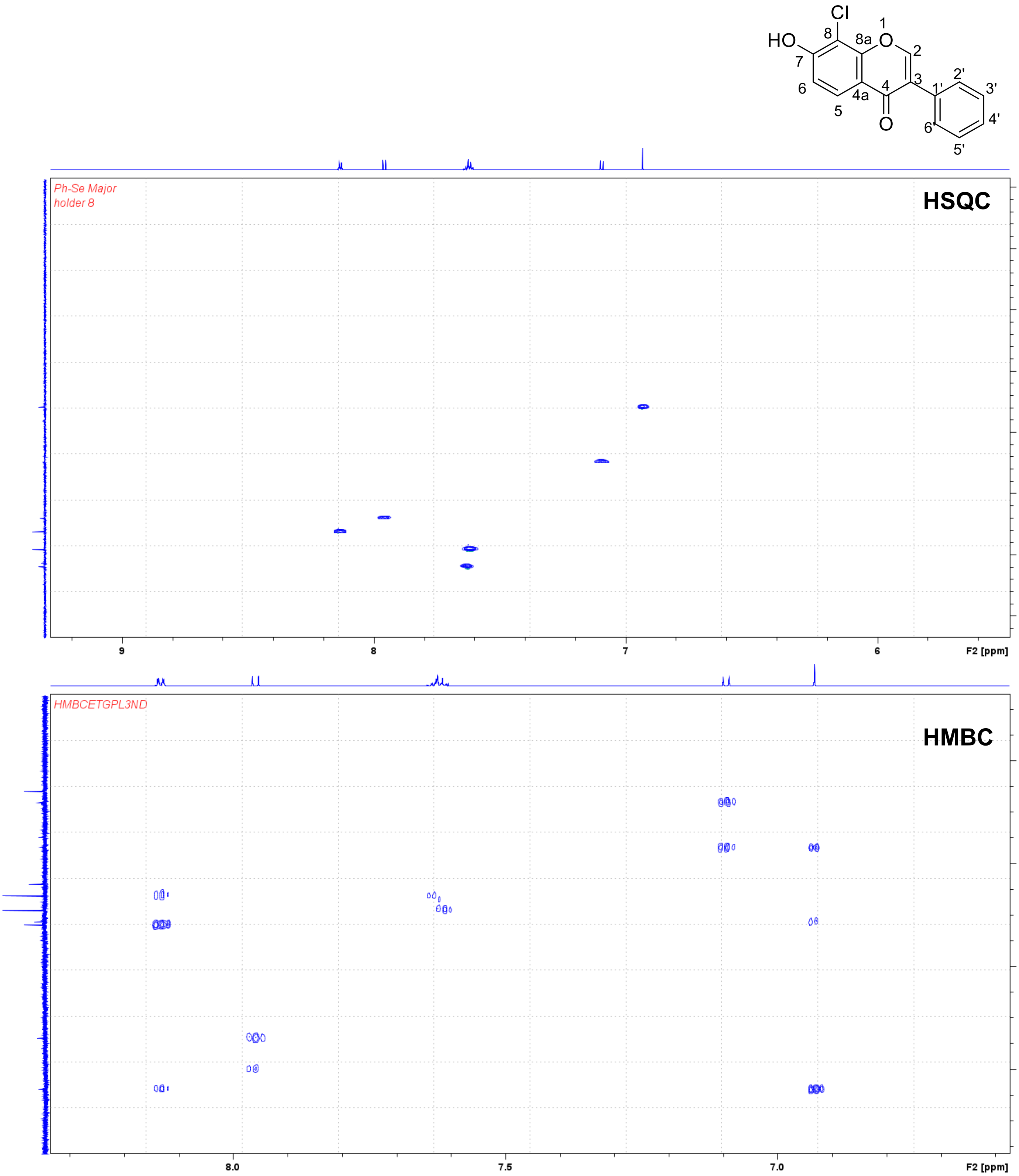

HSQC and HMBC of 8-chloro-7-hydroxy-3-phenyl-4H-chromen-4-on (**12a**).

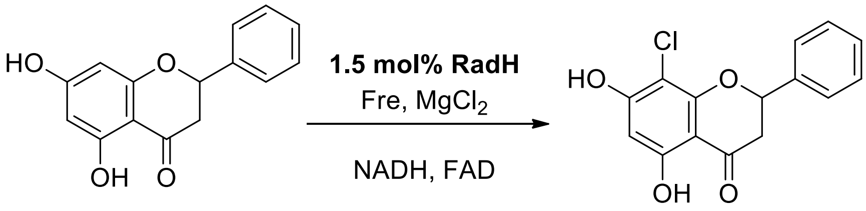

13 **13a**

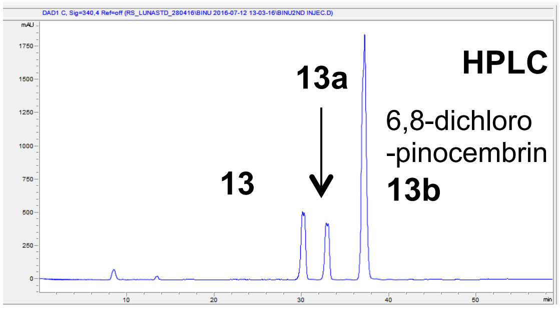

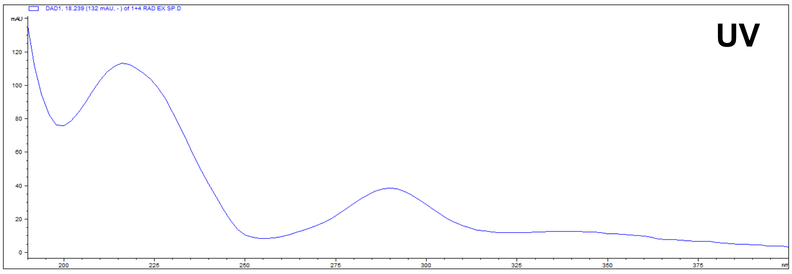

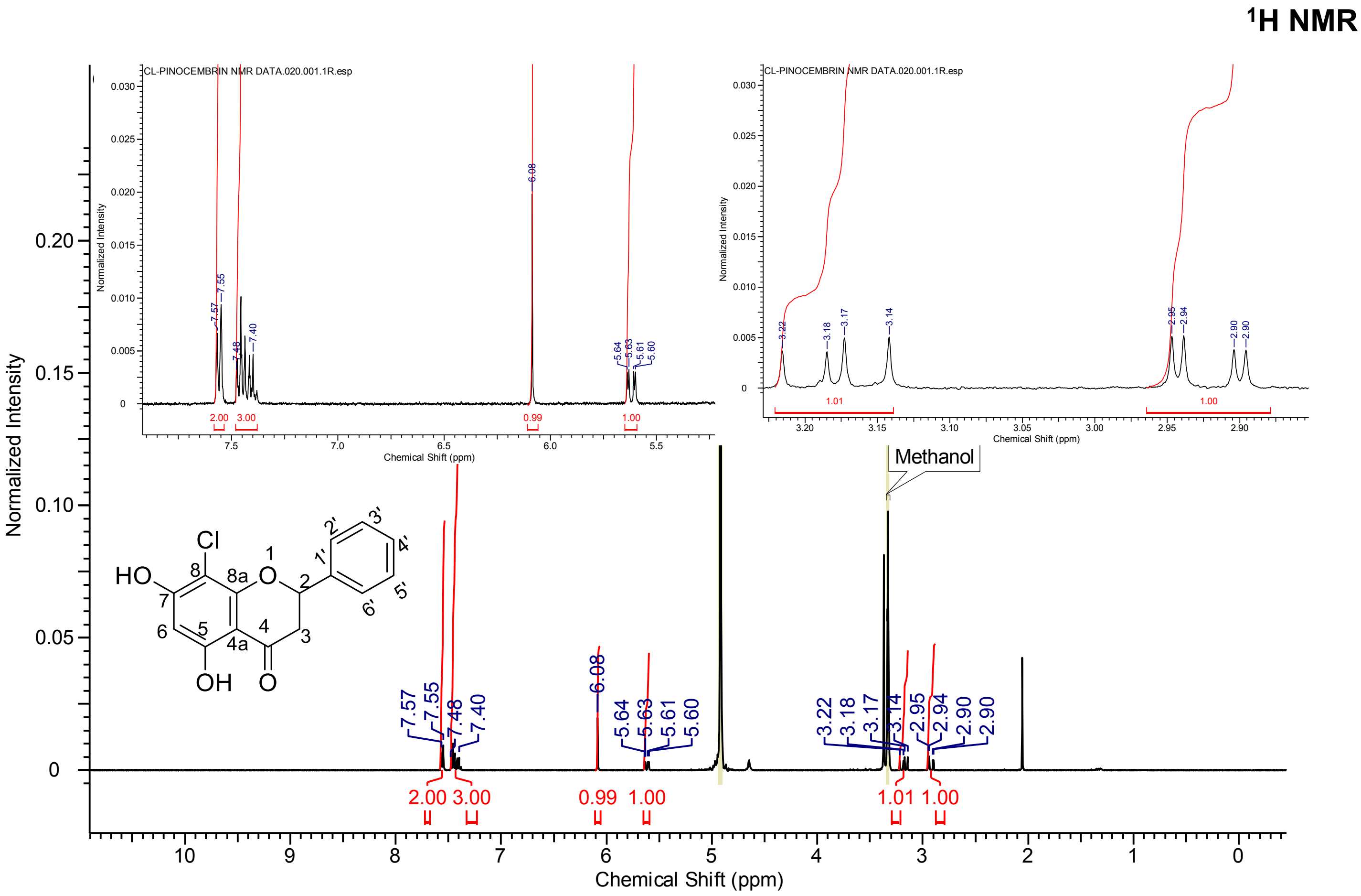

HPLC trace, UV spectrum and ^{1}H NMR of 8-Chloro- 5,7-dihydroxy-2-phenylchroman-4-one (8-chloropinocembrin) (**13a**).

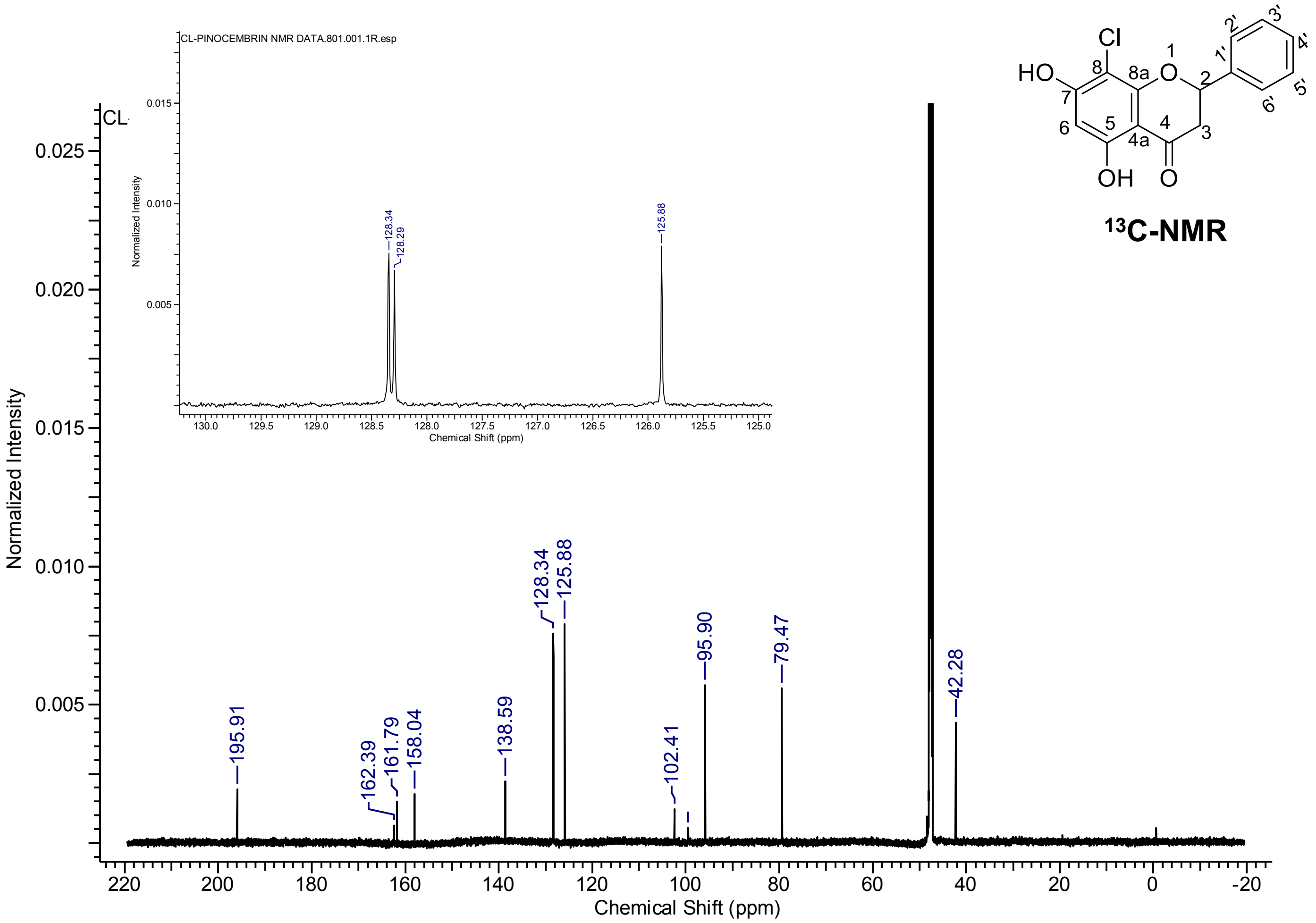

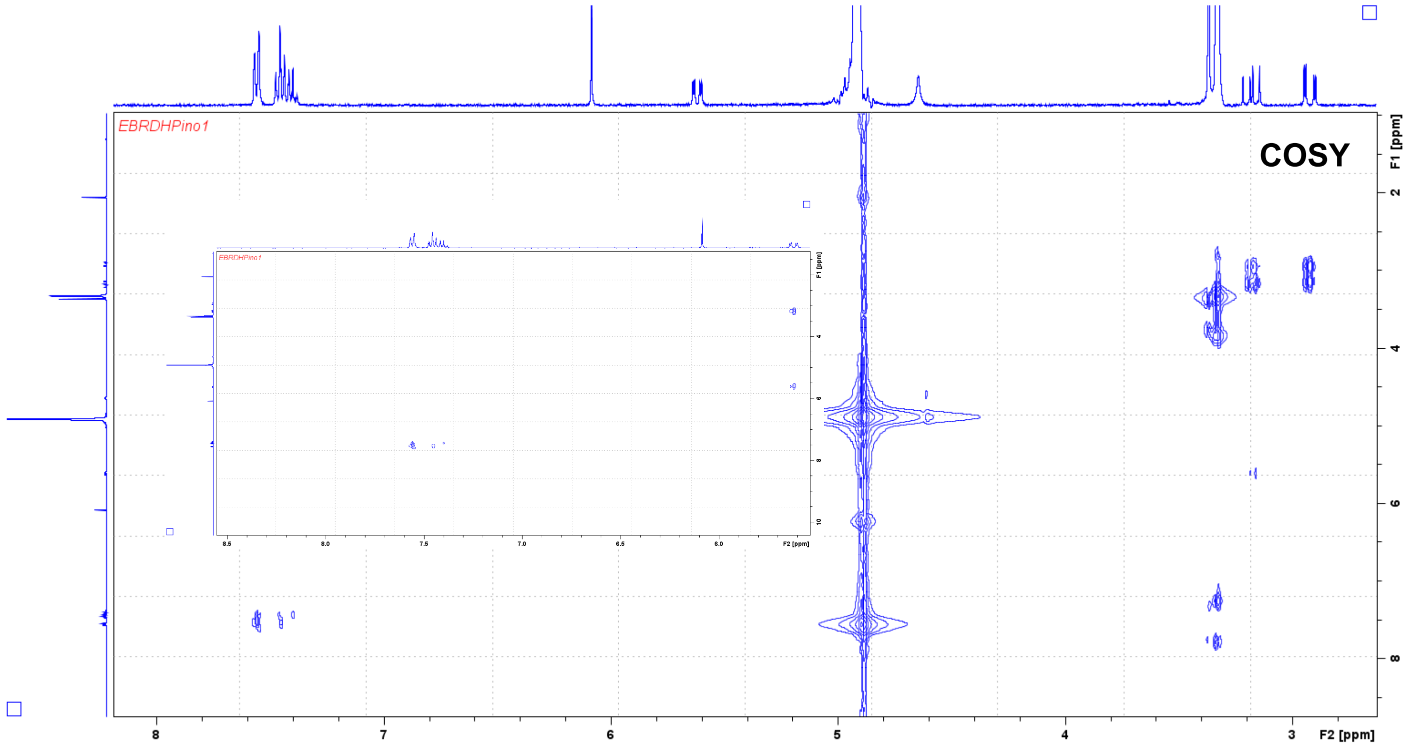

^{13}C NMR and COSY of 8-Chloro- 5,7-dihydroxy-2-phenylchroman-4-one (8-chloropinocembrin) (**13a**).

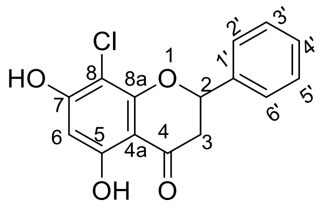

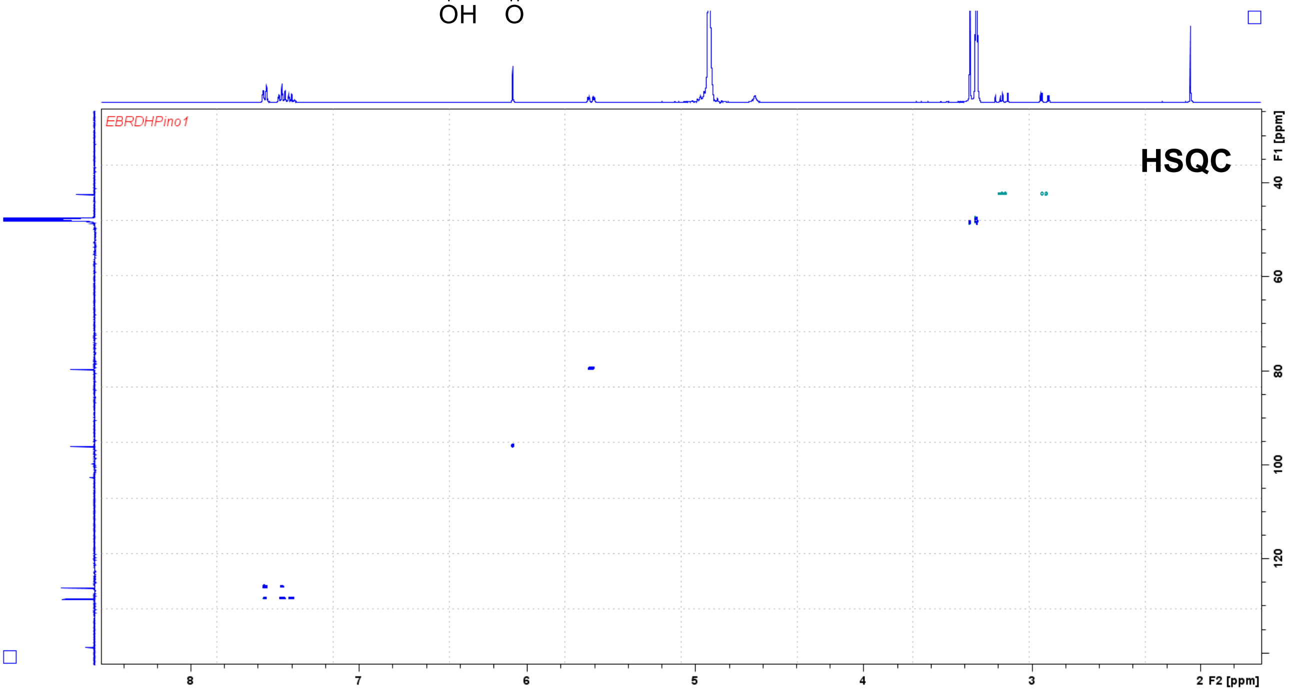

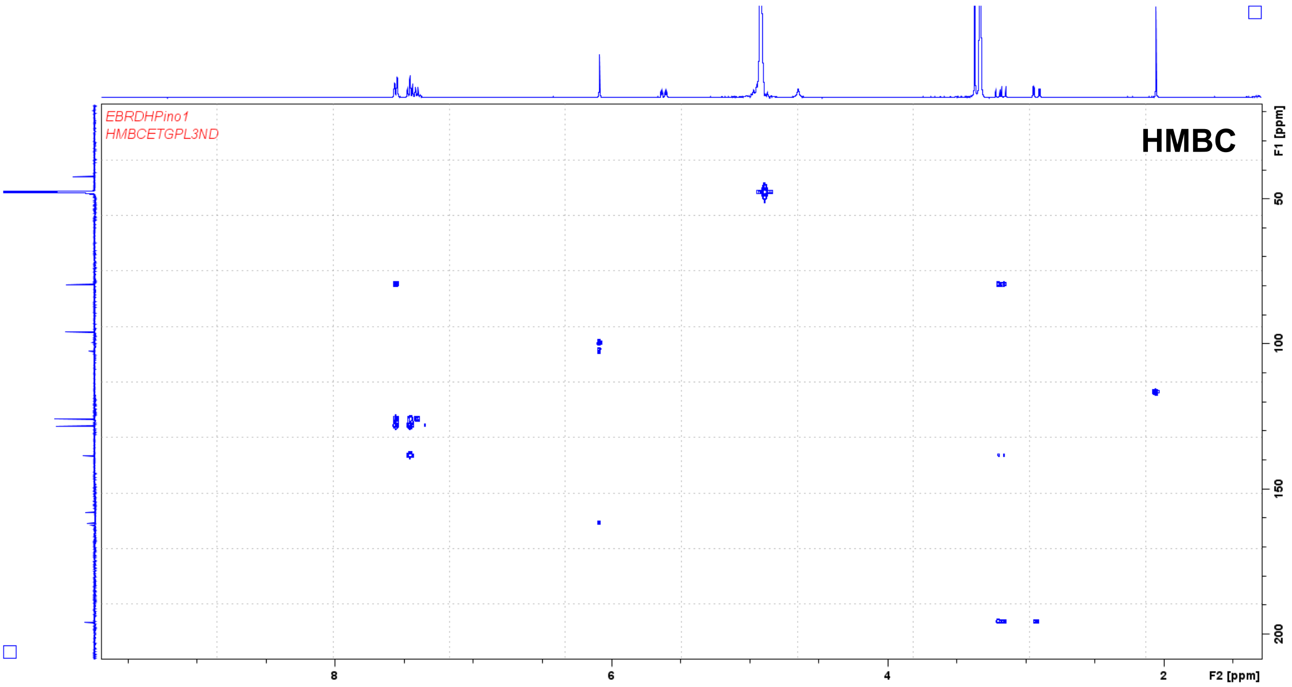

HSQC and HMBC of 8-Chloro- 5,7-dihydroxy-2-phenylchroman-4-one (8-chloropinocembrin) (**13a**).

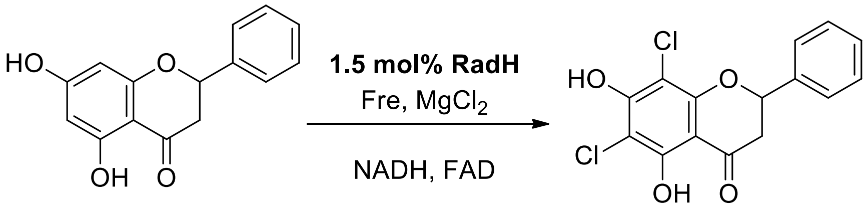

13 **13c**

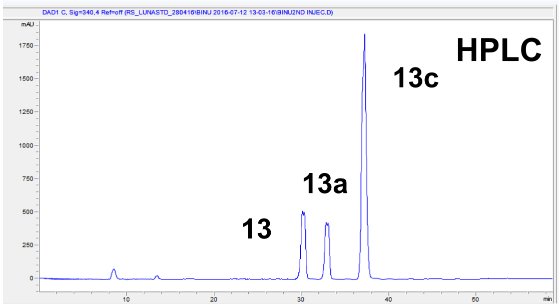

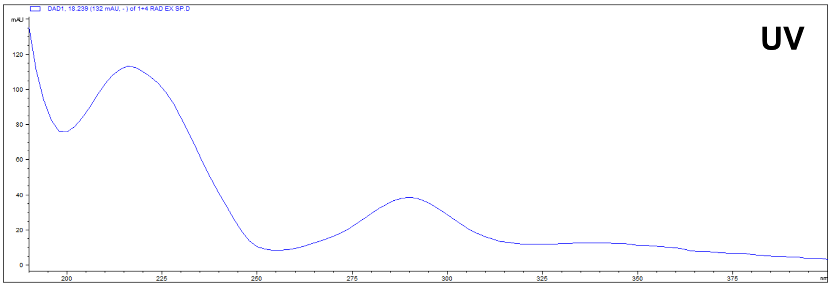

^{1}H NMR

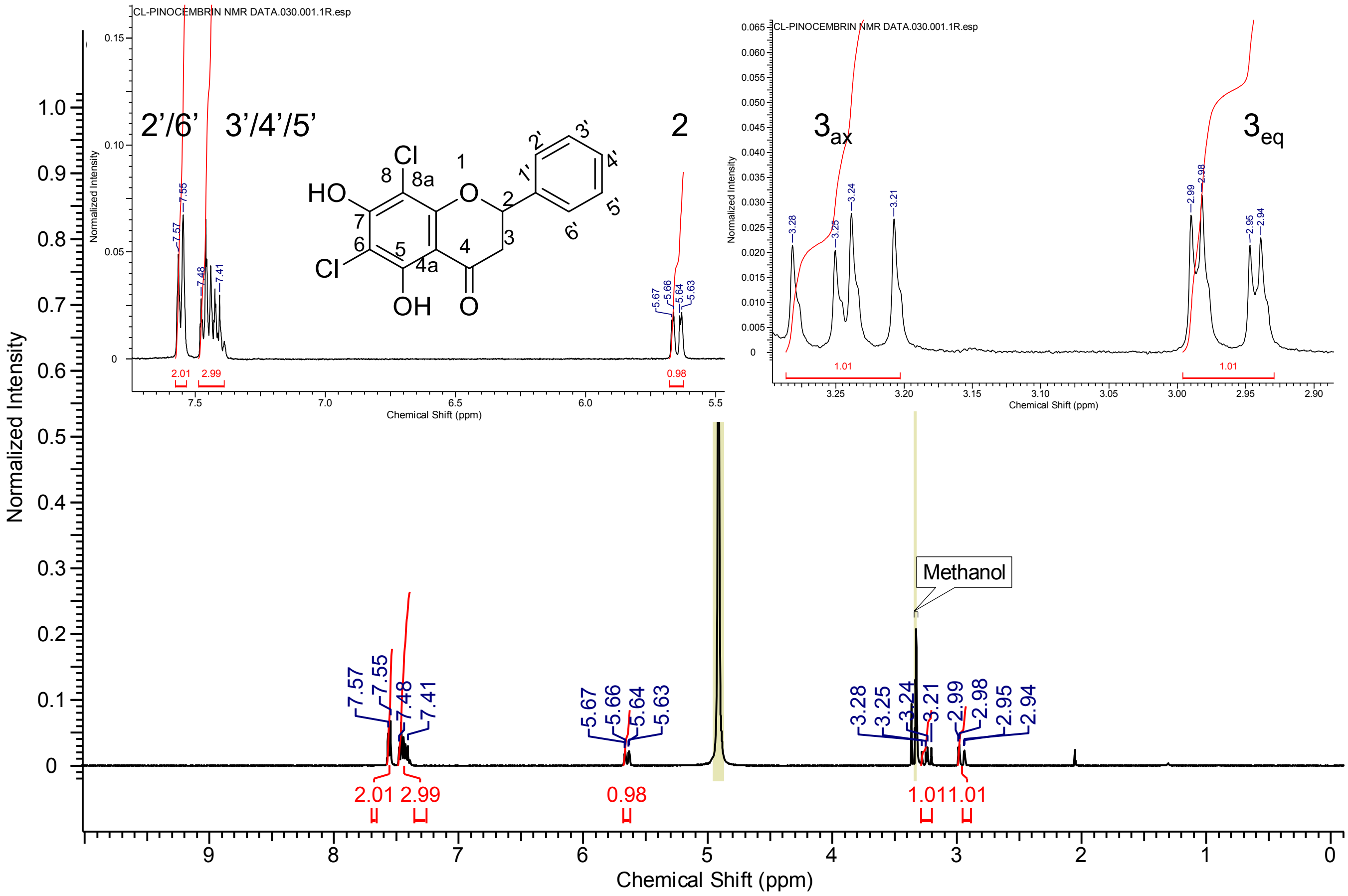

HPLC trace, UV spectrum and ^{1}H NMR of 6,8-dichloro- 5,7-dihydroxy-2-phenylchroman-4-one (6,8-dichloropinocembrin) (**13c**).

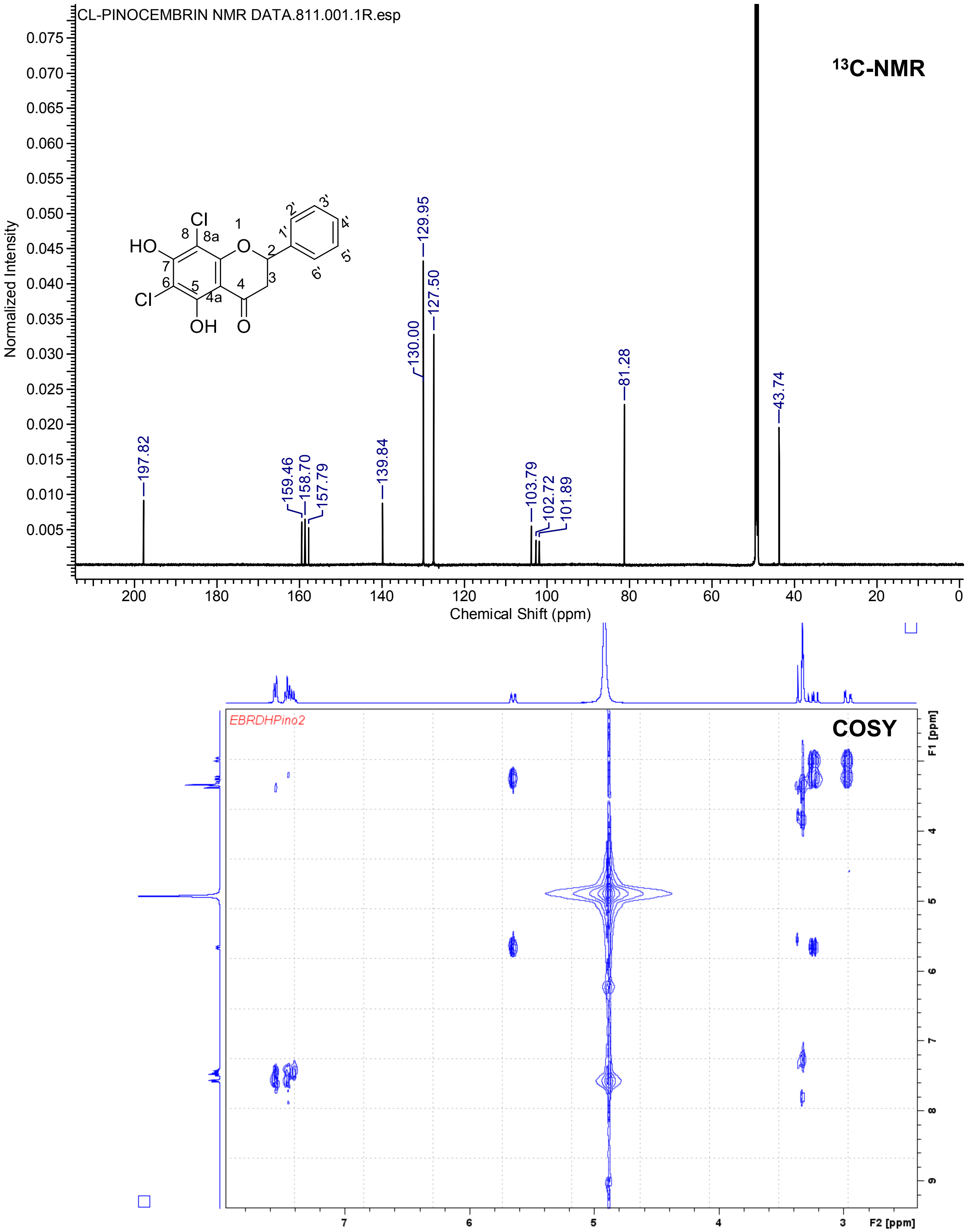

^{13}C NMR and COSY of 6,8-dichloro- 5,7-dihydroxy-2-phenylchroman-4-one (6,8-dichloropinocembrin) (**13c**).

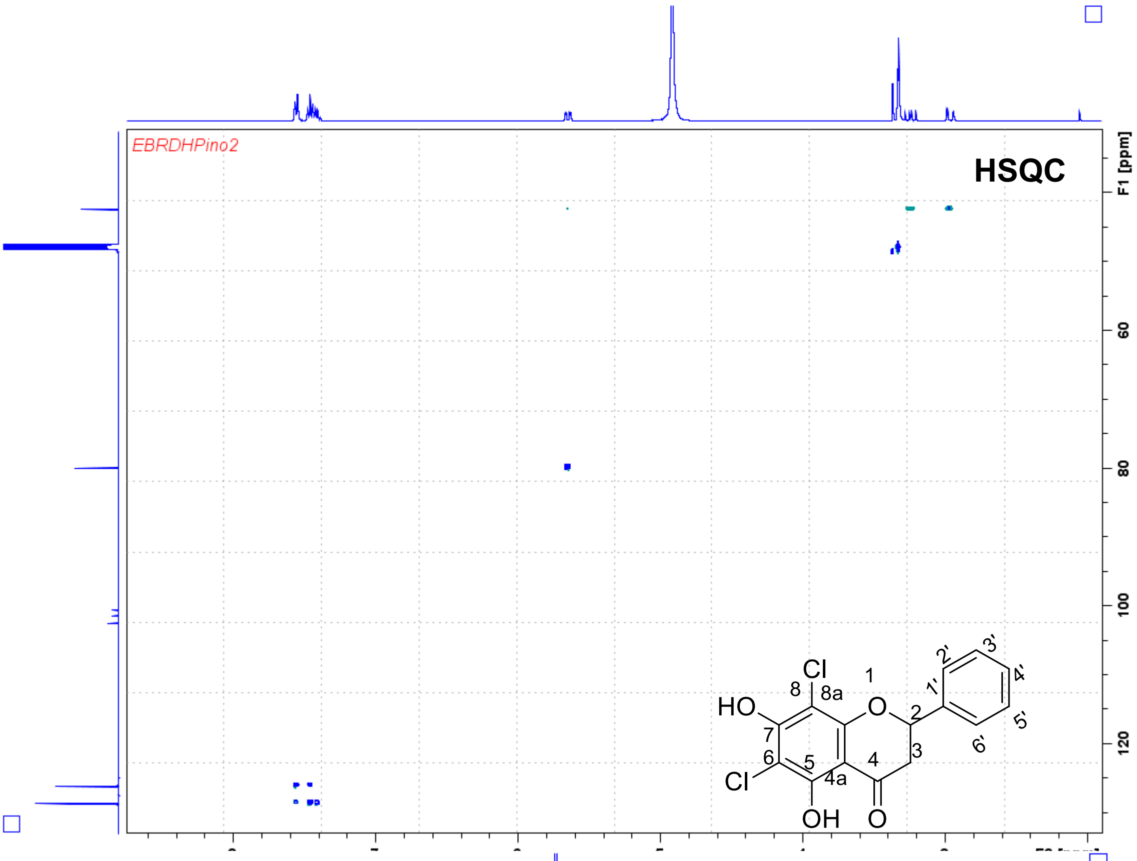

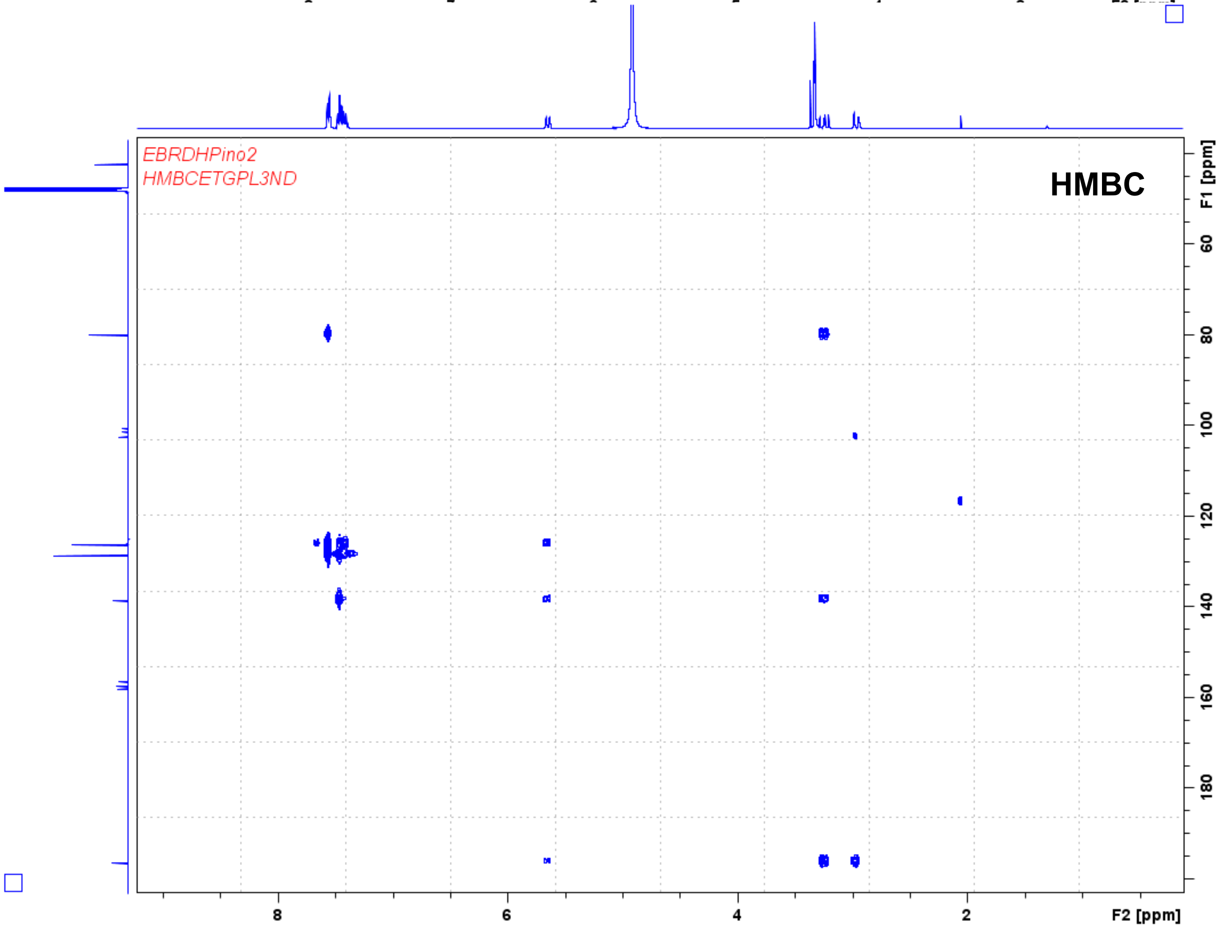

HSQC and HMBC of 6,8-dichloro- 5,7-dihydroxy-2-phenylchroman-4-one (6,8-dichloropinocembrin) (**13c**).

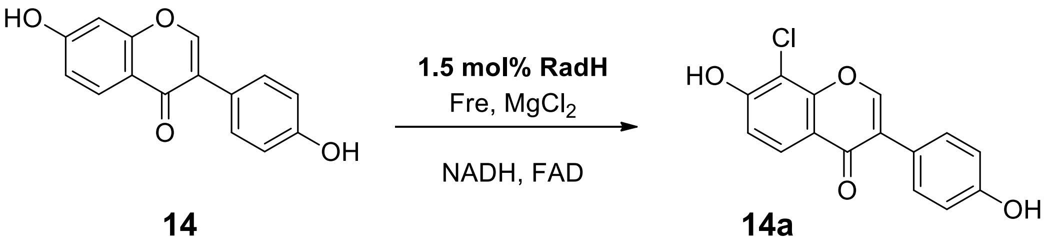

14 **14a**

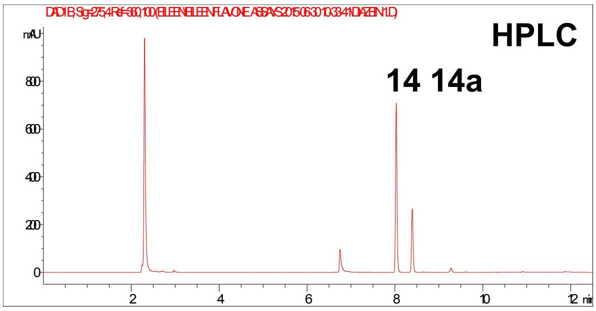

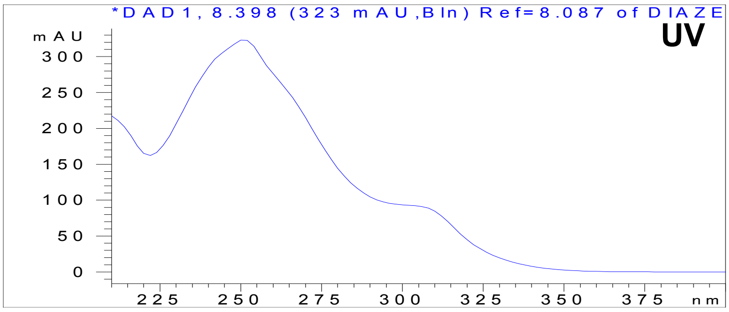

^{1}H NMR

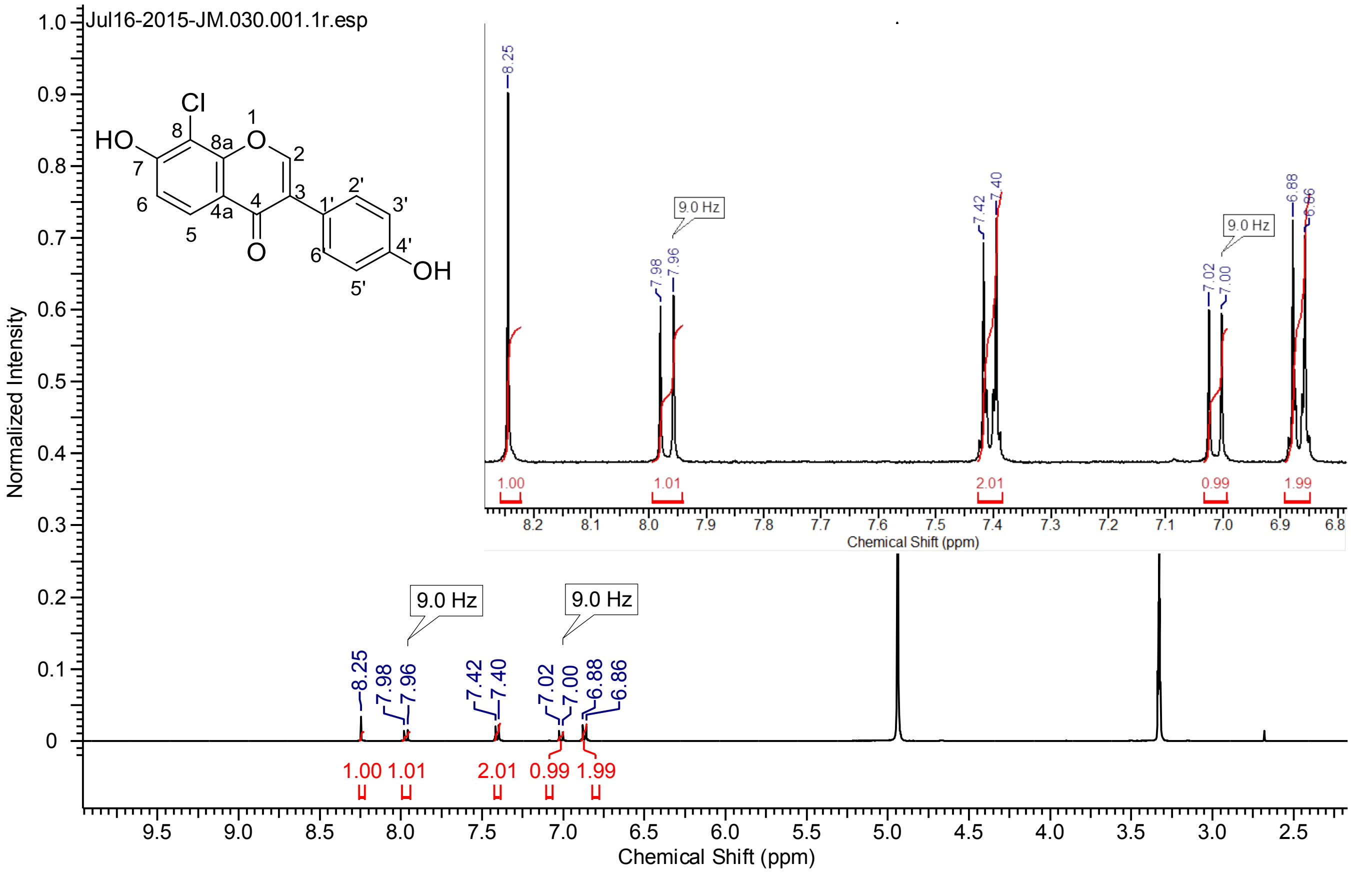

HPLC trace, UV spectrum and ^{1}H NMR of 8-chloro-7-hydroxy-3-(4-hydroxyphenyl)-4H-chromen-4-one (**14a**).

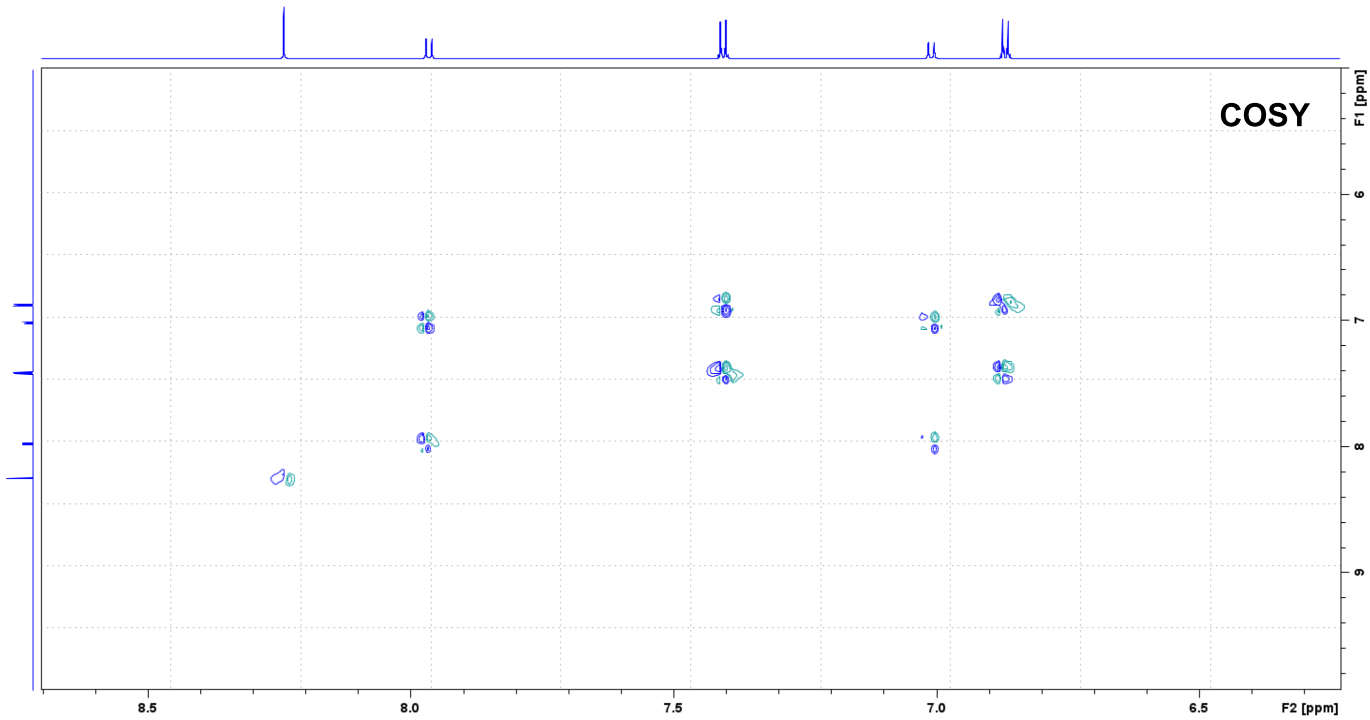

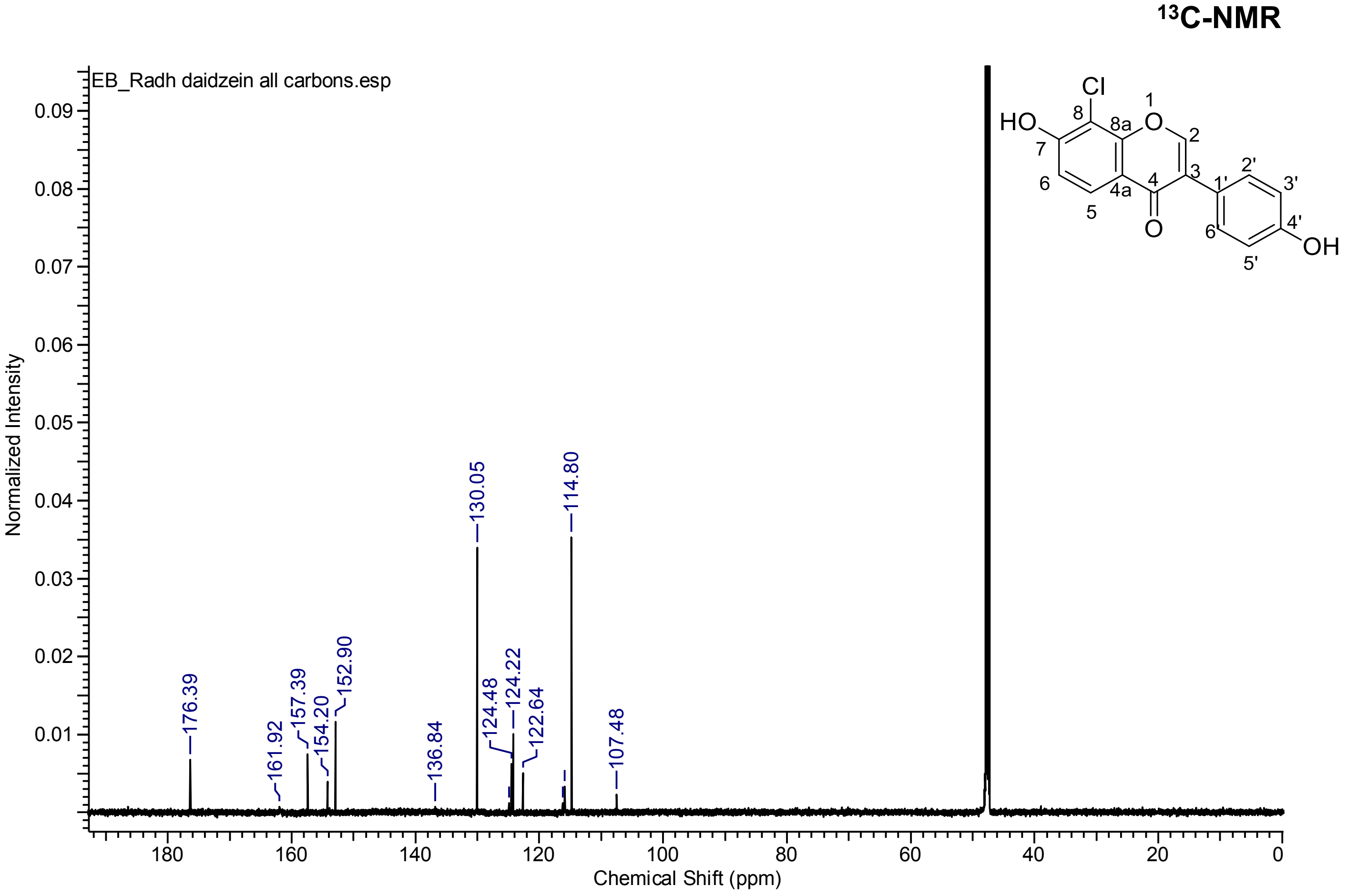

COSY and ^{13}C NMR of 8-chloro-7-hydroxy-3-(4-hydroxyphenyl)-4H-chromen-4-one (**14a**).

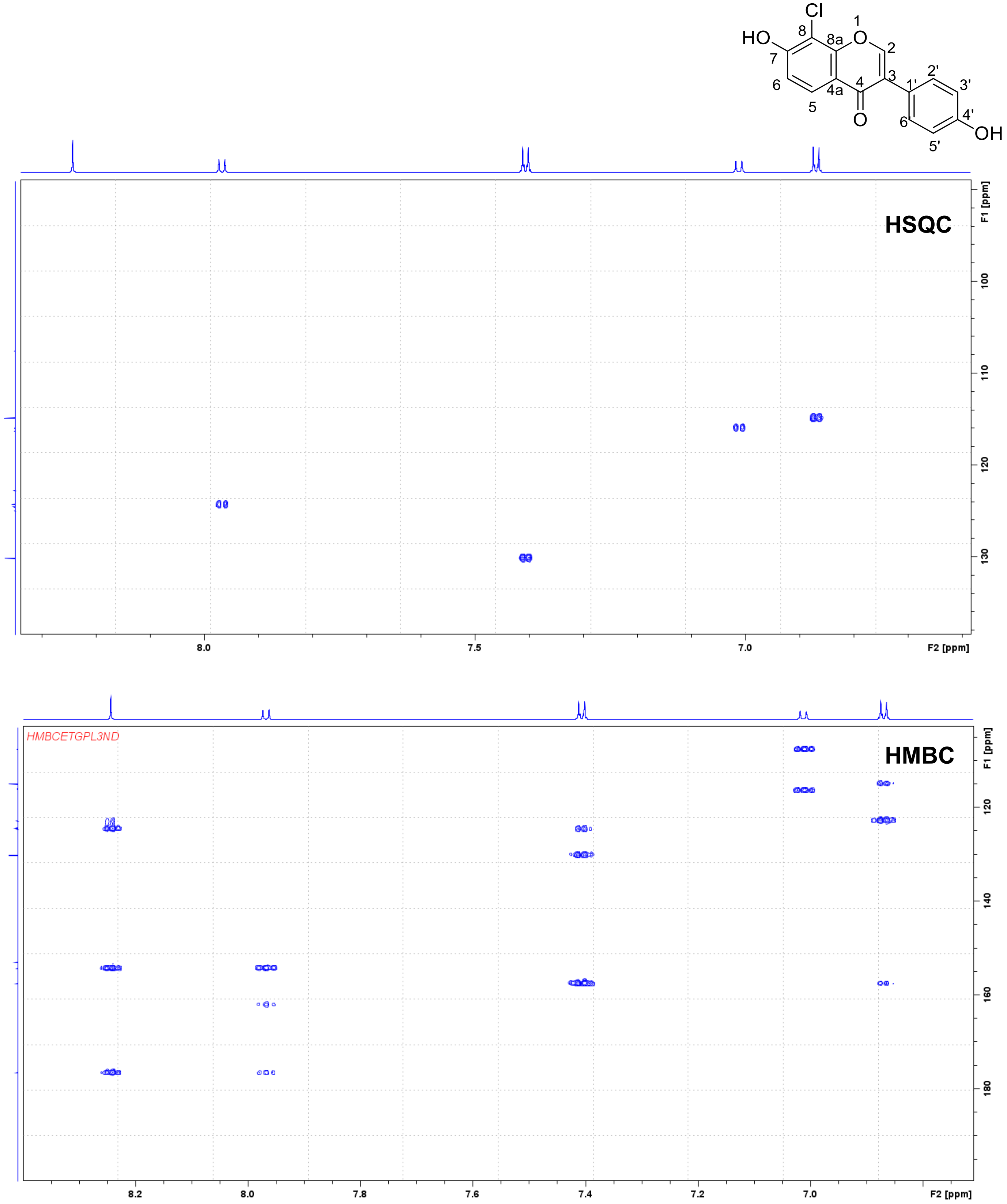

HSQC and HMBC of 8-chloro-7-hydroxy-3-(4-hydroxyphenyl)-4H-chromen-4-one (**14a**).

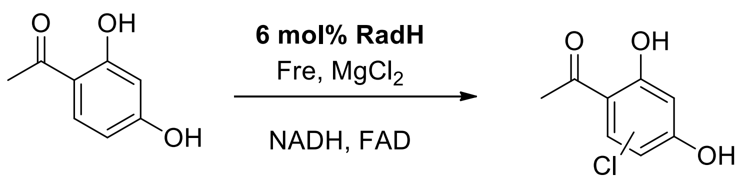

15 → **15a/15b**

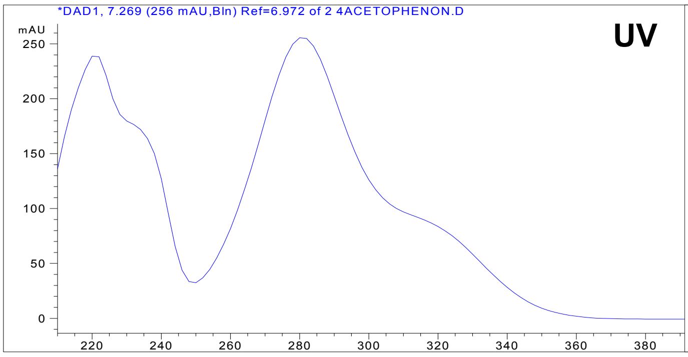

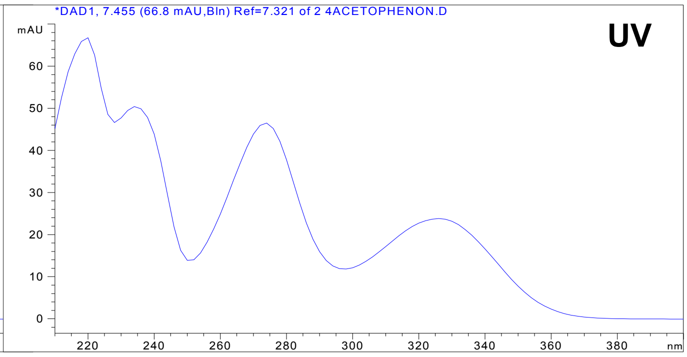

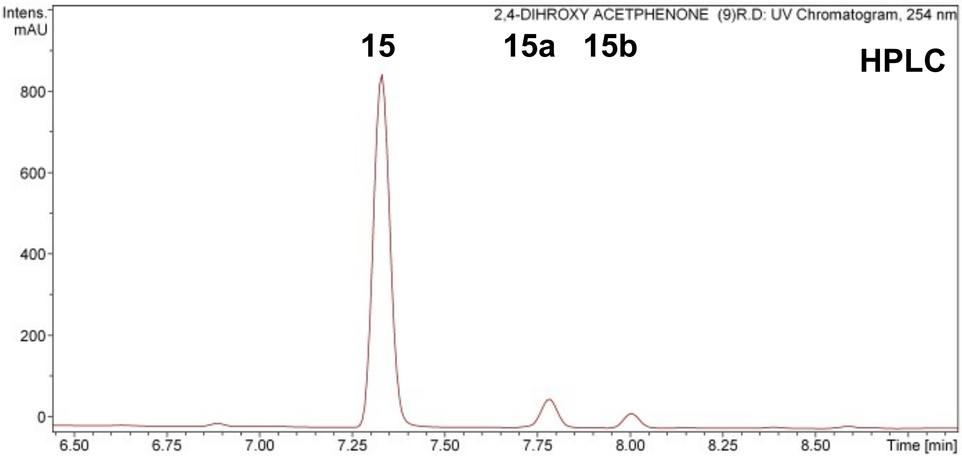

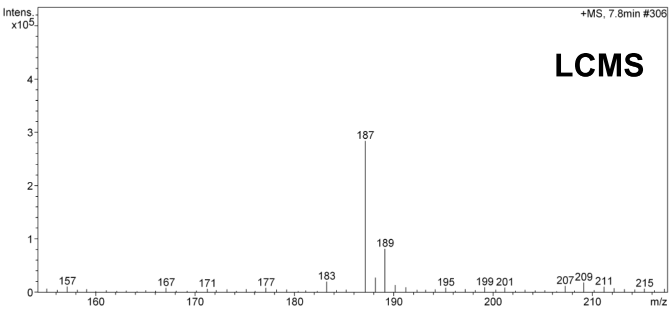

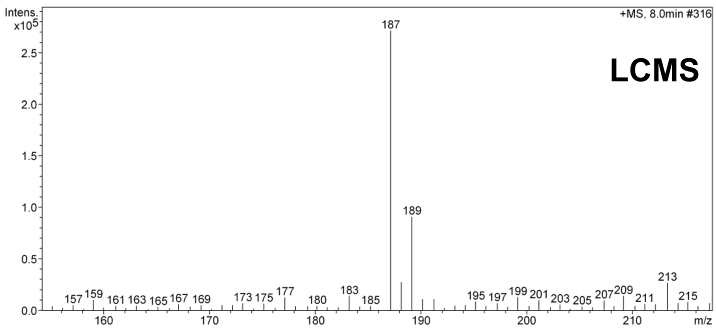

UV spectra, HPLC trace and LCMS of 1-(5-chloro-2,4-dihydroxyphenyl)ethanone (**15a**) and 1-(3-chloro-2,4-dihydroxyphenyl)ethanone (**15b**).

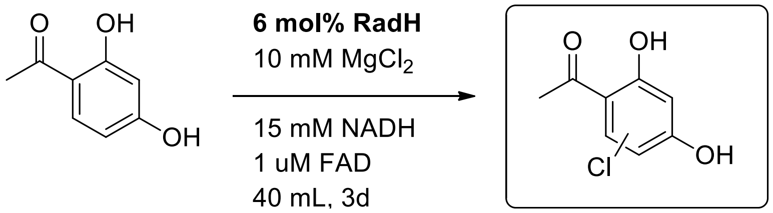

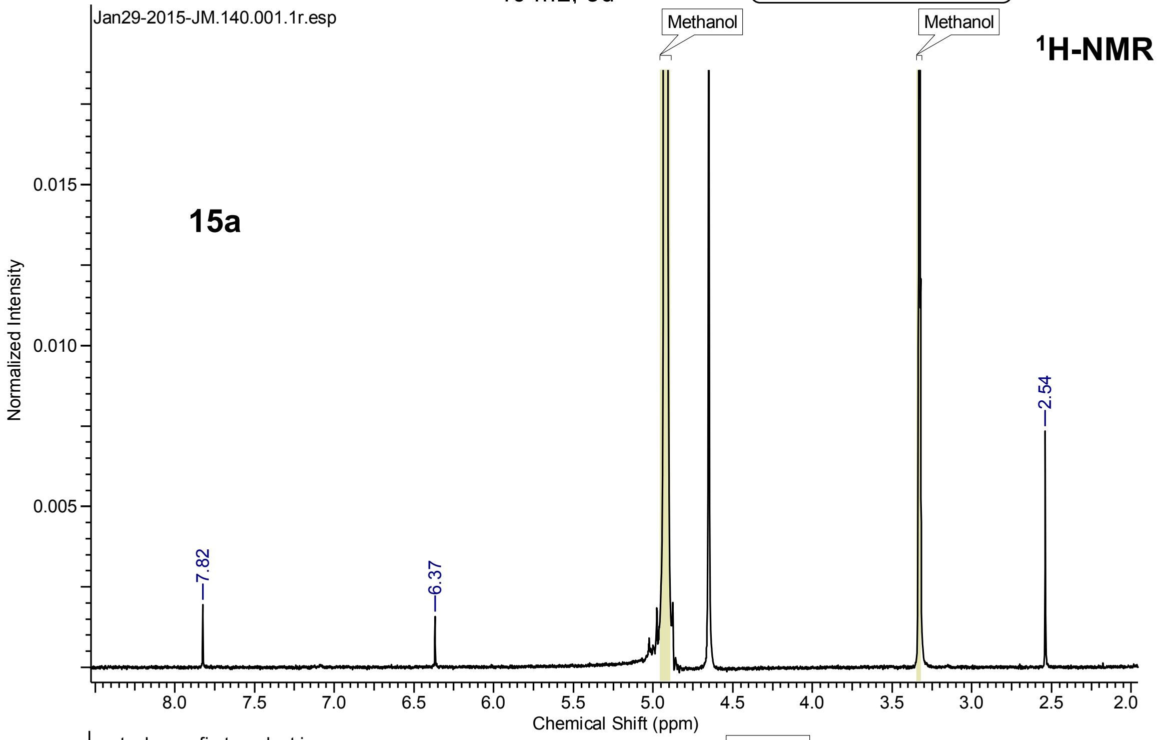

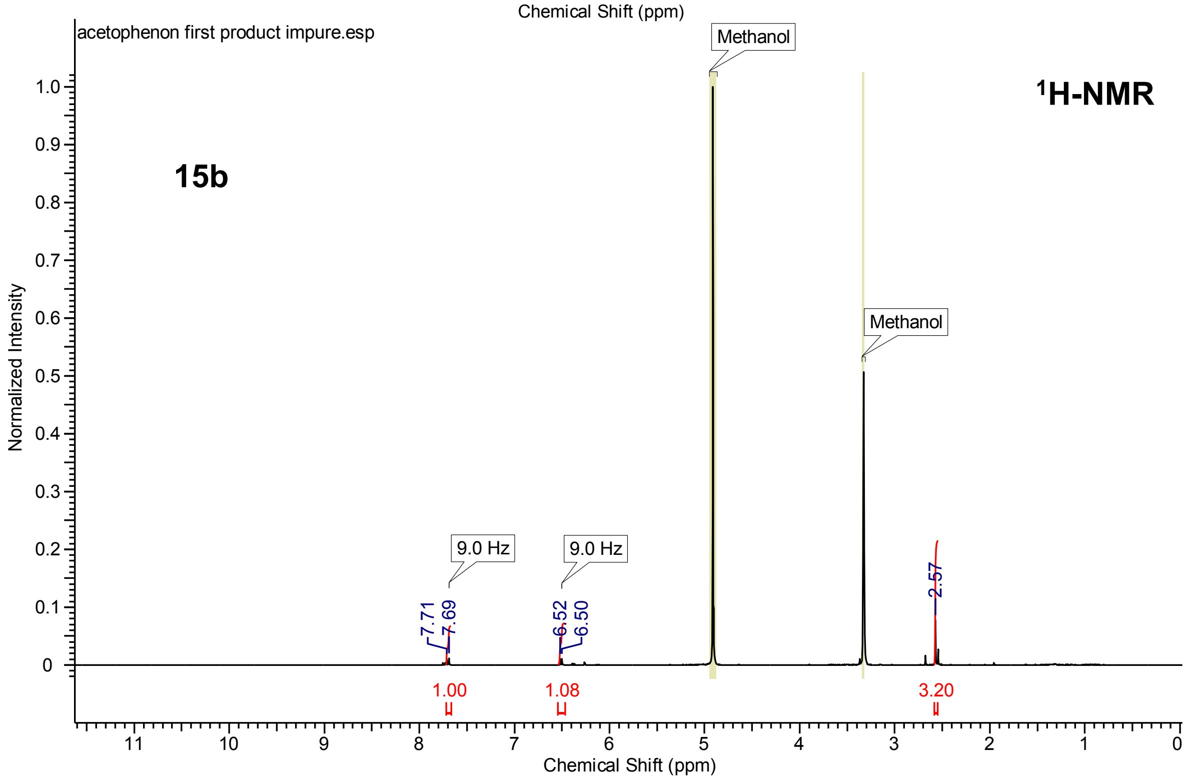

^{1}H NMR of 1-(5-chloro-2,4-dihydroxyphenyl)ethanone (**15a**) and 1-(3-chloro-2,4-dihydroxyphenyl)ethanone (**15b**).
